# Accurate non-covalent interaction energies on noisy intermediate-scale quantum computers *via* second-order symmetry-adapted perturbation theory†

**DOI:** 10.1039/d2sc05896k

**Published:** 2023-02-23

**Authors:** Matthias Loipersberger, Fionn D. Malone, Alicia R. Welden, Robert M. Parrish, Thomas Fox, Matthias Degroote, Elica Kyoseva, Nikolaj Moll, Raffaele Santagati, Michael Streif

**Affiliations:** a QC Ware Corporation, Palo Alto CA 94301 USA rob.parrish@qcware.com; b Medicinal Chemistry, Boehringer Ingelheim Pharma GmbH & Co. KG Birkendorfer Straße 65 88397 Biberach an der Riß Germany; c Quantum Lab, Boehringer Ingelheim 55218 Ingelheim am Rhein Germany nikolaj.moll@boehringer-ingelheim.com

## Abstract

The calculation of non-covalent interaction energies on noisy intermediate-scale quantum (NISQ) computers appears to be challenging with straightforward application of existing quantum algorithms. For example, the use of the standard supermolecular method with the variational quantum eigensolver (VQE) would require extremely precise resolution of the total energies of the fragments to provide for accurate subtraction to the interaction energy. Here we present a symmetry-adapted perturbation theory (SAPT) method that may provide interaction energies with high quantum resource efficiency. Of particular note, we present a quantum extended random-phase approximation (ERPA) treatment of the SAPT second-order induction and dispersion terms, including exchange counterparts. Together with previous work on first-order terms (*Chem. Sci.*, 2022, **13**, 3094), this provides a recipe for complete SAPT(VQE) interaction energies up to second order, which is a well established truncation. The SAPT interaction energy terms are computed as first-level observables with no subtraction of monomer energies invoked, and the only quantum observations needed are the VQE one- and two-particle density matrices. We find empirically that SAPT(VQE) can provide accurate interaction energies even with coarsely optimized, low circuit depth wavefunctions from a quantum computer, simulated through ideal statevectors. The errors of the total interaction energy are orders of magnitude lower than the corresponding VQE total energy errors of the monomer wavefunctions. In addition, we present heme-nitrosyl model complexes as a system class for near term quantum computing simulations. They are strongly correlated, biologically relevant and difficult to simulate with classical quantum chemical methods. This is illustrated with density functional theory (DFT) as the predicted interaction energies exhibit a strong sensitivity with respect to the choice of functional. Thus, this work paves the way to obtain accurate interaction energies on a NISQ-era quantum computer with few quantum resources. It is the first step in alleviating one of the major challenges in quantum chemistry, where in-depth knowledge of both the method and system is required *a priori* to reliably generate accurate interaction energies.

## Abstract 2

Proposed quantum algorithm for accurate non-covalent interactions on noisy intermediate-scale quantum computers. Combining molecular wavefunctions from the quantum computing and classical post procressing using symmetry-adapted perturbation theory.

## Graphical abstract

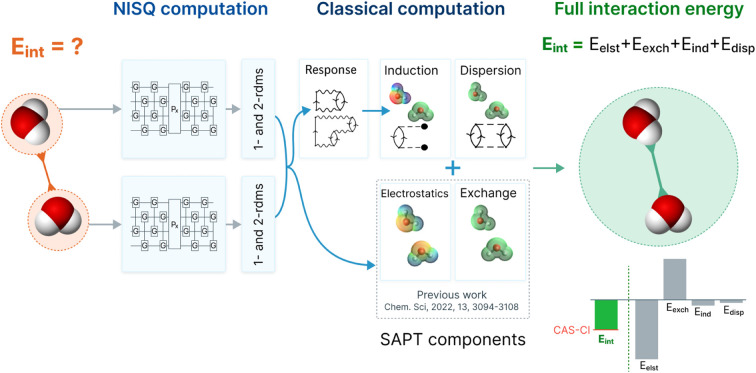

## 1. Introduction

Quantum computing has emerged as a promising platform to approach classically challenging problems in chemistry.^1,2^ The most interesting near-term application is the simulation of strongly correlated systems for which the electronic structure cannot be described with a single Slater determinant. For such systems Kohn–Sham density functional theory^3,4^ (KS-DFT) may fail to describe the electronic structure correctly; popular examples are Fe–S clusters or the FeMo-cofactor.^5,6^

Classically, a proper treatment of these strongly correlated systems is achieved with multi-reference methods where the naive combinatorial scaling of the wavefunction ansatz limits its applications. Note however that much progress has been made on classical heuristics for wavefunction methods that exhibit less than combinatorial scaling and that may be highly accurate for a broad range of problems.^7–13^ Alternatively, quantum algorithms^2,14–16^ might be used to solve the Schrödinger equation with a resource cost that scales polynomially with the number of qubits. Unfortunately, the currently available noisy intermediate-scale quantum (NISQ) hardware^17^ suffers from relatively poor gate fidelity and a low qubit count,^18^ which poses two key challenges. First, it is important for NISQ-tailored quantum algorithms^19^ to minimize quantum resources. The most prominent NISQ methods are hybrid quantum-classical algorithms like the variational quantum eigensolver (VQE),^20,21^ quantum Krylov methods,^18,22–26^ or the fermionic quantum Monte Carlo method.^27^ The second challenge is to find specific applications that could harness quantum computing.^28,29^ Many application studies in chemistry use either reduced model systems or molecules with a simple electronic structure.^30–33^ There are several promising application areas for quantum chemistry in computer aided drug design^34^ namely, exploring potential energy surfaces,^35^ simulating metalloenzymes^36^ and computing protein-ligand interaction energies,^37^ the last of which we consider in this work.

The computation of non-covalent interaction energies is a routine task in classical quantum chemistry^38,39^ and the standard procedure is the supermolecular approach: the interaction energy is calculated as the difference between the ground state energies of the dimer and two monomers separated to infinity.^40^ However, transferring this approach to a NISQ type quantum computer is difficult for several reasons: first, the VQE total energies (on the order of thousands of kcal mol^−1^) need to be tightly converged to resolve interaction energies on the order a few kcal mol^−1^ with the supermolecular approach. This is a disadvantage on NISQ hardware because the total energy expectation value is obtained statistically and high-precision expectation values require a high number of measurements. Furthermore, converging the VQE total energy to high accuracy requires deep circuits associated with a large set of parameters, where it becomes increasingly difficult to reach the global minimum on the parameter surface.^41^ Second, accounting for the basis set superposition error (BSSE) in the supermolecular approach^40^ is commonly achieved by expanding the basis in the monomer calculations to the size of the dimer basis. This unnecessarily increases the qubit count requirements for the individual monomers and can potentially lead to convergence issues for the VQE.^20,21^

To this end, this work provides an alternative pathway towards interaction energies with high accuracy and low quantum resource requirements by using symmetry-adapted perturbation theory (SAPT)^42–44^ in combination with VQE monomer wave functions [SAPT(VQE)]. This approach directly computes the interaction energy as a sum of small expectation values; in contrast, the supermolecular approach computes the small interaction energy (several kcal mol^−1^) as a difference of large total energies (thousands of kcal mol^−1^). This work builds on our previous work,^37^ where we presented the implementation of the first-order SAPT(VQE) terms of electrostatics and exchange. However, the first-order SAPT(VQE) terms alone are not capable of computing accurate interaction energies – standard levels of SAPT also require the computation of the second-order induction and dispersion terms.^43^ Up to now, the absence of a SAPT(VQE) recipe for the complete second-order SAPT terms has been a major potential weakness of the approach – indeed, other authors^30^ have noted that “[first-order SAPT(VQE)] computed interaction energies did not reproduce ligand rankings yielded by more accurate 2nd order SAPT calculations, due to the missing induction and dispersion components in the 1st order approximation”, and conclude that “[the first-order SAPT(VQE)] workflow is limited by truncation of the SAPT expansion at 1st order and it is not clear how their method can be effectively extended to higher orders”.

**Fig. 1:**
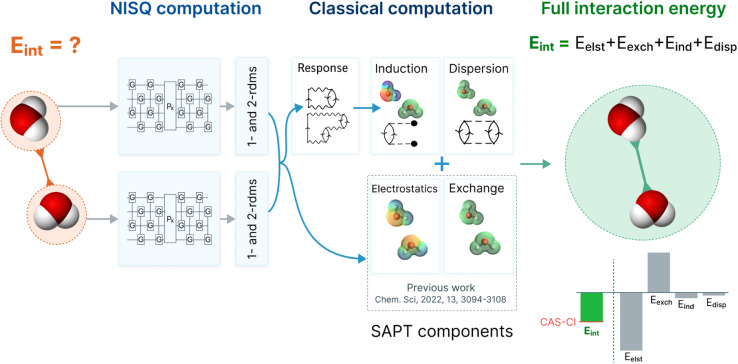
The workflow of SAPT(VQE) to obtain accurate interaction energies using a NISQ quantum algorithm: first, we define two monomers and the interaction of interest (exemplary here: two water molecules); second, we define the active space of the electrons which are simulated on the quantum computer; third, the quantum computer is used to compute the electronic structure *via* a quantum algorithm such as the VQE. The converged wavefunction yields reduced one- and two-particle density matrices (1- and 2-RDM); fourth, the classical computer computes the interaction energy as a post-processing step using the RDMs *via* a sum of electrostatic, exchange, induction and dispersion energies. The former two terms were published by some of us previously,^37^ and the latter two are presented in this study and employ an extended random phase approximation (ERPA) formalism. They require solving response equations and are necessary to obtain accurate interaction energies; fifth, the SAPT energy components and interaction energies provide an in-depth understanding of intermolecular interaction of interest (note that in this work the quantum computing results were obtained on a simulator).

In this work, we ameliorate this crucial deficiency by direct implementation of the second-order induction and dispersion terms, together with their exchange counterparts. The combination of first order energies (from our previous work^37^) and second order energies (presented in this work) is an accurate truncation of the SAPT perturbation series.^43^ Those methods usually provide interaction energies with an accuracy below 1 kcal mol^−1^ for a large spectrum of non-covalent binding motifs, even with modest basis sets.^45,46^ Therefore, this work provides a pathway to obtain accurate intermolecular interaction energies using wavefunctions from quantum computations (in this work using VQE wavefunctions). The improved SAPT(VQE) workflow is depicted in Fig. 1 (using a water dimer as an example).

Our approach follows the SAPT(FCI) approach of Korona^47^ but with the naively exponential-scaling FCI piece replaced by an active-space VQE wavefunction, which is intended to be implemented on a forthcoming NISQ computer (in this work we use ideal statevector simulators for the numerical tests). To implement the second-order terms, we follow the extended random phase approximation (ERPA) formalism for SAPT(CASSCF) (complete active space self-consistent field (CASSCF)) developed by Hapka *et al.*,^48^ with the VQE standing in for the FCI solver in CASSCF. In the approach, the response equations for the coupled polarization propagators are carried out in a truncated hole-particle basis reminiscent of the Casida expansion^49^ in TD-DFT or the quantum subspace expansion (QSE) for VQE excited states.^50^ While this treatment necessarily does not include all Hamiltonian states even in the FCI limit, it does use a set of coupled hole-particle states that span the full range of energetic scales of the Hilbert space, which are empirically known to provide highly converged results for the induction and dispersion energies. Notably, the use of the ERPA formalism in SAPT(VQE) allows for the computation of the second-order induction and dispersion terms, together with exchange counterparts (here in the *S*^2^ approximation) with the active-space one- and two-particle density matrices of the VQE ground state wavefunctions appearing as the only “new” quantum observables. These observables are polynomial scaling and typically readily available as a byproduct of the VQE optimization procedure. The subsequent ERPA equations, induction and dispersion contractions, and exchange counterparts are polynomial scaling classical operations. They are significantly more complicated than the first-order terms – roughly 200 equations are needed to describe the implementation (see the ESI† for full details), and the naive CuPy^90^ implementation of the equations implemented here is restricted to smaller systems than our previous paper due to classical postprocessing overheads (though optimization techniques such as hole/active/particle separations and density fitting might significantly reduce these overheads). Complexity notwithstanding, the approach presented provides a recipe for a SAPT(VQE) doppelganger of SAPT(FCI) complete through all second order terms.

Below, we first lay out the motivation and high-level theory for the ERPA treatment of the second-order SAPT(VQE) approach. The ERPA equations, contractions to induction and dispersion terms, and exchange counterparts are straightforward but extremely verbose, so much of their explicit presentation is deferred to the ESI (Sections 1–3).† We then demonstrate the numerical performance of all four SAPT(VQE) terms and total interaction energies for several small multi-reference dimers and a model heme-nitrosyl hydrogen bonding complex, using classical ideal statevector simulators to emulate the VQE.

## 2. Theory

The most direct “supermolecular” route to the interaction energy of two monomers A and B is to simply compute the total energy of the combined system *E*_AB_ and subtract the total energies of its non-interacting constituents *E*_A_ + *E*_B_, *i.e.*,

1 *E*_int_ = *E*_AB_ − *E*_A_ − *E*_B_

ideally using the exact wave function for each system.

An alternative approach to computing intermolecular interaction energies is symmetry adapted perturbation theory (SAPT), which obtains the interaction energy with a different approach. In particular, we can write the Hamiltonian of the combined system as

2 *Ĥ* = *Ĥ*_A_ + *Ĥ*_B_ + *V̂*

where we assume *Ĥ*_X_|*Ψ*_X_〉 = *E*_X_|*Ψ*_X_〉, where |Ψ_X_〉 is the ground state wavefunction of monomer X and *V̂* contains only the coulombic interactions between monomers A and B. With this partitioning of the Hamiltonian we can build a perturbation theory for the intermolecular interaction energy directly, thus avoiding computing potentially very large total energies. More explicitly we have3
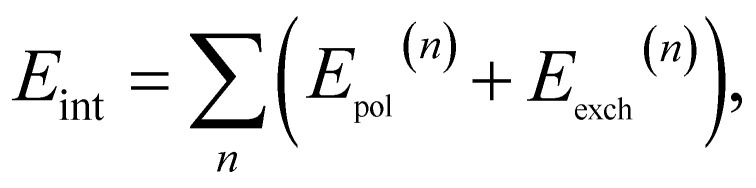
where *E*_pol_^(*n*)^ and *E*_exch_^(*n*)^ are nth-order polarization and exchange energies respectively.

In our previous work,^37^ we derived and implemented the first order polarisation energy, usually denoted as the electrostatic interaction energy *E*^(1)^_elst_ and the first order exchange energy *E*^(1)^_exch_.

In the following, we provide a brief overview over the second order terms (the detailed equations are presented in ESI Section 1).† For the second order polarisation energy, it is conventional in SAPT to split it into induction and dispersion energy, *i.e.*, *E*^(2)^_pol_ = *E*^(2)^_ind_ + *E*^(2)^_disp_.

The second order induction energy is *E*^(2)^_ind_ defined as:

4 *E*^(2)^_ind_ = *E*_ind_(A ← B) + *E*_ind_(B ← A)

where *E*_ind_(A ← B) is the induction of monomer A in the presence of monomer B and defined as:

5 *E*^(2)^_ind_(A ← B) = 〈*Ψ*^0^_A_*Ψ*^0^_B_|*V̂*|*Ψ*^ind^_A_*Ψ*^0^_B_〉

and the first order induction wave function for monomer A|*Ψ*^ind^_A_〉 is defined as:6
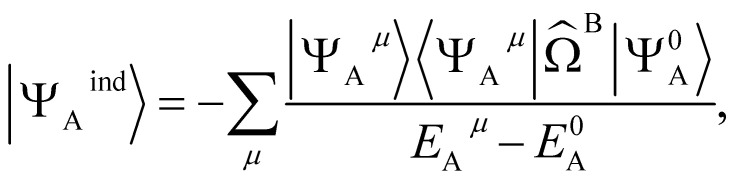
where |*Ψ*_A_^*μ*^〉 corresponds to the *μ*th excited state wavefunction and *E*_A_^*μ*^ to the *μ*th excited state energy of monomer A and *Ω̂*^B^ is the effective electrostatic potential of monomer B (see ESI 2† for an exact definition). It follows that7
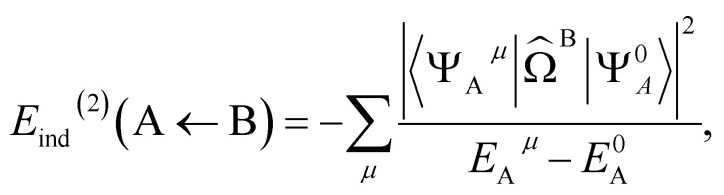
and *E*_ind_(B ← A) follows analogously.

The dispersion energy is defined as:

8 *E*^(2)^_disp_ = 〈*Ψ*^0^_A_*Ψ*^0^_B_|*V̂*|*Ψ*^disp^_AB_〉

where the first order dispersion wave function is defined as:9
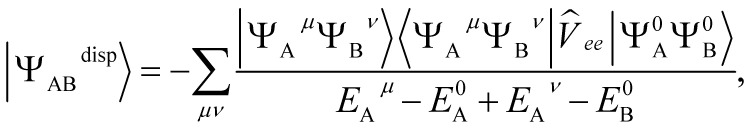
yielding:10
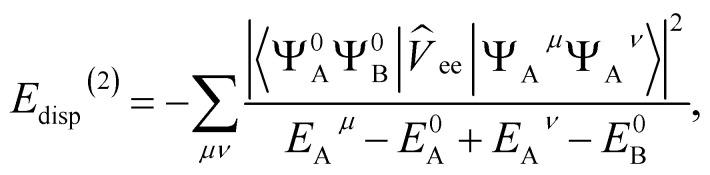
where *V̂*_ee_ is the usual electron–electron Coulomb operator.

If one neglects all electron exchange processes other than those that exchange a single electron between monomers A and B one arrives at the so-called *S*^2^ approximation to the exchange energies,^51–54^ and the second order exchange terms are defined as:

11 *E*^(2)^_exch-ind_(A → B) = 〈*Ψ*^0^_A_*Ψ*^0^_B_|(*V̂* − *V̄*)(*P̂* − *P̄*)|*Ψ*^ind^_A_*Ψ*^0^_B_〉

12 *E*^(2)^_exch-disp_ = 〈*Ψ*^0^_A_*Ψ*^0^_B_|(*V̂* − *V̄*)(*P̂* − *P̄*)|*Ψ*^disp^_AB_〉

where *P̂* is defined as the single electron exchange operator, *P̄* is defined as 〈*Ψ*^0^_A_*Ψ*^0^_B_|*P̂*|*Ψ*^0^_A_*Ψ*^0^_B_〉 and *V̄* is defined as *E*^(1)^_pol_.

The combination of both first and second order terms yields the following expression for the interaction energy:

13 *E*_int_ ≈ *E*_SAPT_ = *E*^(1)^_elst_ + *E*^(1)^_exch_ + *E*^(2)^_ind_ + *E*^(2)^_disp_ + *E*^(2)^_exch-disp_ + *E*^(2)^_exch-ind_

Both the dispersion and induction energies are often combined with their exchange counterparts and the order superscripts are dropped to yield a simplified energy expression:

14 *E*_SAPT_ = *E*_elst_ + *E*_exch_ + *E*_ind_ + *E*_disp_

(for further details on the derivations see ESI Section 1).†

If we use high quality monomer wavefunctions approaching full configuration interaction (FCI), we conceptually approach the SAPT(FCI) method of Korona and co-workers. In Korona's original work, the computation of the second-order induction and dispersion terms is performed by a direct response property treatment of static (induction) and frequency dependent dynamic susceptibility tensors (dispersion), followed by contractions of these property tensors to form polarization and exchange SAPT contributions.^47,52,55–57^ In this work, we instead employ extended random phase approximation (ERPA)^58^ as pioneered within SAPT(CASSCF) by Hapka^48,53,54,59^ to avoid computing excited state properties on a quantum computer as they often require a significant measurement overhead on NISQ-era quantum computers.^22,60–62^ We note that the use of ERPA as a proxy for the explicit response properties of the VQE resembles the quantum subspace expansion (QSE) method,^50^ wherein a basis of single and double excitations out of a VQE reference is used to provide an excited state ansatz that is truncated in character (but not in excitation energy).

Further details of SAPT and the ERPA procedure are provided in the ESI† along with explicit expressions for each term in terms of ERPA transition density matrices (ESI Sections 2 and 3).† We note that SAPT interaction energies using this second-order truncation of SAPT are typically highly accurate, even with Hartree–Fock wavefunctions (for the case of single-reference systems), and well established to produce accurate interaction energies in many common cases.^46^ Third- and higher-order extensions are likely possible along similar response properties or ERPA lines as used here, but are typically found to not improve the SAPT interaction energy significantly past the second-order level.

The current NISQ-era hardware is limited to tens of qubits (spin-orbitals); therefore an active space formalism is necessary to describe realistic chemical systems. In the active space approach, we partition the one-electron orbital set into core orbitals, active orbitals and virtual orbitals. Ideally, the active orbitals contain the orbitals required to describe the entangled electrons properly. The active space of the wave function is then calculated on a quantum computer (see ESI Section 1.2† for more information). In the SAPT(VQE) approach, one or both of the monomer active space wavefunctions are generated by VQE-type quantum circuits:

15 |*Ψ*_VQE_〉 ≡ *Û*_VQE_|*Φ*_I_〉

where |*Φ*_I_〉 is some initial state (typically the Hartree–Fock determinant). From these wavefunctions we obtain single-particle and two-particle reduced density matrices that go into the computation of the SAPT interaction energy. In this work we use a modified version of the unitary cluster Jastrow wavefunction ansatz^63^ (VQE) which takes the form16
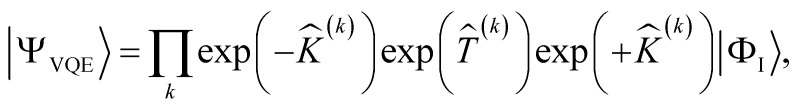
where *K̂*^(*k*)^ and *T̂*(*k*) are one- and two-body operators, and *k* is a parameter that controls the depth of the circuit and as a result its variational freedom. We use a slightly modified *k*-uCJ ansatz from ref. 63, which we denote as *k*-muCJ for clarity, with the ‘m’ standing for modified (see ESI Section 1.3† for more details). The SAPT(VQE) workflow is outlined at a high level in Fig. 1. We note that the SAPT(VQE) method as formulated within the ERPA picture is independent of the quantum algorithm used to determine the density matrices and thus can likely be readily adopted to any quantum algorithm of choice.

## 3. Results

**Fig. 2:**
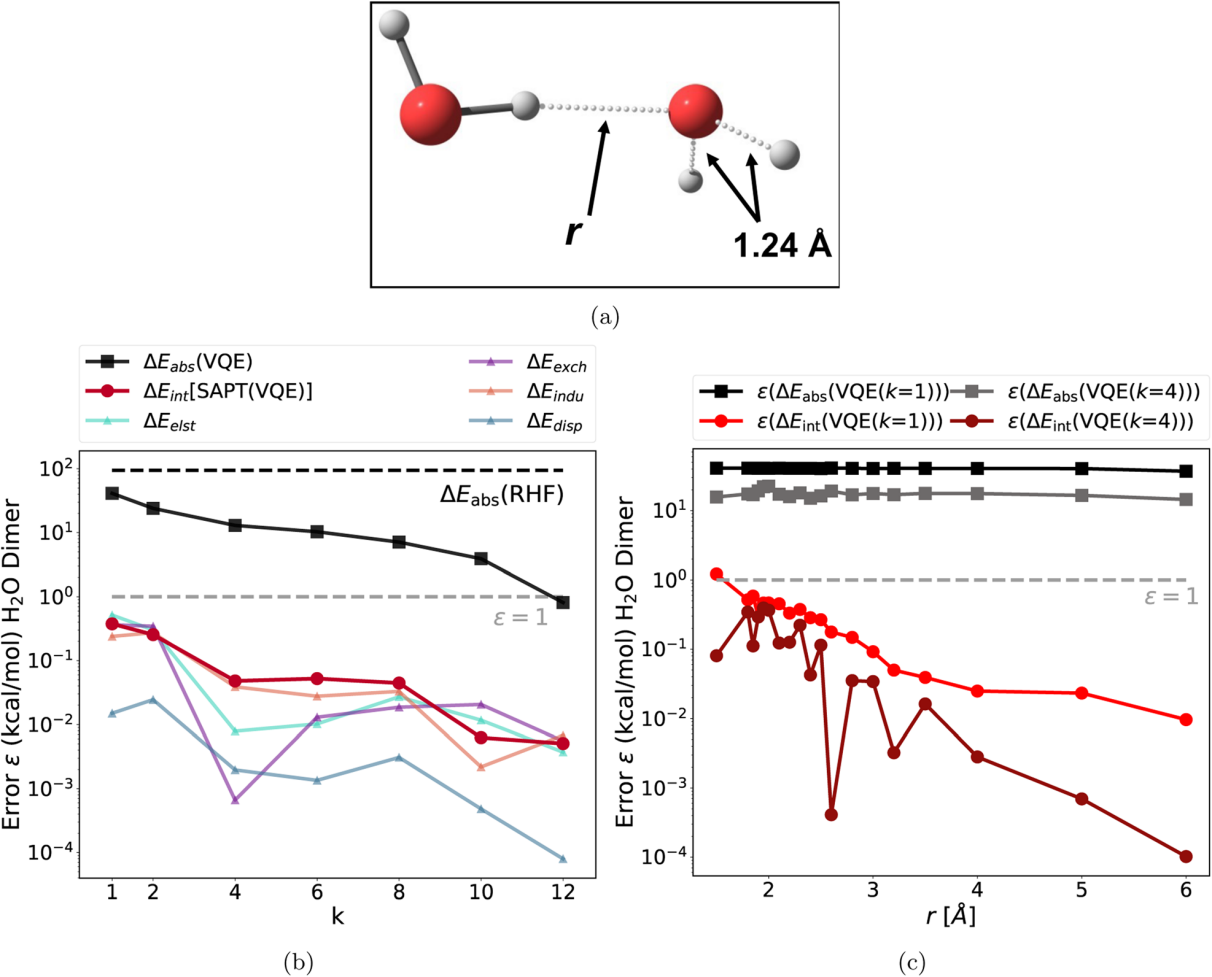
(a) Structure of the stretched water dimer; (b) absolute errors of the VQE total energy (stretched monomer) and each SAPT(VQE) energy term as a function of the repetition factor *k* at *r* = 2.0 Å. This shows that accurate interaction energies can already be obtained with coarsely optimized, low circuit depth wavefunctions from the quantum computer (errors relative to the CAS-CI and SAPT(CAS-CI) energies, and the dotted gray line represents the chemical accuracy 1 kcal mol^−1^ threshold); (c) absolute error of the VQE total energy (stretched water monomer) and each SAPT(VQE) (*k* = 1) and SAPT(VQE) (*k* = 4) energy term as a function of the intermolecular distance *r*. This shows that this empirical finding also holds for non-equilibrium bond distances.

**Fig. 3:**
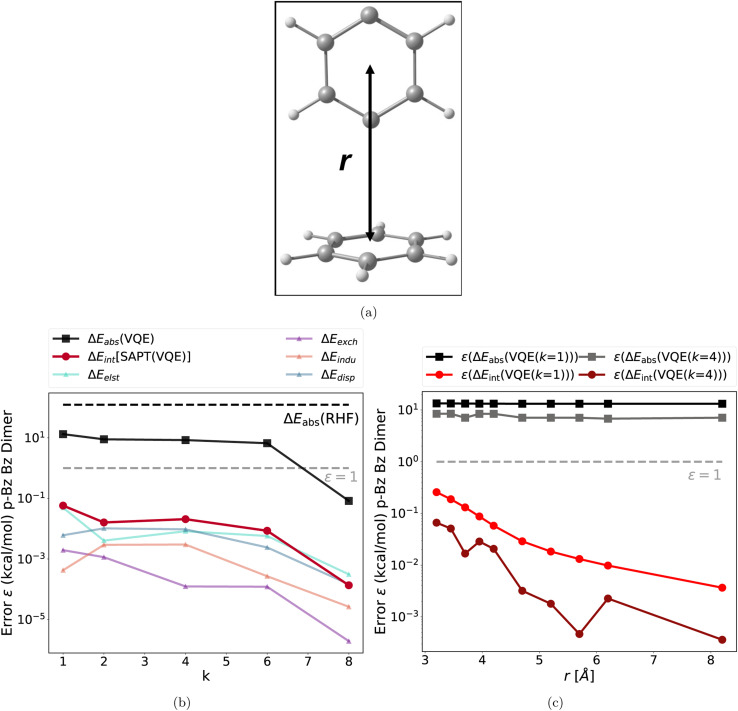
(a) Structure of the benzene *p*-benzyne dimer; (b) absolute errors of the VQE total energy (*p*-benzyne monomer) and each SAPT(VQE) energy term as a function of the repetition factor *k* at *r* = 3.9 Å (error relative to the CAS-CI and SAPT(CAS-CI) energies, and the dotted gray line represents the chemical accuracy 1 kcal mol^−1^ threshold); (c) absolute error of the VQE total energy (*p*-benzyne monomer) and each SAPT(VQE) (*k* = 1) and SAPT(VQE) (*k* = 4) energy term as a function of the intermolecular distance r. This shows that our empirical finding about accurate interaction energies also holds for different interaction motifs and different strongly correlated electronic structures.

**Fig. 4:**
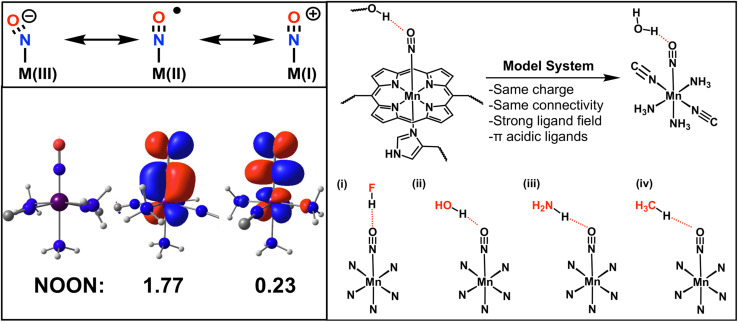
Upper left panel: different oxidation states of a transition metal bound NO; bottom left panel: structure of the [Mn(CN)_2_(NH_3_)_3_NO]^0^ complex and the natural orbitals (NOON) with the largest deviation from integer value occupation (π type metal to ligand backbonding); right panel: simplification of the heme ligand framework and schematic representation of the four manganese nitrosyl hydrogen bond complexes.

**Fig. 5:**
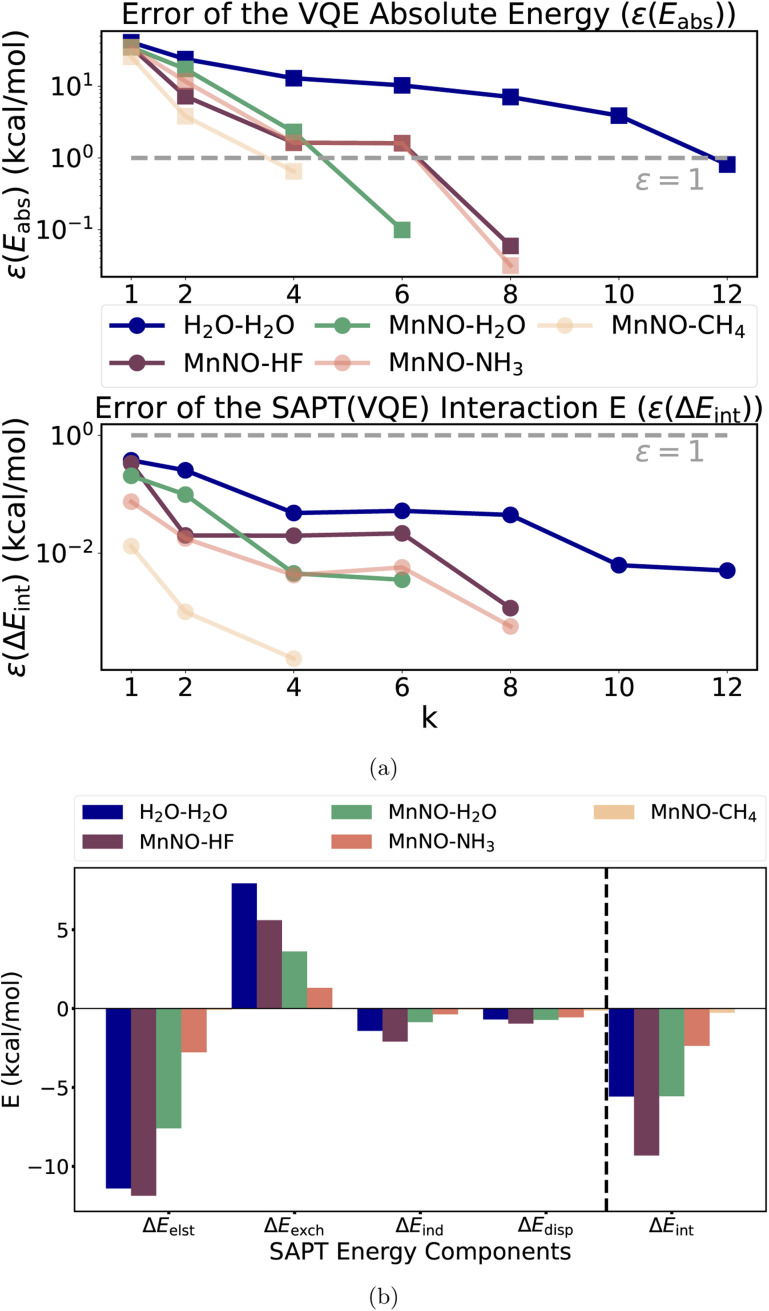
(a) Top figure: absolute errors of the VQE total energy for the [Mn(CN)_2_(NH_3_)_3_NO]^0^ complex and the “stretched” water; bottom figure: the SAPT(VQE) interaction energy as a function of the repetition factor *k* for the water dimer and each model heme-nitrosyl hydrogen bond complex showing that our empirical finding about accurate interaction energies also holds for a wide range of interaction energies (error relative to the CAS-CI and SAPT(CAS-CI) energies, and the dotted gray line represents the 1 kcal mol^−1^ threshold); (b) term-by-term decomposition of the SAPT(VQE) (*k* = 4) interaction energies of each model heme-nitrosyl hydrogen complex (see Table S1† for details).

As a first step, we test SAPT(VQE) with two classic intermolecular interaction motifs (water dimer and t-shaped benzene dimer). As a second step, we apply SAPT(VQE) to a heme-nitrosyl model complex; these systems are highly relevant in both biological^64^ and pharmaceutical^65^ chemistry. The SAPT(VQE) results presented in this section are the result of ideal statevector VQE simulations (see ESI Section 4† for more details). In all examples, we benchmark the accuracy of the VQE/SAPT(VQE) results by comparing to classical SAPT(CAS-CI) energies using the same orbitals and active space (see Fig. 2–5). The complete active space configuration interaction (CAS-CI) wavefunction represents the exact wavefunction within the active space approximation and thus, SAPT(CAS-CI) results represent the best possible interaction energy within the SAPT approximation but not the exact interaction energy (see Fig. S6† for a comparison of VQE and CAS-CI wave functions). We note that this comparison is only possible for small active space sizes due to the combinatorial scaling of the CAS-CI wavefunction ansatz.

### 3.1 Multi-reference benchmark systems

The chemistry of non-covalent interactions governs a wide range of interaction motifs such as hydrogen bonds or dispersion bound systems. However, the electronic structure of these simple systems is often well described by classical single reference methods. Therefore, we modified two of the classic systems in our previous study,^37^ namely the water dimer and the t-shaped benzene dimer, to make the electronic structure strongly correlated and thus challenging to compute accurately for conventional single reference methods.

The first test case is a hydrogen bonding motif: the stretched water dimer complex, which is depicted in Fig. 2a. The two partially broken single bonds make this system strongly correlated and require a multi-reference treatment to accurately describe the electronic structure. We included all eight valence electrons of the stretched monomer and eight spatial orbitals (8e and 8o) in the active space (for a detailed procedure on how the active orbitals were selected for the CASSCF calculations see ESI Section 4).† The CASSCF natural orbital occupation numbers (NOON, see Fig. S2†) exhibit deviations from integer values, which is an indicator of strong correlation. Consequently, the single reference RHF method fails to describe this system as apparent from the large deviation of the absolute energy of the monomer (see Fig. 2b black dotted line). This system also provides a challenge for the quantum algorithm as the *k*-muCJ ansatz needs a repetition factor of *k* > 10 to converge the absolute energy to the stretched monomer below 1 kcal mol^−1^. This can lead to large resource requirements for the supermolecular ansatz: in a worst case scenario for the supermolecular ansatz, both monomers are treated with an active space which then increases the resource requirement for the dimer computation even further (assuming that the active spaces combine). This is illustrated for the water dimer, we chose the same (8e, 8o) active space for the “stretched” water and add a (4e, 4o) active space for the second water yielding a (12e, 12o) and 24 qubits for the dimer. The VQE calculation for the dimer system did not converge sufficiently even with *k* > 20 (*ε* ∼30 kcal mol^−1^). This illustrates the difficulties of using the supermolecular ansatz in the NISQ era.

In sharp contrast to this, the errors of the total interaction energy as well as the errors of each individual SAPT energy term are multiple orders of magnitude lower than the absolute VQE errors (see Fig. 2(b)). In fact, the very shallow *k* = 1 circuit is accurate enough to provide interaction energies in comparison to the SAPT(CAS-CI) results. In the next step, we probed the bond dissociation of the water complex (along the intermolecular O–H bond labeled *r* in Fig. 2a). We find a similar behavior: the errors in the interaction energies and each energy component are below 1 kcal mol^−1^ for all intermolecular distances and several orders of magnitude lower than the error in absolute energies. For *k* = 4, each interaction energy is below the 1 kcal mol^−1^ threshold; for *k* = 1 some errors are slightly larger at small intermolecular distances.

The second test case is a dispersion bound complex: the T-shaped benzene *p*-benzyne dimer, depicted in Fig. 3a. The *p*-benzyne monomer has a biradical ground state, which is difficult to describe with classical single reference methods.^66^ The *k*-muCJ VQE ansatz requires *k* = 8 for a moderate (6e, 6o) active space to reach sub 1 kcal mol^−1^ accuracy as illustrated in Fig. 3b. The key findings are identical to the previous test case and also hold for different intermolecular distances (see Fig. 3b and c). Thus, these findings hold for very different types of intermolecular interaction motifs, intermolecular distances, different active spaces and different type difficult electronic structures.

### 3.2 Hydrogen bonding to heme-nitrosyl model complexes

As an application example, we study hydrogen bonding to a manganese nitrosyl complex. Nitric oxide (NO) is a small molecule with important biological implications such as signal transduction^65,67^ or as a key intermediate in the nitrogen cycle.^64,68^ At the center of these processes are metalloporphyrins,^69–71^ where NO binds to the metal center as a nitrosyl ligand.^72,73^

In order to understand and control these biological processes, the chemistry around the metal–NO bonds must be elucidated in terms of electronic structure and reactivity^74^ as illustrated by theoretical,^75^ experimental^76^ and medicinal^77–79^ work. Unfortunately, the metal–NO bond in nitrosyl complexes poses a challenge for many quantum chemistry methods due to the redox active nature of the NO ligand.^80^ There are three possible oxidation states for the NO moiety: NO^−^, NO˙ and NO^+^, which is illustrated for a generic M–NO complex in Fig. 4. In many cases, the bond is best described in terms of a superposition of these states. This strong correlation makes this a challenging system for many single reference methods such as DFT.^81^ This results in a large variety of recommended functionals depending on the specific nitrosyl complex studied.^70,75,82,83^

In this work, we study the hydrogen bonding to a heme-model manganese–nitrosyl complex. This model can serve as a proxy of how a metal-heme bound NO interacts with a protein environment as depicted in Fig. 4. The metal-coordinating cyano and ammonia ligands resemble a porphyrin coordination environment in terms of the ligand field, total charge and π-acidity (see Fig. 4). As hydrogen bond donors we chose HF, H_2_O, NH_3_ and CH_4_, which cover a wide range of donor strengths similarly to active sites in proteins. The resulting four hydrogen complexes are depicted in Fig. 4 and are abbreviated as MnNO⋯HF, MnNO⋯HOH, MnNO⋯HNH_2_ and MnNO⋯HCH_3_.

As a first step, we analyzed the electronic structure of [Mn(CN)_2_(NH_3_)_3_NO]^0^ and found the system to be strongly correlated (see ESI Section 5† for a more detailed discussion). We used a (6e, 6o) active space for the subsequent CASSCF and VQE calculations to include all 6 electrons of the Mn–NO bond. The key six active orbitals are centered around the Mn–NO moiety and are very similar to the active orbitals in real heme nitrosyl complexes^84^ (see Fig. S2 and Section 5†). The *k*-uCJ VQE ansatz required up to *k* = 8 layers to converge to the CASSCF energy within 1 kcal mol^−1^ (the variations are caused by the additional ghost-basis functions from the different hydrogen bonding donors). In contrast, the SAPT interaction energy is already significantly below that threshold even for *k* = 1. It is noteworthy that this finding holds true for the wide range of interaction energies in this series (−0.3 to −9.3 kcal mol^−1^, see Fig. 5a). Thus, we confirm the core finding of this work for different interaction motifs, a wide range of interaction energies, different strongly correlated electronic structure systems and both equilibrium and non-equilibrium bond distances. However, we reiterate that this work uses an ideal statevector simulator; thus, the effect of noise is yet to be seen.

**Fig. 6:**
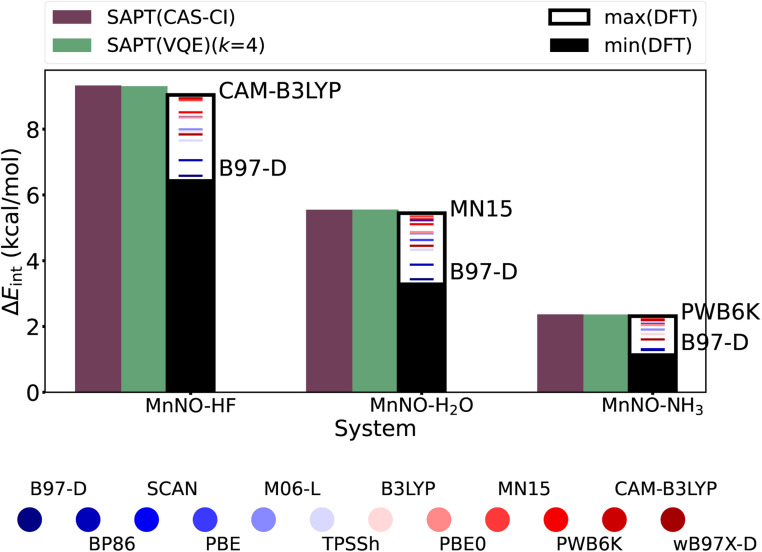
Interaction energies using several approximate DFT exchange correlation functionals (supermolecular approach) in comparison to the SAPT(CAS-CI) and SAPT(VQE) (*k* = 4) interaction energies showing the sensitivity of the results for a given hydrogen bonding complex–exchange correlation functional pair (see Table S3† for more details).

In addition to the interaction energy, SAPT provides a decomposition into physical meaningful terms helping to unravel the origin of the interaction. Fig. 6b plots each component of the SAPT(VQE) (*k* = 4) calculation of the series of hydrogen bonded complexes plus the stretched water dimer as a reference of a typical hydrogen bond (see ESI Section 5.1, Table S1 and Fig. S7† for more details including bond distances). The main driving force for binding is the electrostatic term as expected for hydrogen bonds. The strongest contrast between the water dimer and the Mn–NO hydrogen bonds is observed in the exchange energy and is the main driving force for the difference in interaction energies. This may be rationalized by the difference in the diffuseness of the lone pairs: the bound NO becomes (partly) NO^+^, which makes the lone pair more compact in space than the lone pair in the water dimer, thus resulting in less exchange repulsion.

Finally, we compare the SAPT(VQE) interaction energy to DFT based supermolecular (BSSE corrected^85^) interaction energies, the standard approach on classical hardware. The exact comparison is difficult as there is no standard procedure to obtain accurate interaction energies for strongly correlated systems. In addition, we use a small basis set [due to technical limits in the current CuPy classical implementation of SAPT(VQE)]. However, nitrosyl complexes are an example of the non-universality problem of approximate density functionals as the hydrogen bonding moiety and the nitrosyl moiety prefer different approximate density functionals^75,84,86^ and thus reliable predictions are only possible with careful system specific benchmarking when experimental data are available.^70^ In contrast, SAPT is expected to robustly give accurate results for hydrogen bonds given appropriate monomer wavefunctions, *e.g. via* a quantum algorithm in SAPT(VQE). To illustrate this point, Fig. 6 plots the interaction energies of SAPT(VQE), SAPT(CAS-CI) and several popular DFT functionals (through the supermolecular approach). We included many popular functionals as well as several top performing functionals for non-covalent interactions.^87^ We see in Fig. 6 that the SAPT(VQE) (*k* = 4) is almost identical to the SAPT(CAS-CI) in all four cases. The DFT functionals exhibit a significant spread for each complex. The B97-D functional predicted the smallest interaction energy in all four cases, but the highest interaction energy is predicted in each case by a different functional. Furthermore, we see the relative ordering of the functional change for each system (color sequence in each plot). This illustrates the non-universality problem for approximate exchange correlation functionals even for very similar nitrosyl complexes (this also holds true for a larger basis set as illustrated in Fig. S8†). Note that the SAPT(CAS-CI) results are the reference for the SAPT(VQE) calculations and do not represent the true interaction energy, and thus, only the SAPT(VQE) and not the DFT interaction energies should be compared against this reference.

To demonstrate that the erroneous behavior of DFT is related to the system studied here, we calculate azanone hydrogen complexes with HF and H_2_O (see Fig. S9†). These hydrogen complexes are the main group analogues of the nitrosyl complex where we replace the Mn–NO with a H–NO bond. This results in a much simpler electronic structure without strong correlation where we can generate reference energies using coupled cluster wave function methods. We find that many DFT functionals perform within 1 kcal mol^−1^ accuracy. Interestingly, we see that the relative ordering of the functional changes notably from the MnNO to the HNO systems. Furthermore, we note that SAPT predicts the interaction energies with <0.5 kcal mol^−1^ error in both cases using the optimal basis set (see Fig. S9(a) and (b); see ESI Section 5.2† for details on the reference energies). We can expect SAPT(VQE) ansätze to yield similarly accurate results for strongly correlated examples as presented above when the optimal basis is used. Therefore, the SAPT formalism, presented in this work, is able to provide accurate interaction energies both for simple and difficult electronic structures, while the accuracy of DFT deteriorates for the latter.

## 4. Conclusion

With the development of the present manuscript, we have what we believe represents a minimally complete path for the accurate determination of intermolecular interaction energies on a NISQ-type computer. Our previous study^37^ established the theoretical framework of SAPT(VQE) but was limited to only first order terms of electrostatics and exchange for a proof-of-concept demonstration. This work obviates this limitation by including the second order terms of induction and dispersion, including their exchange counterparts. This results in a level of SAPT that is expected to produce accurate interaction energies with or near chemical accuracy.^46^ In this hybrid quantum-classical procedure, we obtain monomer wavefunctions on a quantum computer *via* the VQE algorithm and measure the one- and two-particle reduced density matrices of the monomers (simulated through ideal statevectors in the present work). On a classical computer we compute the first and second order SAPT contributions based on the reduced density matrices from VQE calculation. The direct computation of excited states for the second order terms is avoided *via* an extended random phase approximation (ERPA) formalism.

We find empirically that SAPT(VQE) can provide accurate interaction energies even with coarsely optimized, low circuit depth wavefunctions from the quantum computer. The resulting errors of first and second order contributions, in addition to the total interaction energies, are orders of magnitude lower than the corresponding VQE total energies of the monomer wavefunctions. Our empirical findings are based on the application of the SAPT(VQE) method to several systems with strongly correlated electronic structures: two classic intermolecular interaction motifs and several hydrogen bonding complexes of a heme-nitrosyl model complex, a class of biological highly relevant metalloenzymes where classic quantum chemistry methods such as DFT struggle to obtain accurate interaction energies. Thus, this works paves the way to obtain accurate interaction energies on a NISQ-era quantum computer with few quantum resources. It is the first step in alleviating one of the major challenges in quantum chemistry where in-depth knowledge of both the method and system is required *a* priori to reliably generate accurate interaction energies.

While a basic path to NISQ-type computations of interaction energies is now reasonably clear, much remains to improve the details of the operational concept. One basic direction that needs improvement is the classical acceleration of the ERPA and SAPT terms – our naïve CuPy code for this consideration was severely limited in system size due to a non-optimal treatment of core/active/virtual simplification and a lack of density fitting of the response functions.^57^ The implementation of the latter would alleviate the limitation to small basis sets, as medium size basis sets are required for accurate interaction energies with second order SAPT methods. In another instance, it may be that the ERPA formalism for the second order terms could be improved by direct treatment of the response of the monomers to external perturbations, including coupled response of the quantum and classical monomer wavefunction parameters. It might also be the case that more-advanced treatment of the required observables along the lines of double factorization^88,89^ could significantly reduce the number of required measurements when the time comes to deploy this method in the presence of shot noise. As the present work used ideal statevector simulators, the effect of noisy RDMs remains unclear. However, efforts are already underway to gauge this effect. Another highly interesting question is how the present approach might map (if at all) to the fault tolerant regime where statistical evaluation of expectation values is definitionally prohibitive. Finally, beyond the concept of interaction energies, this work represents our general hypothesis of the level of specialization needed to converge various important chemical observables - we believe that other properties such as gradients, polarizabilities, spectroscopies, *etc.,* may require similar quantum adaption of rather verbose classical methods like SAPT to provide good convergence of observables on NISQ devices.

## Data availability

Data for all application examples availe in the ESI.† In addition, XYZ structure and NPZ files for the MnNO⋯HF example. We also include python code to obtain and save the active space Hamiltonian *via* PySCF.

## Author contributions

All authors discussed and designed the formalism of an ERPA-based extension of SAPT(VQE) for induction and dispersion. F. D. M., A. R. W., and R. M. P. carried out the initial derivation of the equations. F. D. M. performed extensive simplifications and spin-adaption of the equations. F. D. M. wrote the CuPy implementation of the method. M. L., T. F., M. D, and N. M. prepared the geometries and performed the classical reference computations. M. L. and F. D. M. performed the simulated SAPT(VQE) computations. M. L. wrote the initial draft of the manuscript. All authors assisted with the production and analysis of the application results and with the writing of the manuscript.

## Conflicts of interest

M. L., A. R. W. and R. M. P. own stock/options in QC Ware Corp.

## Supplementary Material

SC-014-D2SC05896K-s001

SC-014-D2SC05896K-s002

SC-014-D2SC05896K-s003
