# Supplementary material for: Accurate non-covalent interaction energies on noisy intermediate-scale quantum computers via second-order symmetry-adapted perturbation theory
Source: Chem Sci. 2023 Feb 23;14(13):3587–99. doi: 10.1039/d2sc05896k (PMC10055839; doi:10.1039/d2sc05896k)
Supplement: SC-014-D2SC05896K-s001 [file SC-014-D2SC05896K-s001.zip › Manuscript_tex/Figures/SAPT_workflow_color.pdf]

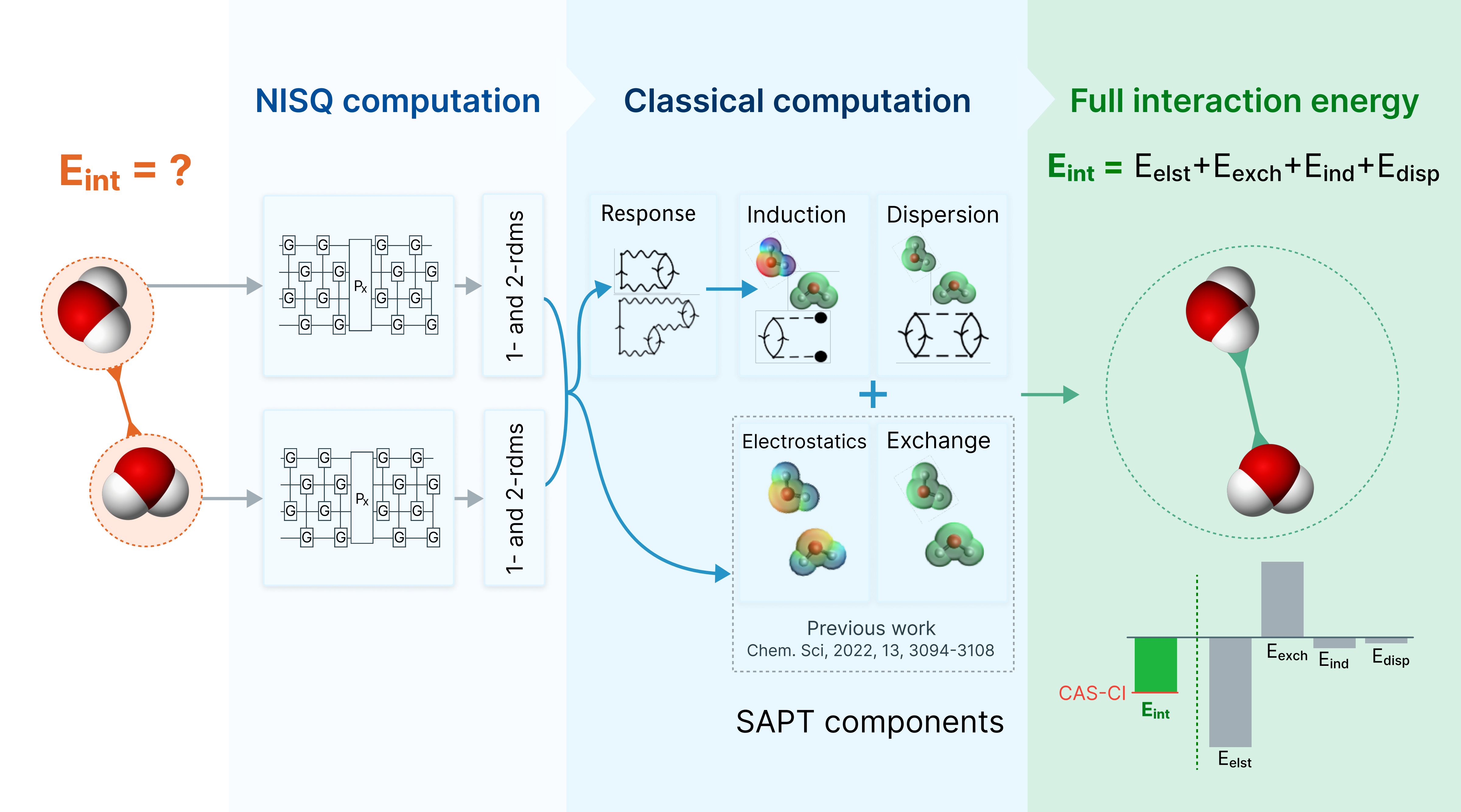

NISQ computation
Classical computation
Full interaction energy
E_int = ?
E_int = E_elst + E_exch + E_ind + E_disp
P_x
1- and 2-rdms
1- and 2-rdms
Response
Induction
Dispersion
+
Electrostatics
Exchange
Previous work
Chem. Sci, 2022, 13, 3094-3108
SAPT components
E_exch
E_ind
E_disp
CAS-CI
E_int
E_elst
