# Supplementary material for: Accurate non-covalent interaction energies on noisy intermediate-scale quantum computers via second-order symmetry-adapted perturbation theory
Source: Chem Sci. 2023 Feb 23;14(13):3587–99. doi: 10.1039/d2sc05896k (PMC10055839; doi:10.1039/d2sc05896k)
Supplement: SC-014-D2SC05896K-s001 [file SC-014-D2SC05896K-s001.zip › Manuscript_tex/Figures/Error_PES_H2O.pdf]

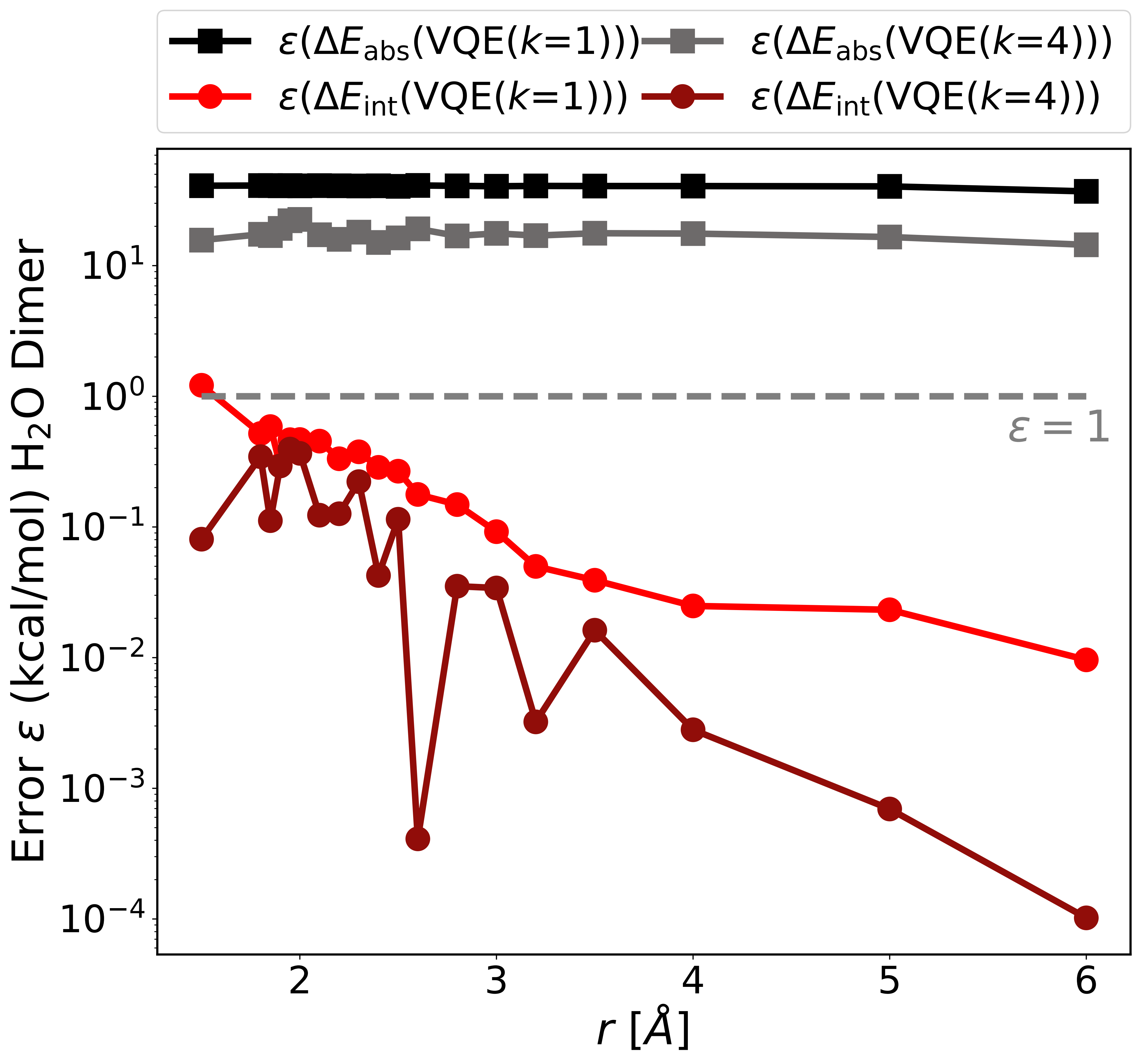

$\varepsilon(\Delta E_{abs}(VQE(k=1)))$
$\varepsilon(\Delta E_{abs}(VQE(k=4)))$
$\varepsilon(\Delta E_{int}(VQE(k=1)))$
$\varepsilon(\Delta E_{int}(VQE(k=4)))$
$\varepsilon = 1$
Error ε (kcal/mol) H_2O Dimer
10^{1}
10^{0}
10^{-1}
10^{-2}
10^{-3}
10^{-4}
2
3
4
5
6
r [Å]
