# Supplementary figures and images for: Accurate non-covalent interaction energies on noisy intermediate-scale quantum computers via second-order symmetry-adapted perturbation theory
Source: Chem Sci. 2023 Feb 23;14(13):3587–99. doi: 10.1039/d2sc05896k (PMC10055839; doi:10.1039/d2sc05896k)

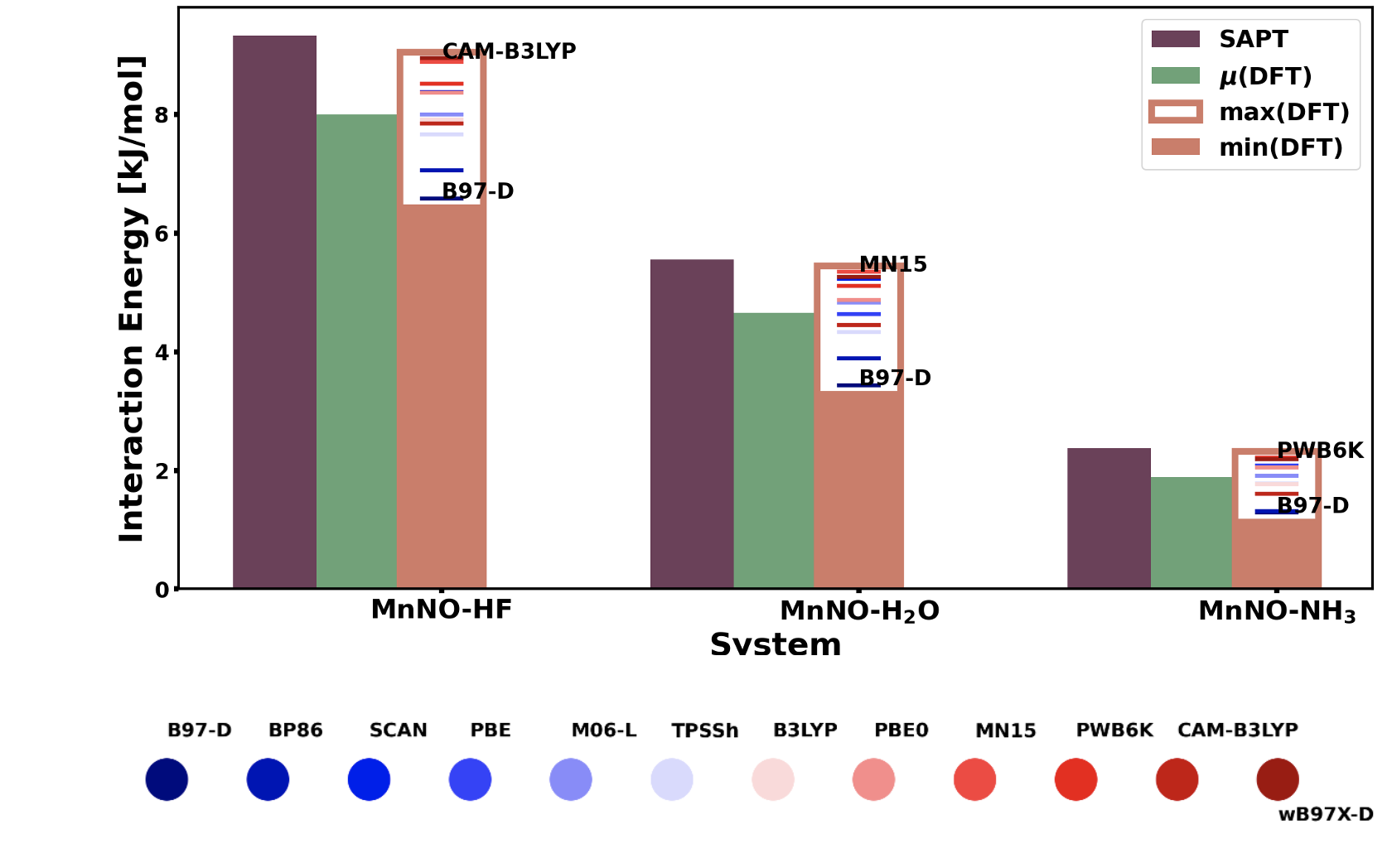

Supplement: SC-014-D2SC05896K-s001 [file SC-014-D2SC05896K-s001.zip › Manuscript_tex/Figures/MnNO_sapt_vs_dft.png]

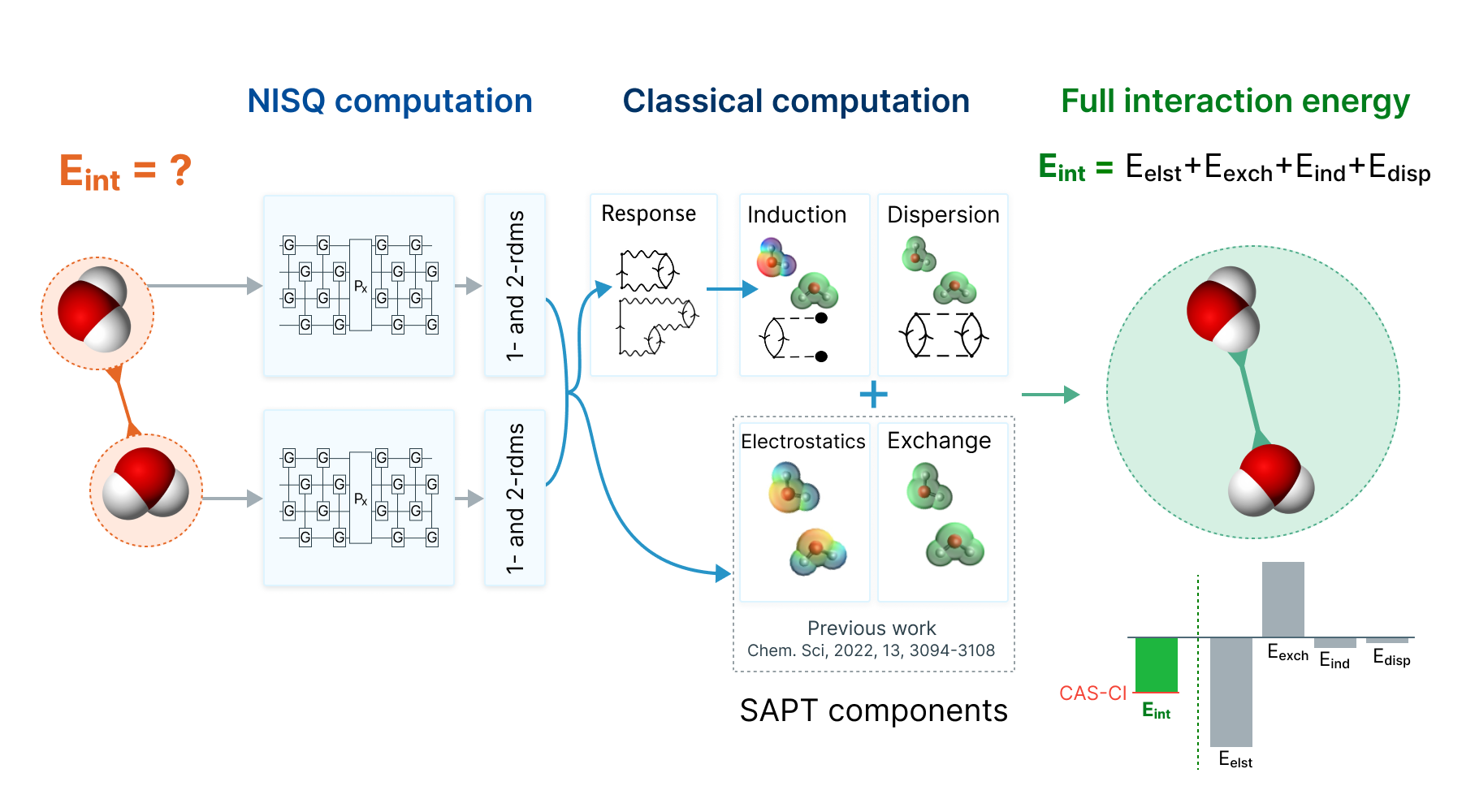

Supplement: SC-014-D2SC05896K-s001 [file SC-014-D2SC05896K-s001.zip › Manuscript_tex/Figures/SAPT_workflow.png]

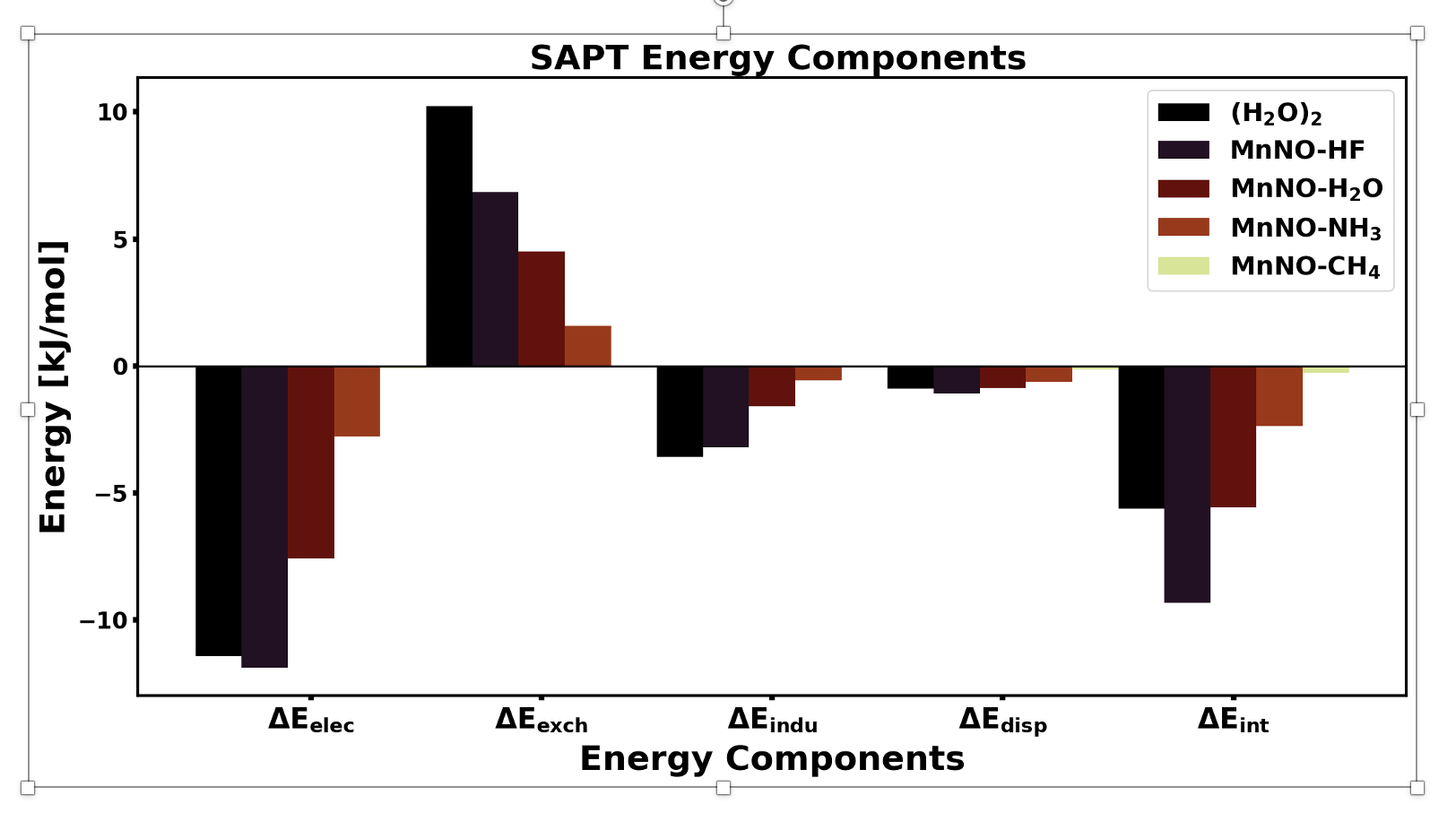

Supplement: SC-014-D2SC05896K-s001 [file SC-014-D2SC05896K-s001.zip › Manuscript_tex/Figures/MnNO_results.png]

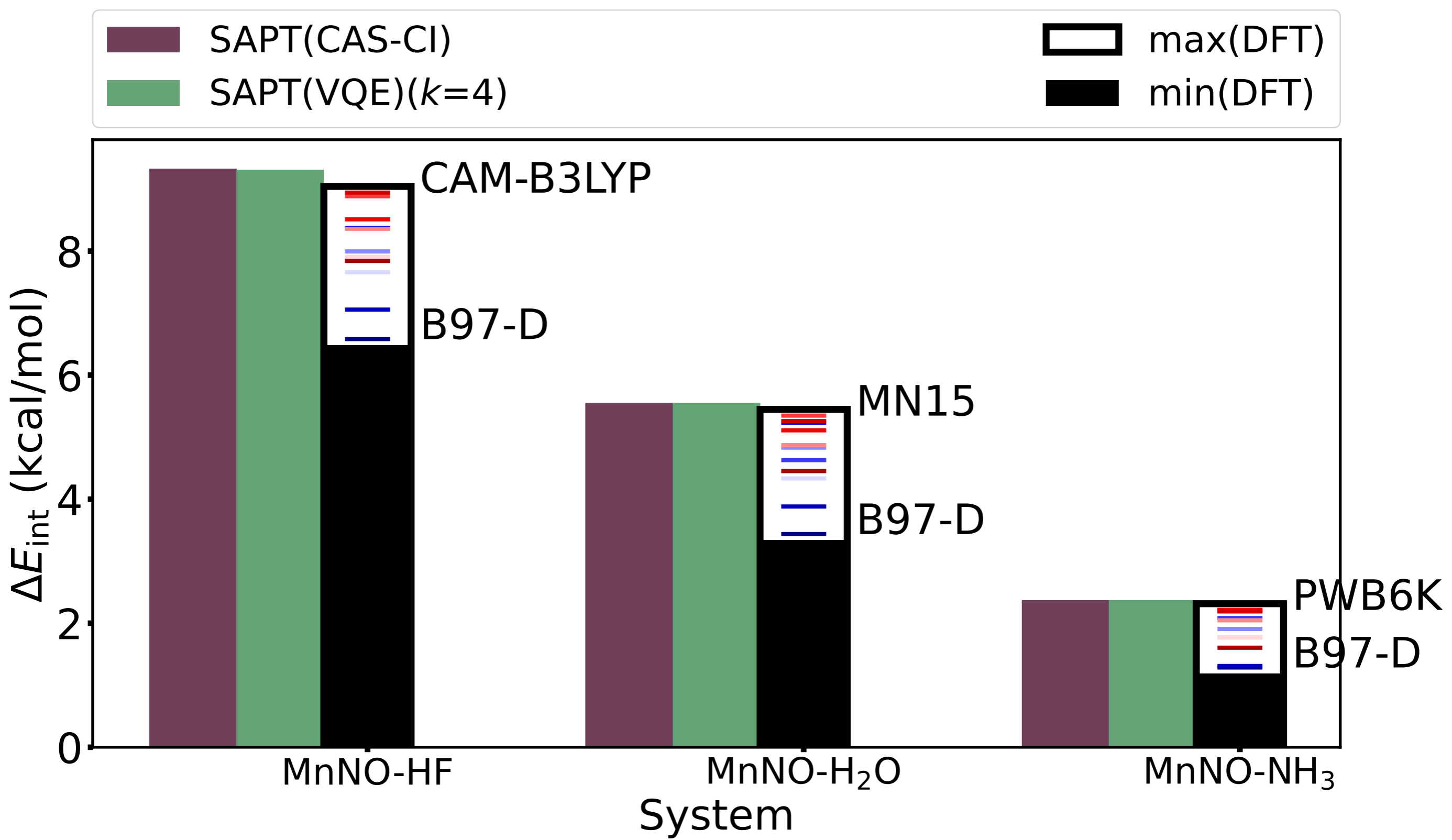

Supplement: SC-014-D2SC05896K-s001 [file SC-014-D2SC05896K-s001.zip › Manuscript_tex/Figures/DFT_SAPT_Mn.pdf]

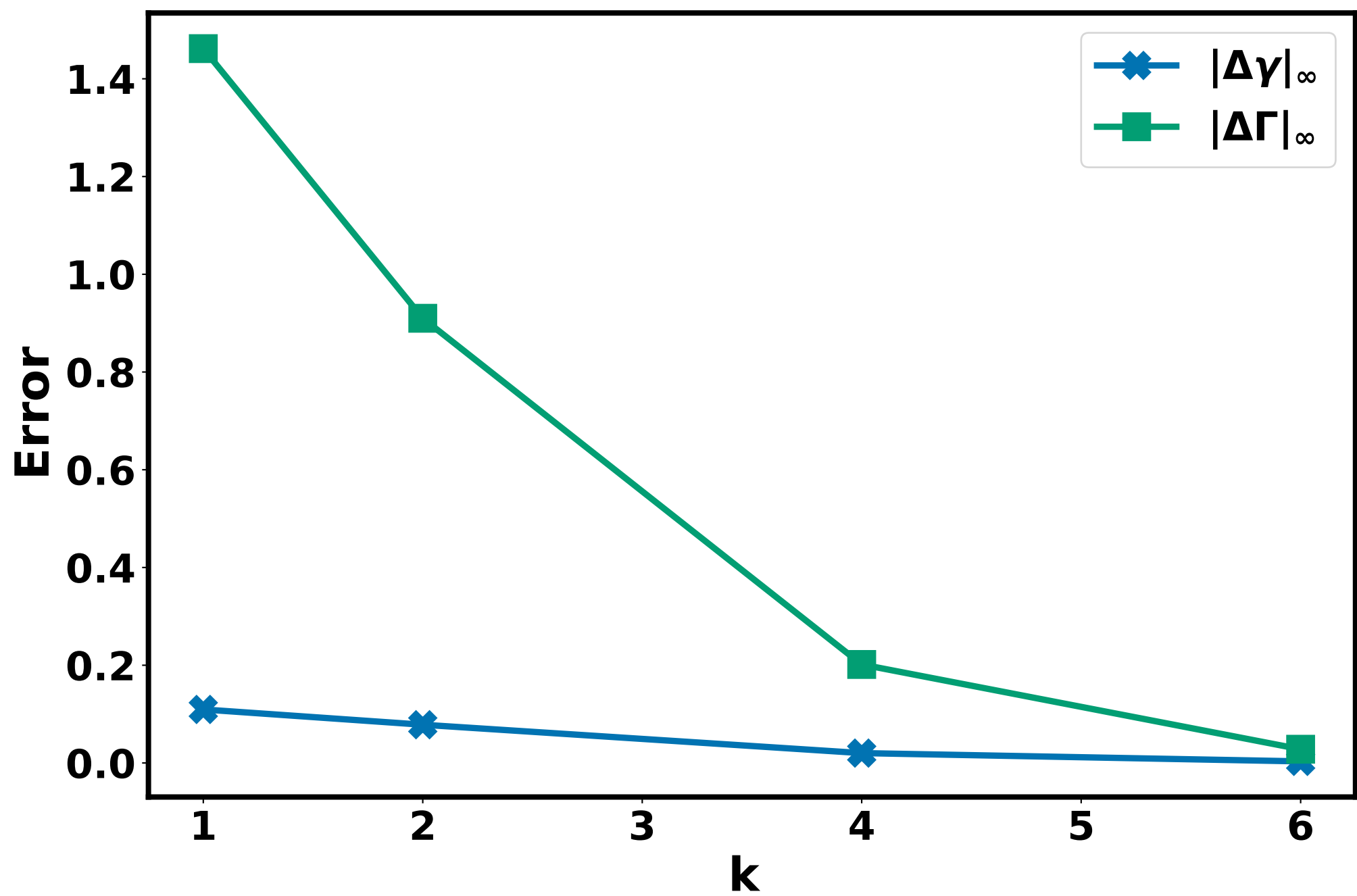

Supplement: SC-014-D2SC05896K-s001 [file SC-014-D2SC05896K-s001.zip › Manuscript_tex/Figures/pdm_error.pdf]

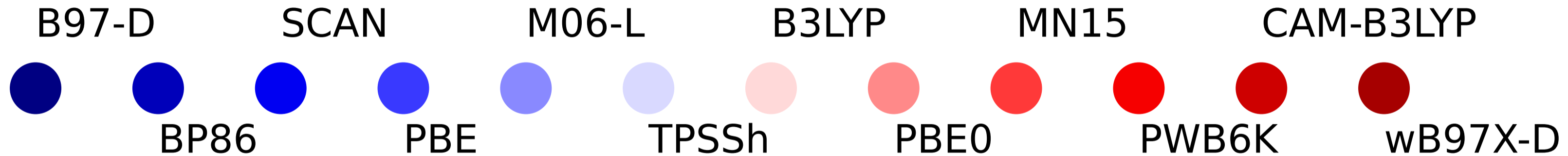

Supplement: SC-014-D2SC05896K-s001 [file SC-014-D2SC05896K-s001.zip › Manuscript_tex/Figures/DFT_color_labels.pdf]

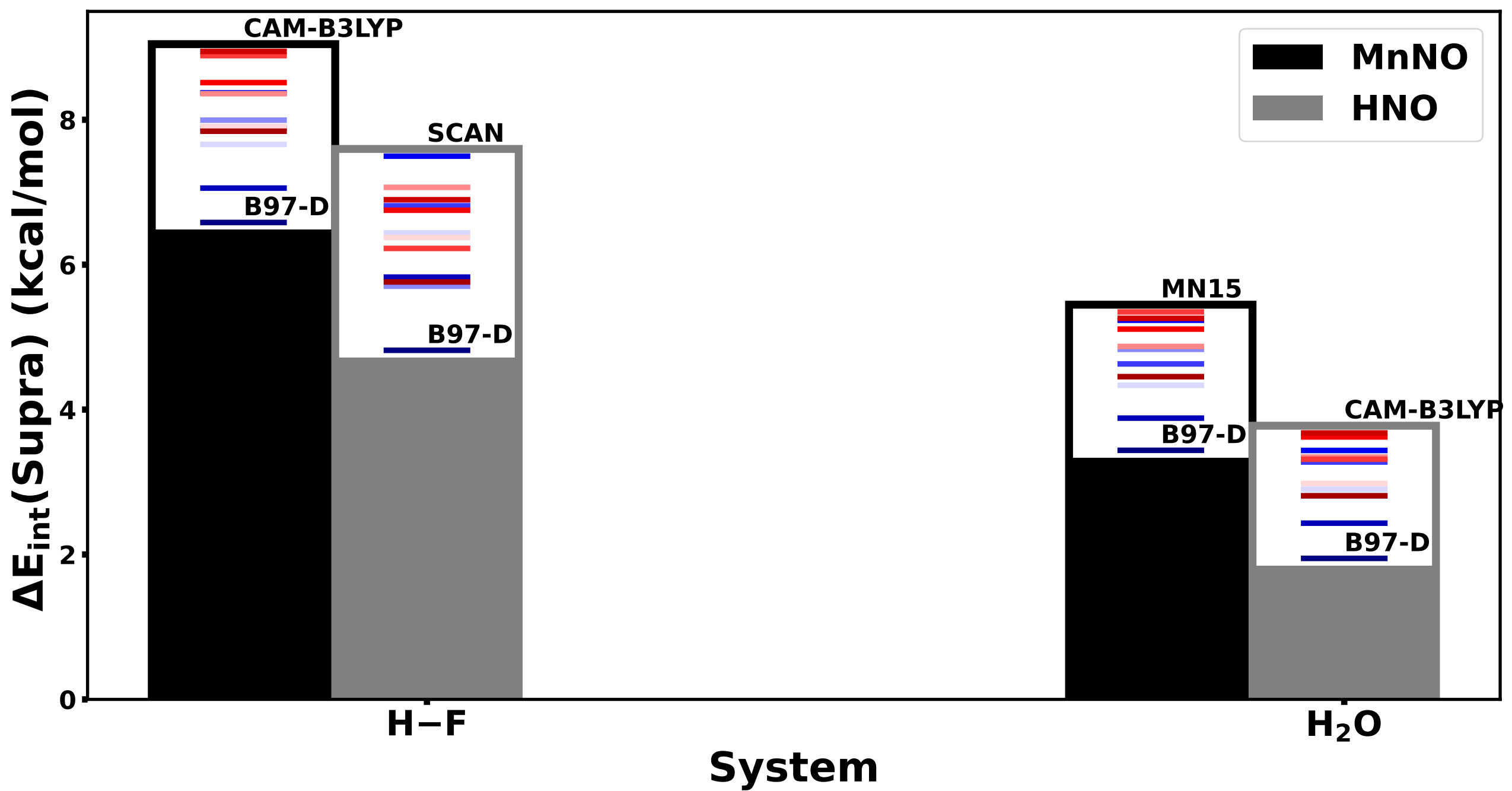

Supplement: SC-014-D2SC05896K-s001 [file SC-014-D2SC05896K-s001.zip › Manuscript_tex/Figures/SAPT_DFT_Mn_vs_HNO.pdf]

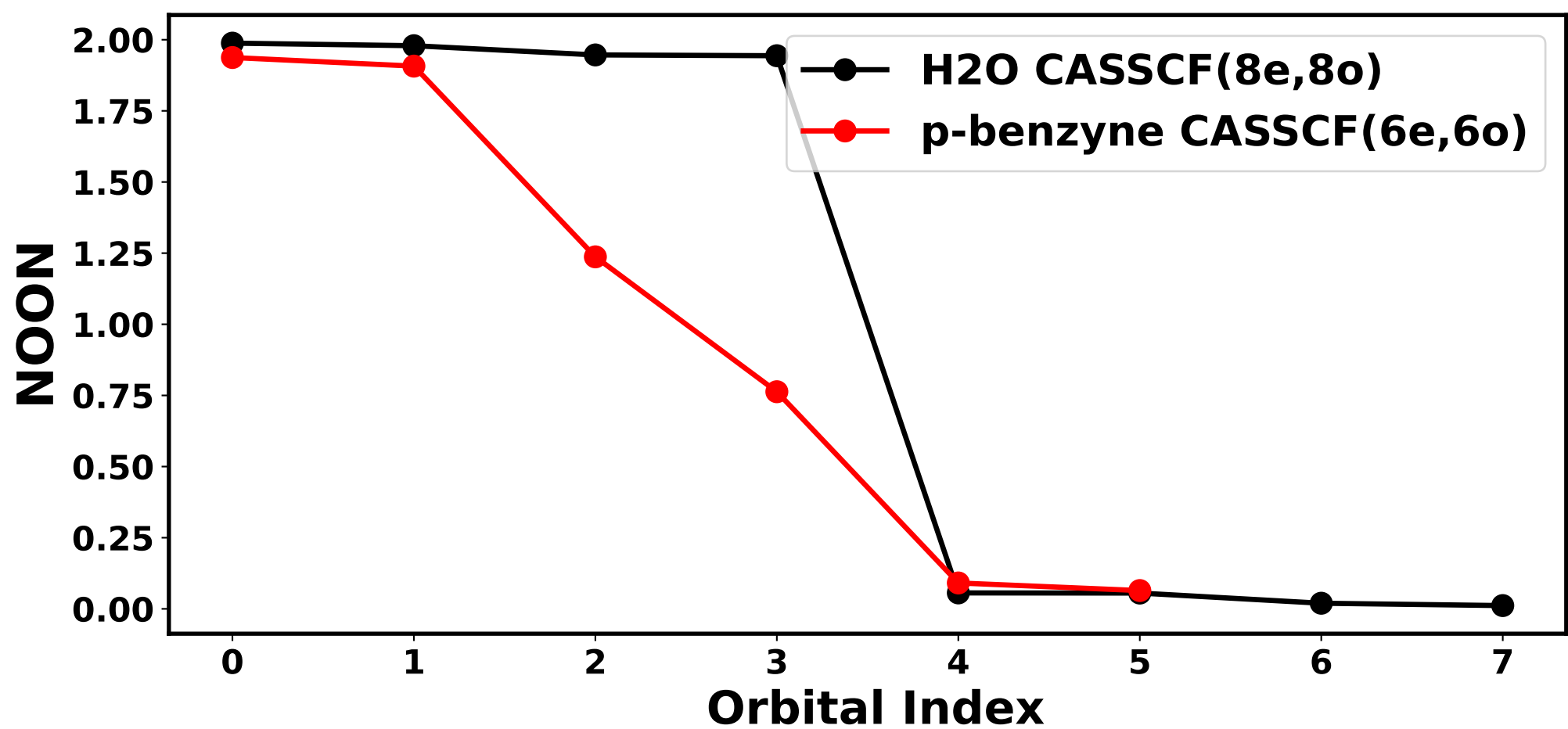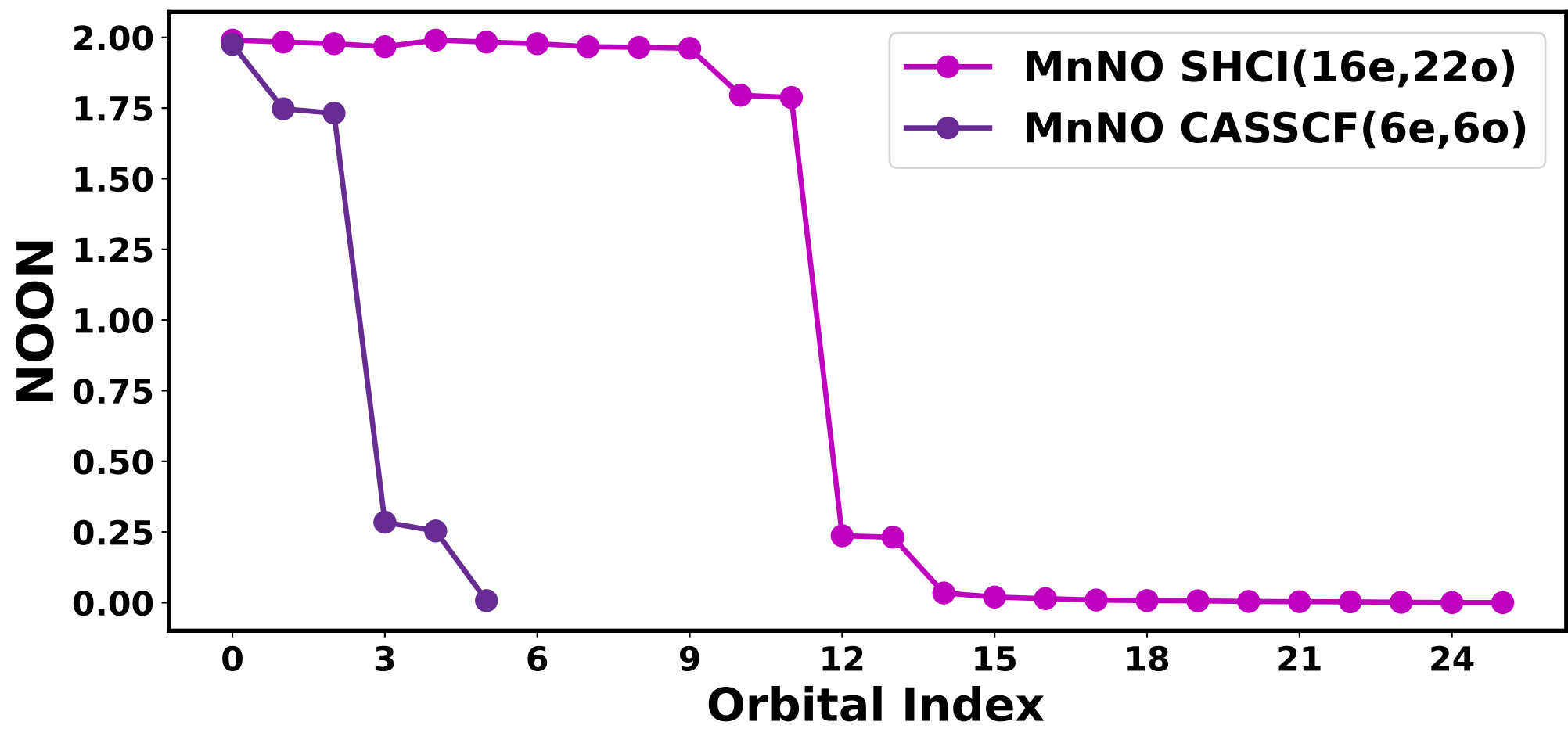

Supplement: SC-014-D2SC05896K-s001 [file SC-014-D2SC05896K-s001.zip › Manuscript_tex/Figures/NOON.pdf]

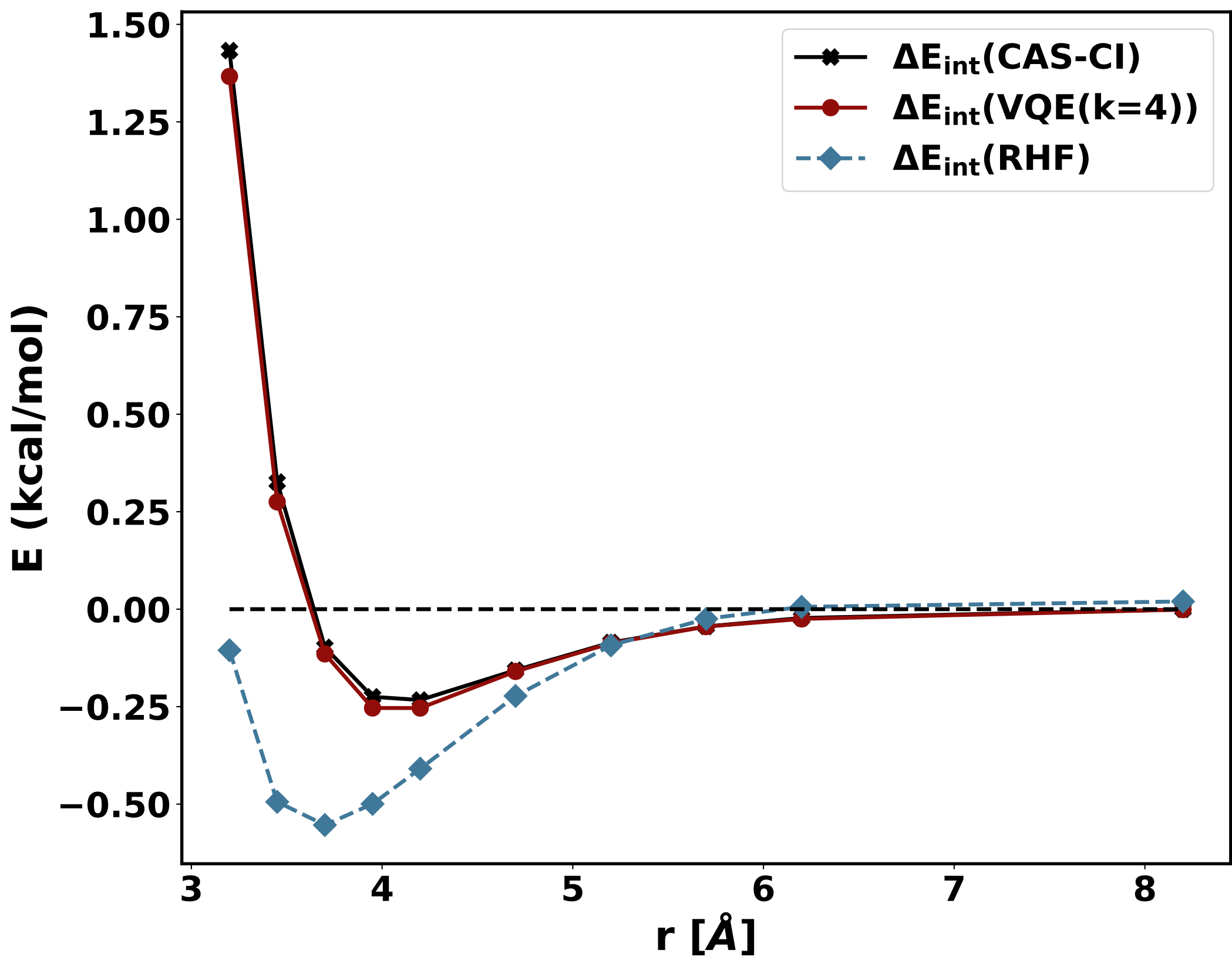

Supplement: SC-014-D2SC05896K-s001 [file SC-014-D2SC05896K-s001.zip › Manuscript_tex/Figures/PES_BZ.pdf]

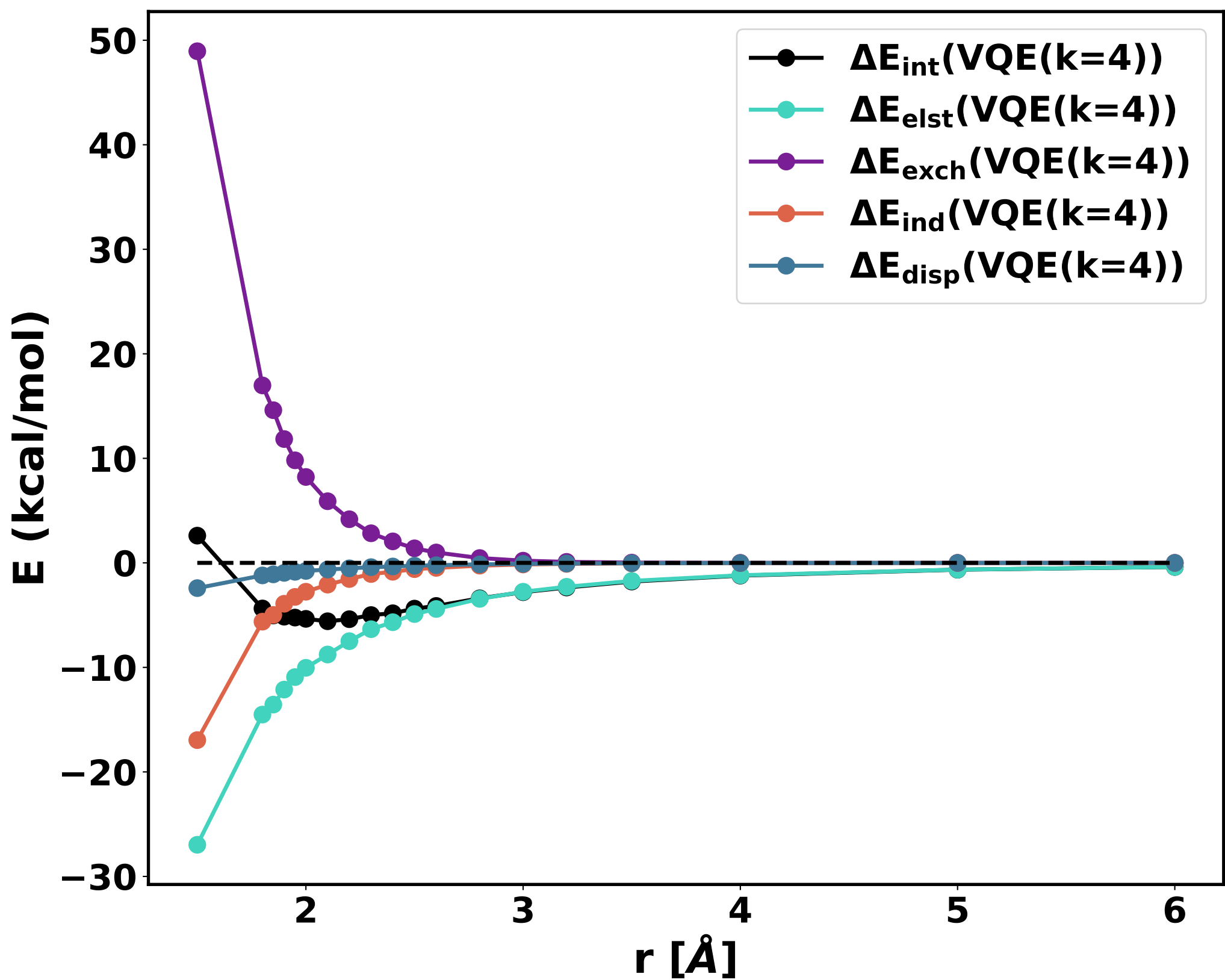

Supplement: SC-014-D2SC05896K-s001 [file SC-014-D2SC05896K-s001.zip › Manuscript_tex/Figures/PES_H2O_SAPT_Terms.pdf]

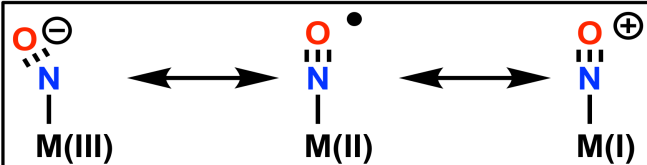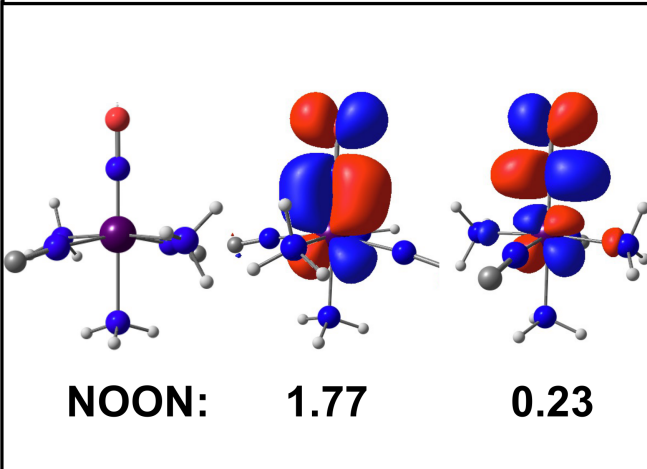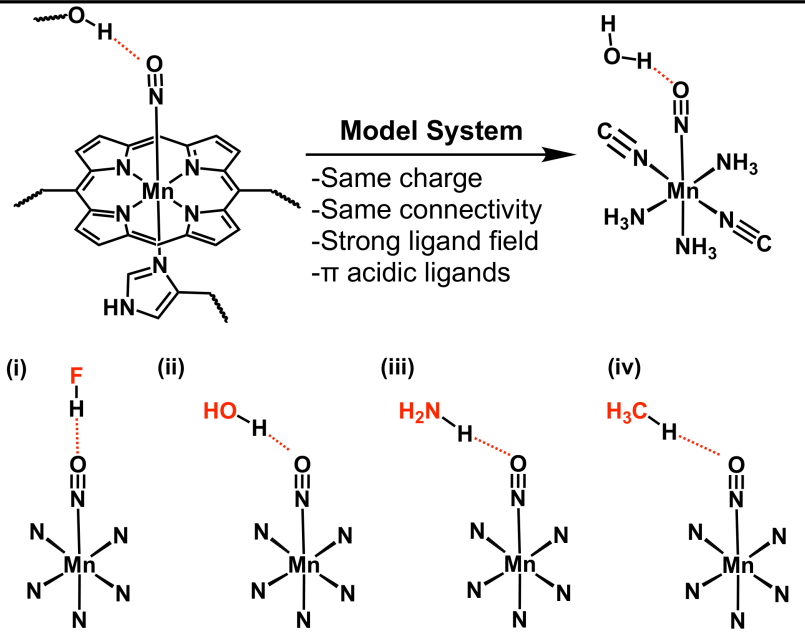

Supplement: SC-014-D2SC05896K-s001 [file SC-014-D2SC05896K-s001.zip › Manuscript_tex/Figures/Ligand_scheme.pdf]

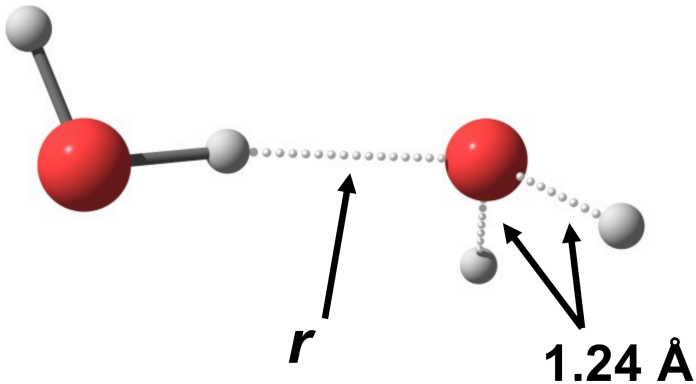

Supplement: SC-014-D2SC05896K-s001 [file SC-014-D2SC05896K-s001.zip › Manuscript_tex/Figures/H2O_xyz.pdf]

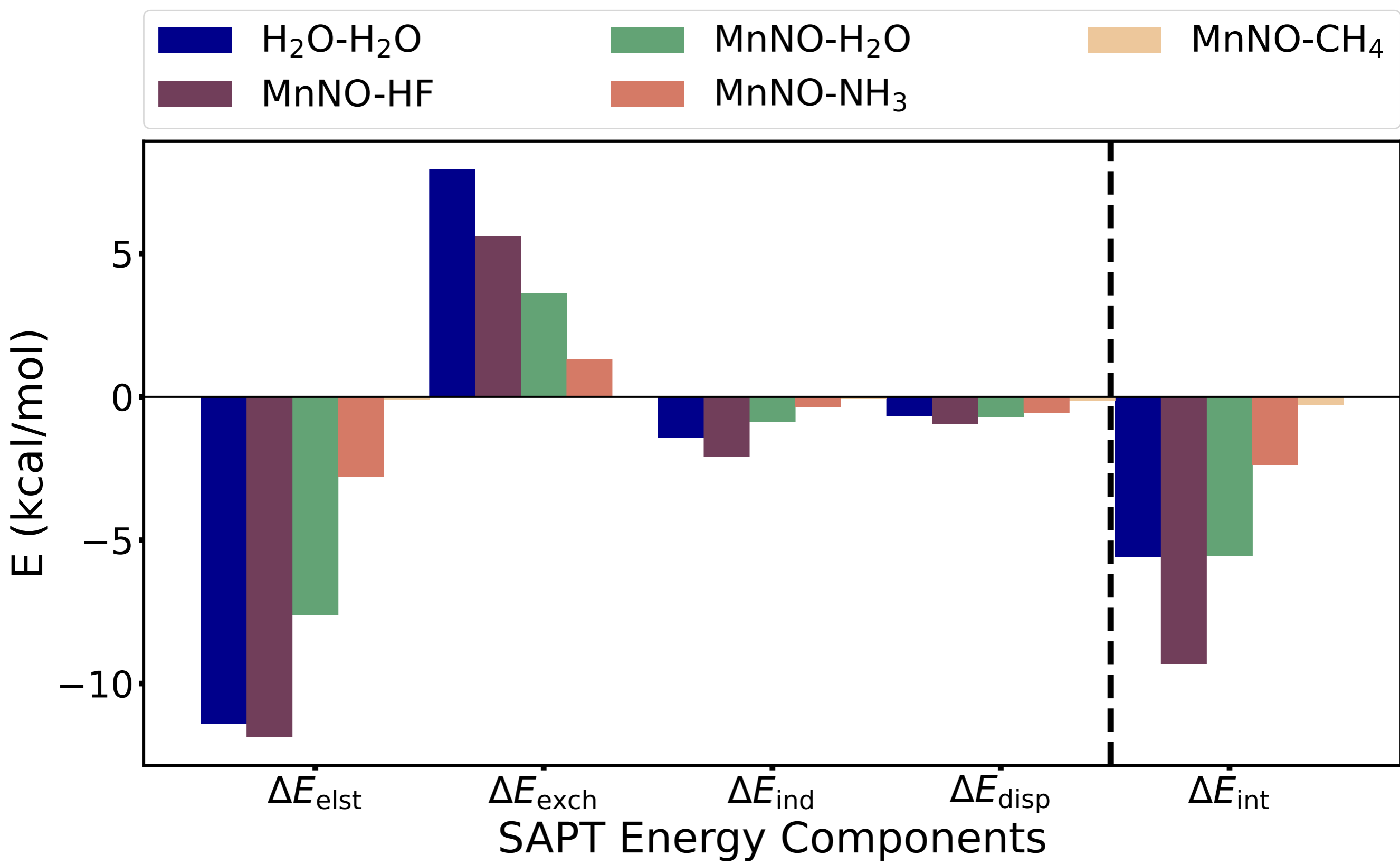

Supplement: SC-014-D2SC05896K-s001 [file SC-014-D2SC05896K-s001.zip › Manuscript_tex/Figures/SAPT_Mn.pdf]

Error  $\varepsilon$  (kcal/mol) p-Bz Bz Dimer

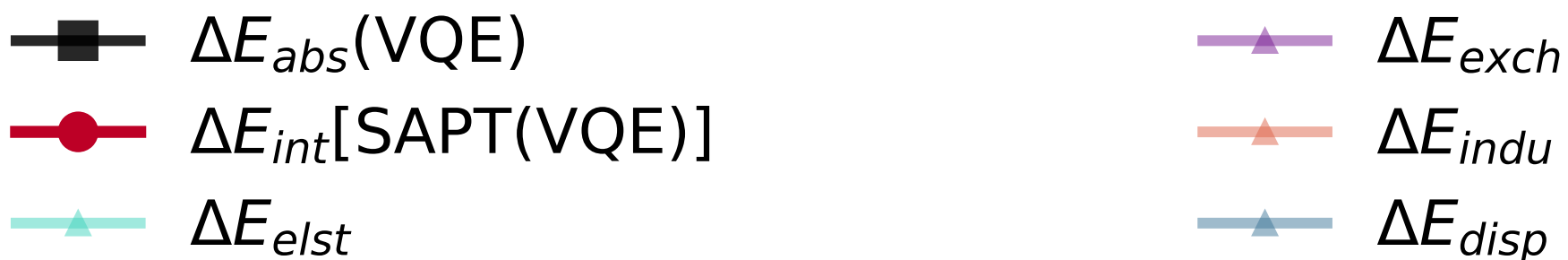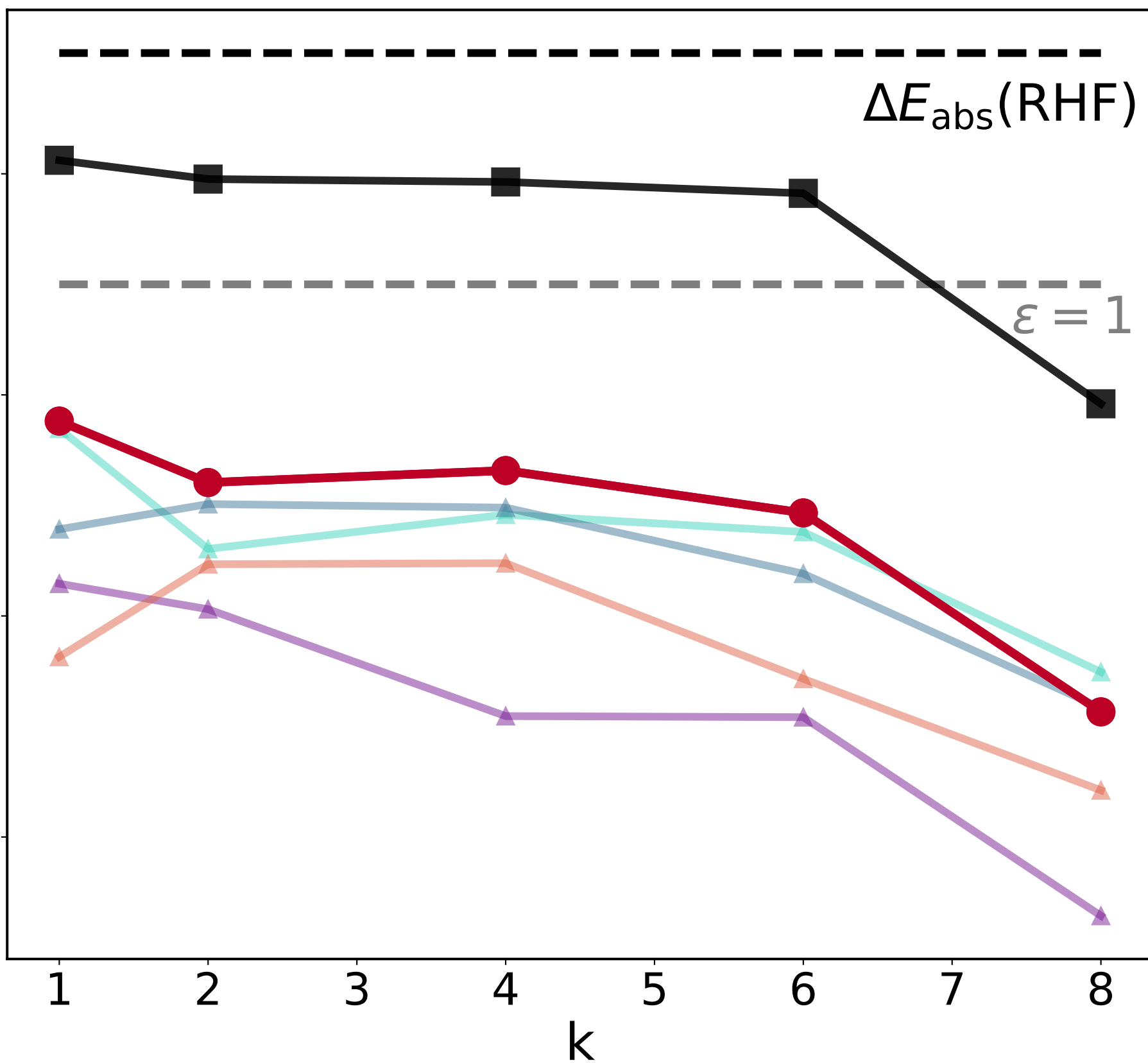

Supplement: SC-014-D2SC05896K-s001 [file SC-014-D2SC05896K-s001.zip › Manuscript_tex/Figures/Error_BZ.pdf]

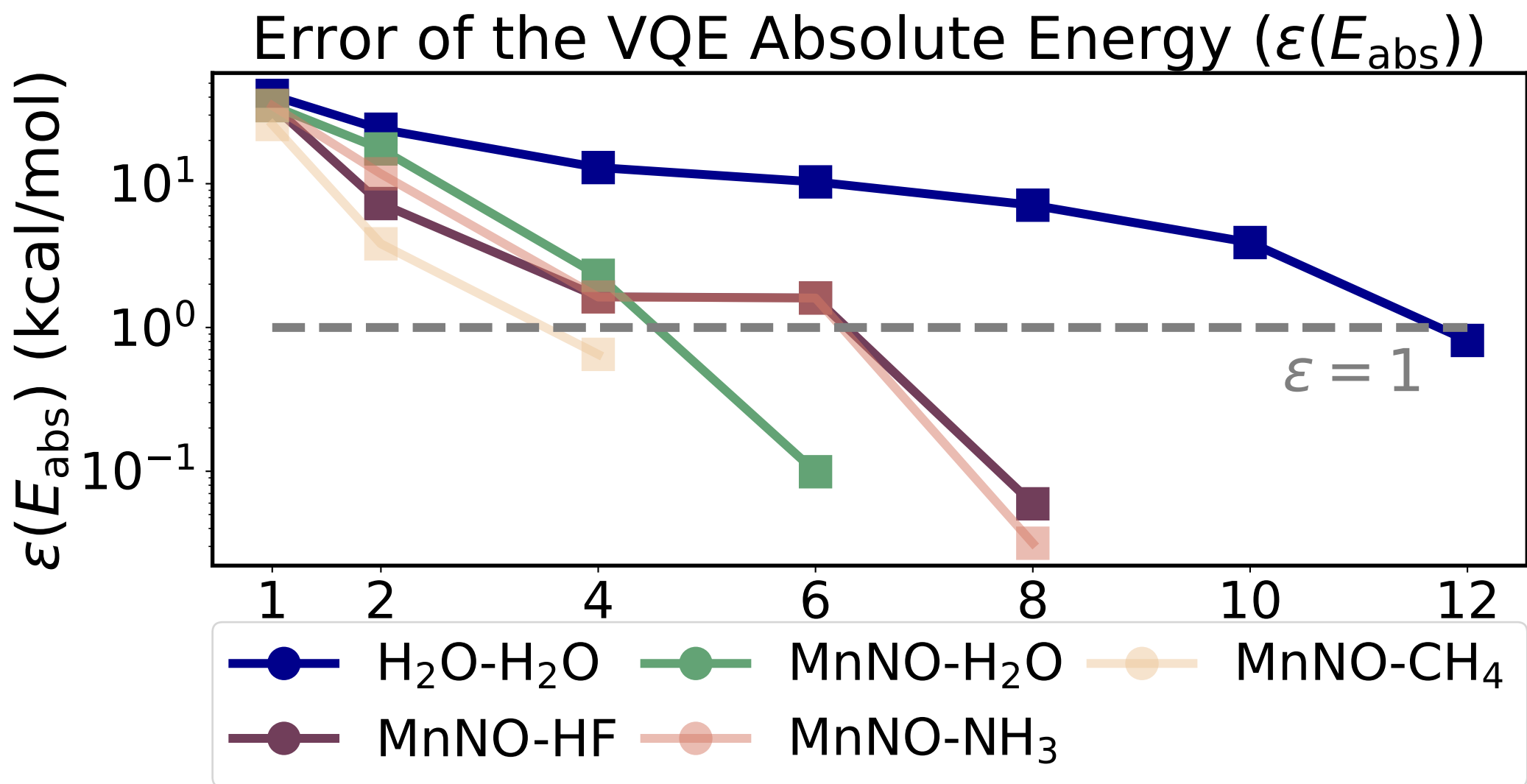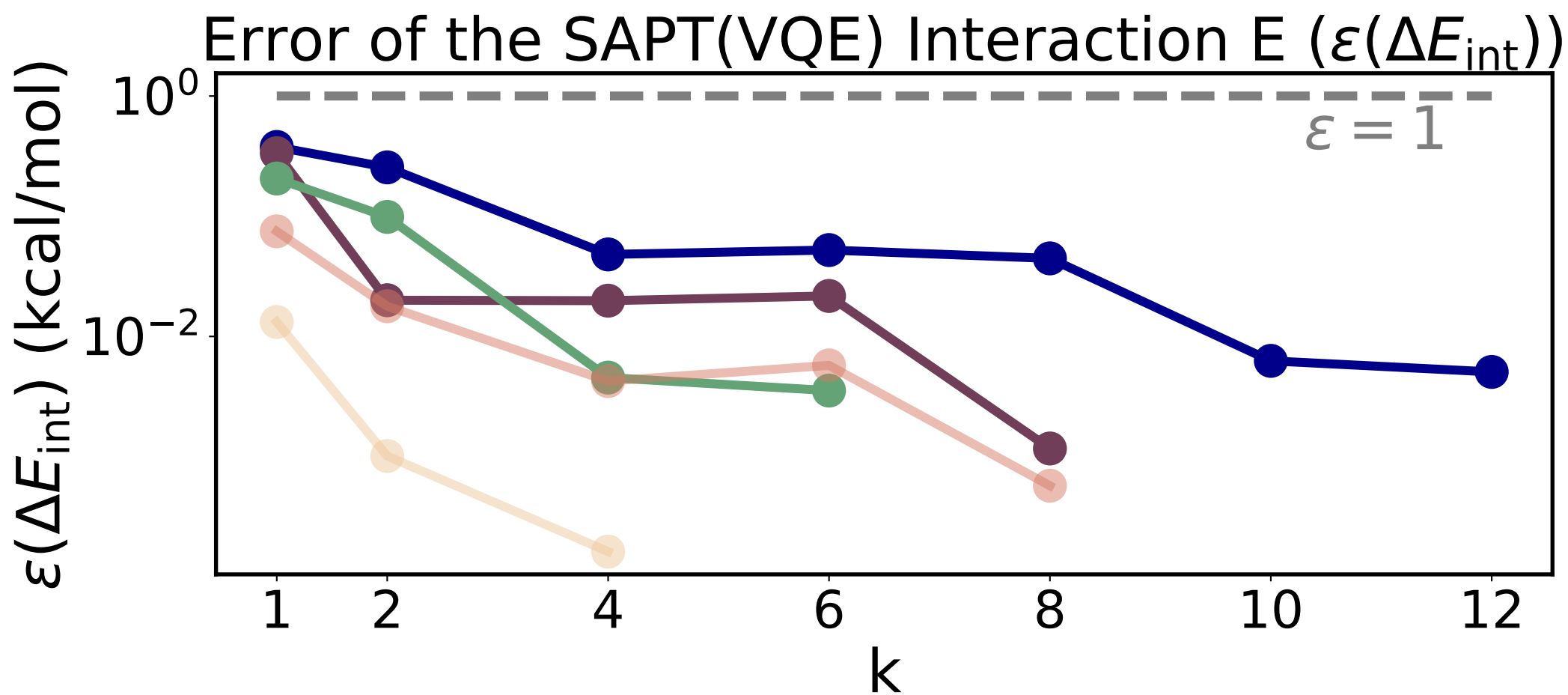

Supplement: SC-014-D2SC05896K-s001 [file SC-014-D2SC05896K-s001.zip › Manuscript_tex/Figures/Error_Mn.pdf]

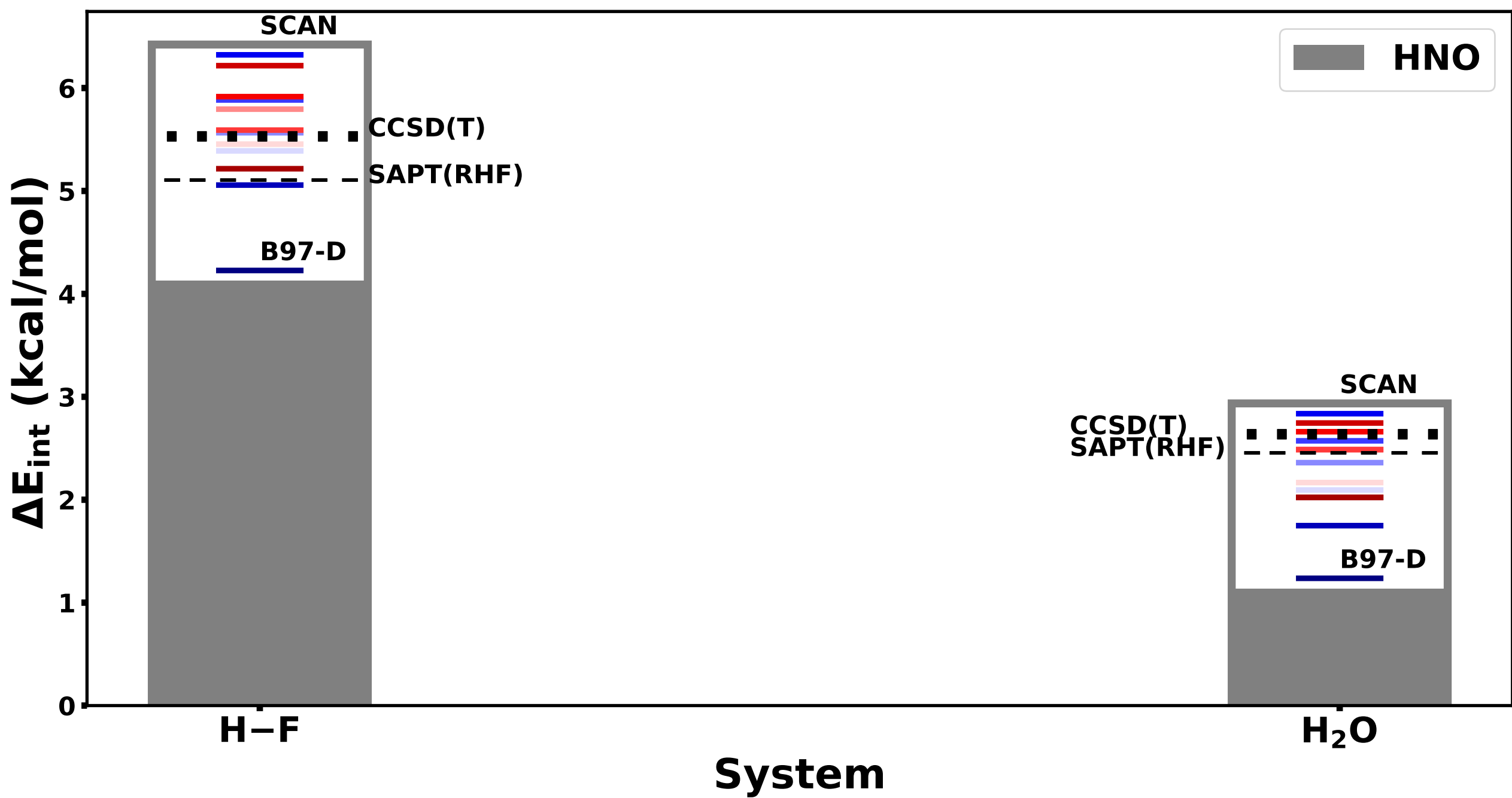

Supplement: SC-014-D2SC05896K-s001 [file SC-014-D2SC05896K-s001.zip › Manuscript_tex/Figures/SAPT_DFT_CCSDT_HNO.pdf]

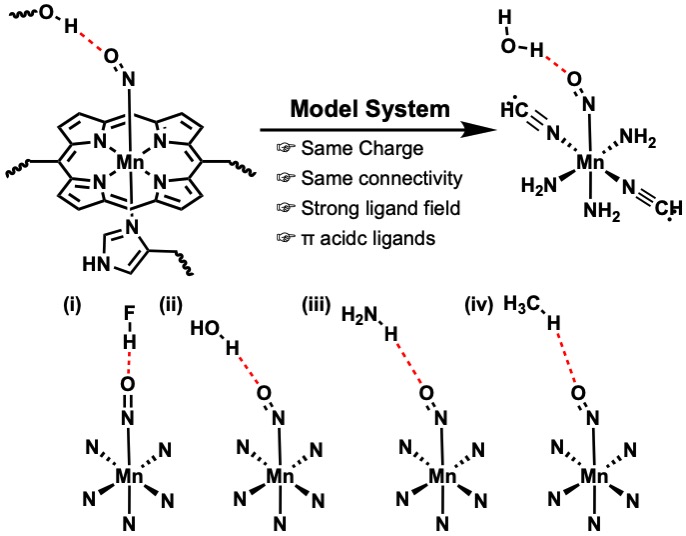

Supplement: SC-014-D2SC05896K-s001 [file SC-014-D2SC05896K-s001.zip › Manuscript_tex/Figures/MnNO_chemdraw.jpeg]

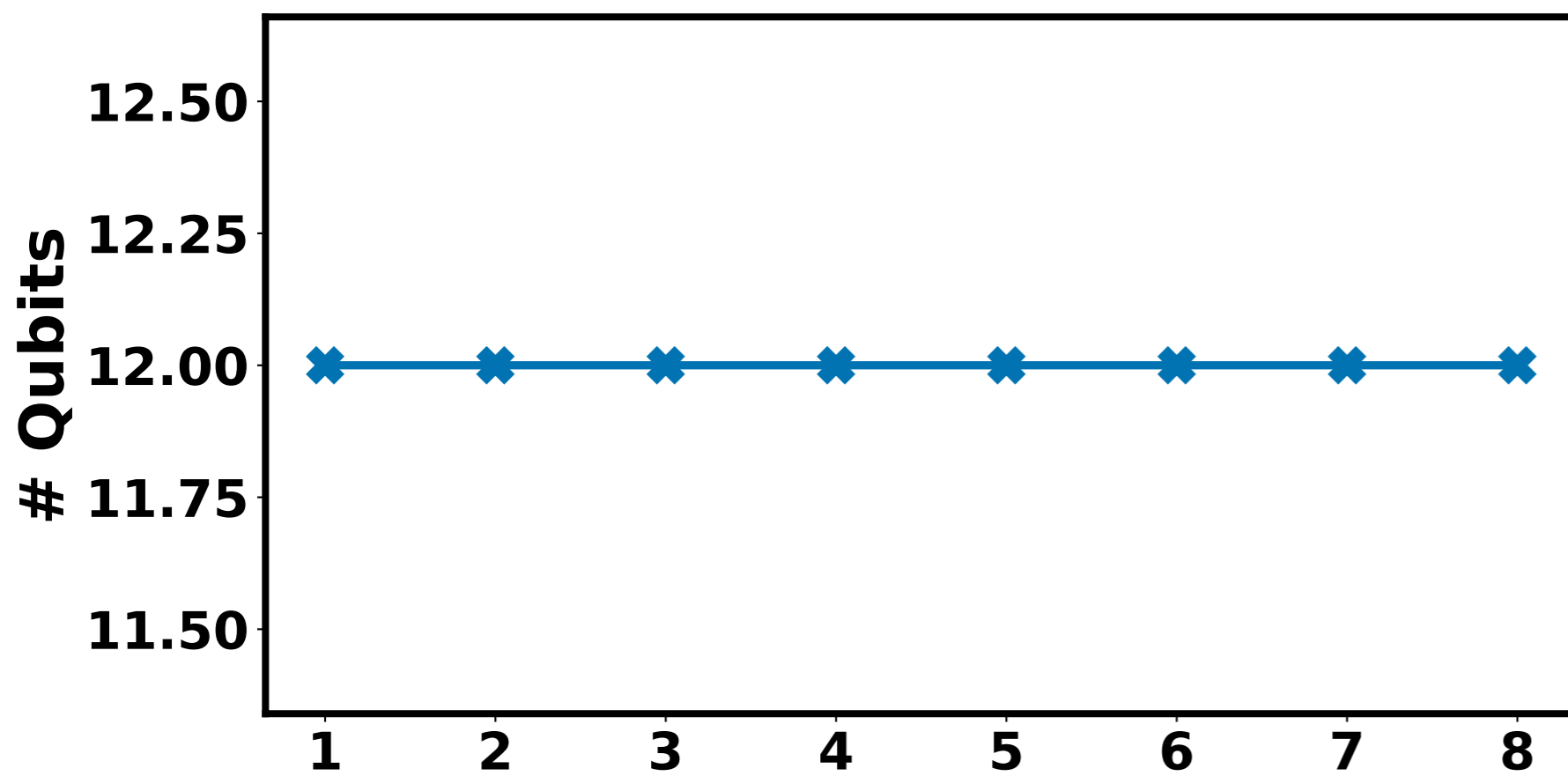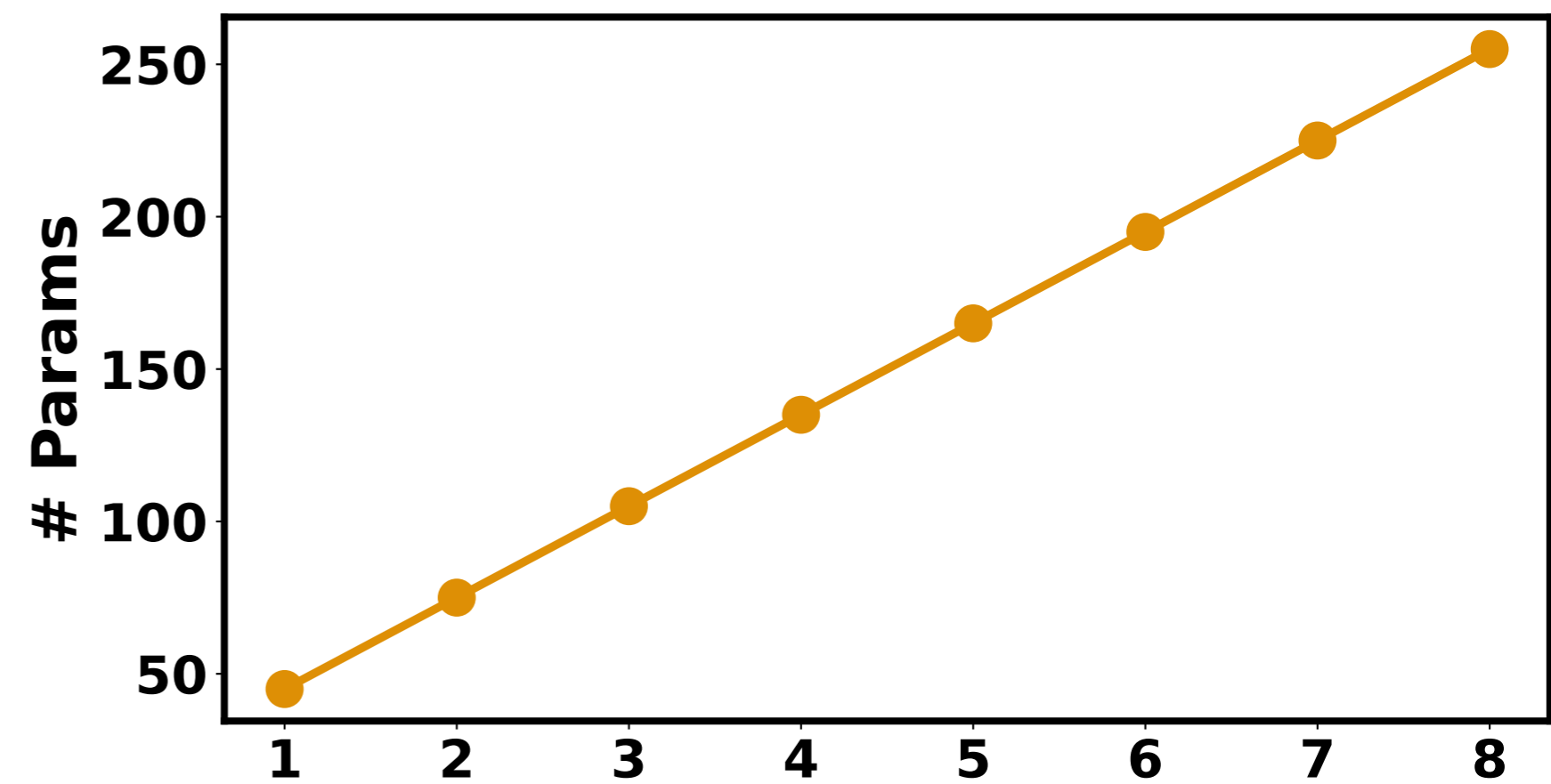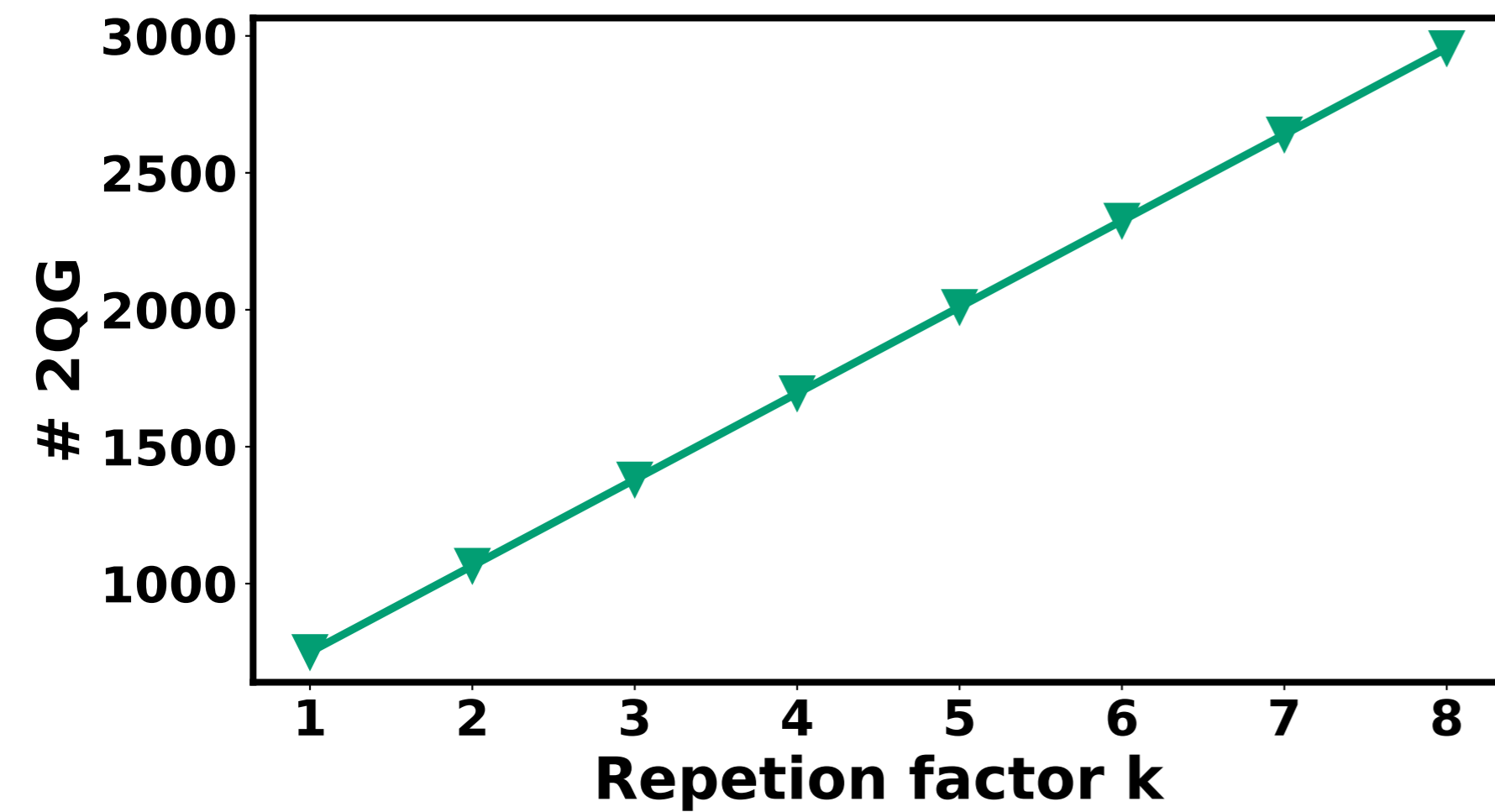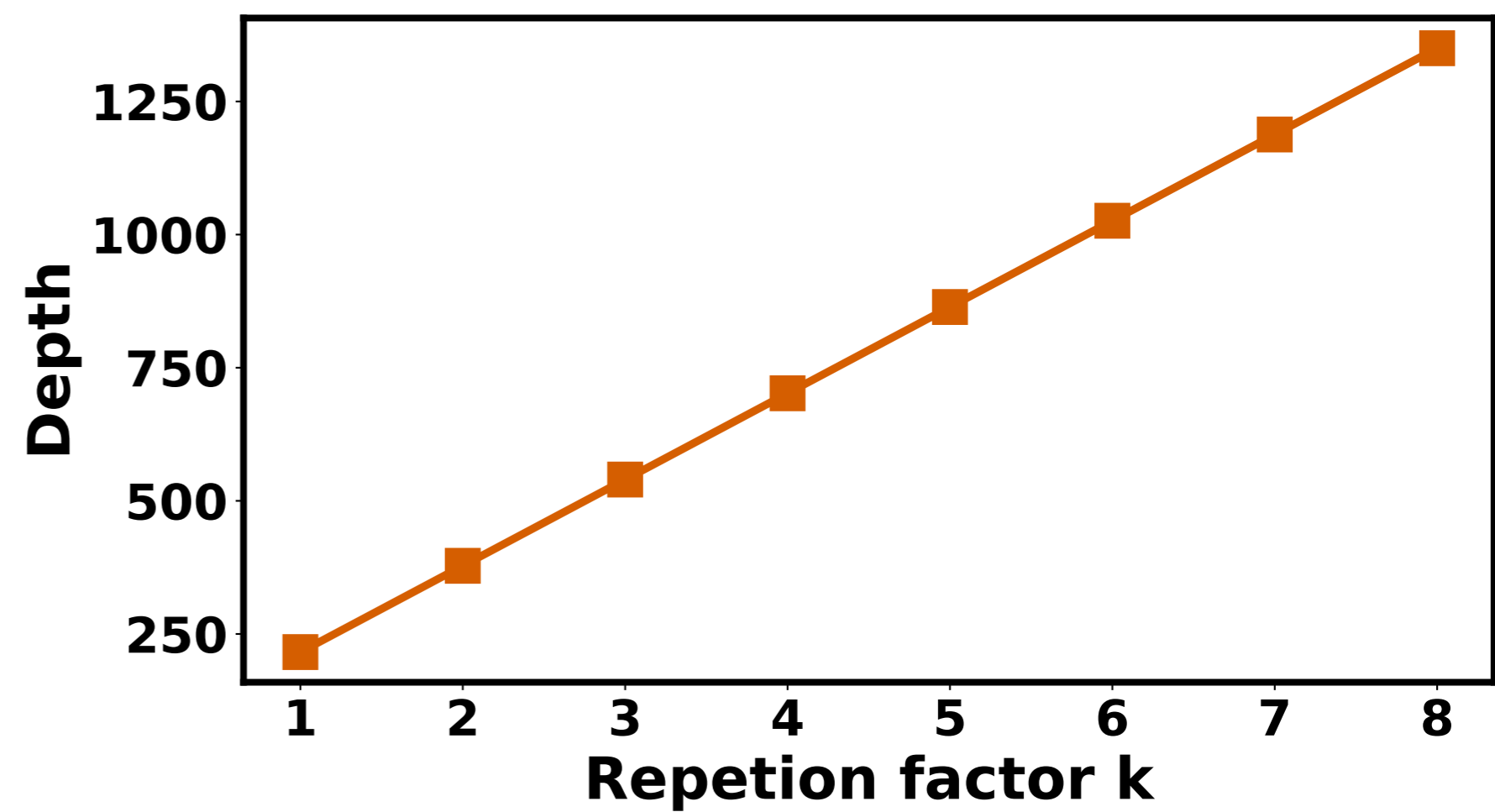

Supplement: SC-014-D2SC05896K-s001 [file SC-014-D2SC05896K-s001.zip › Manuscript_tex/Figures/ressources.pdf]

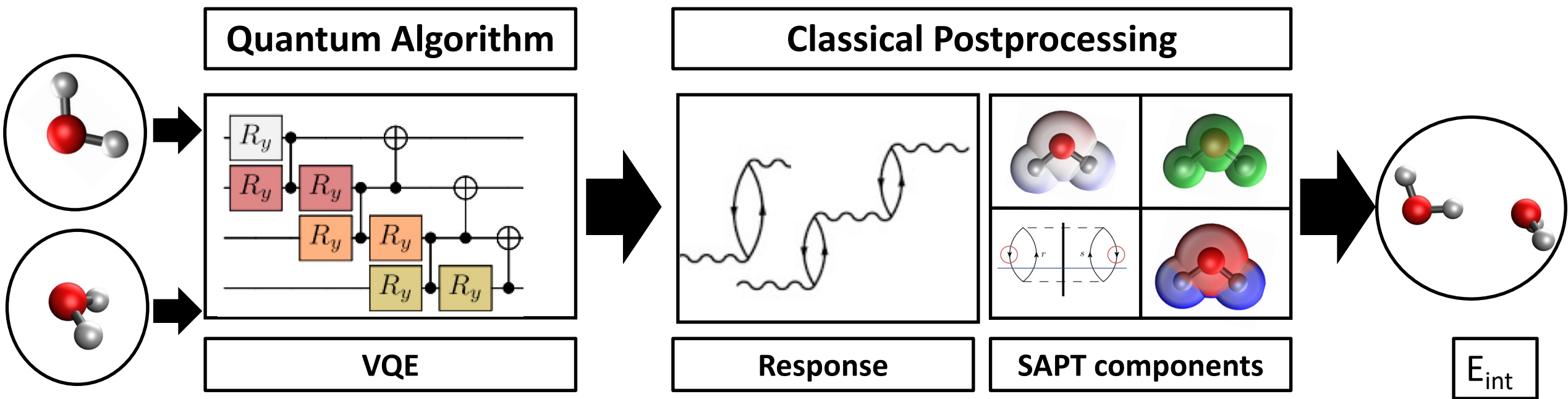

✓ NISQ algorithm

✓ Accurate Interaction Energies

Supplement: SC-014-D2SC05896K-s001 [file SC-014-D2SC05896K-s001.zip › Manuscript_tex/Figures/BI_TOC.pdf]

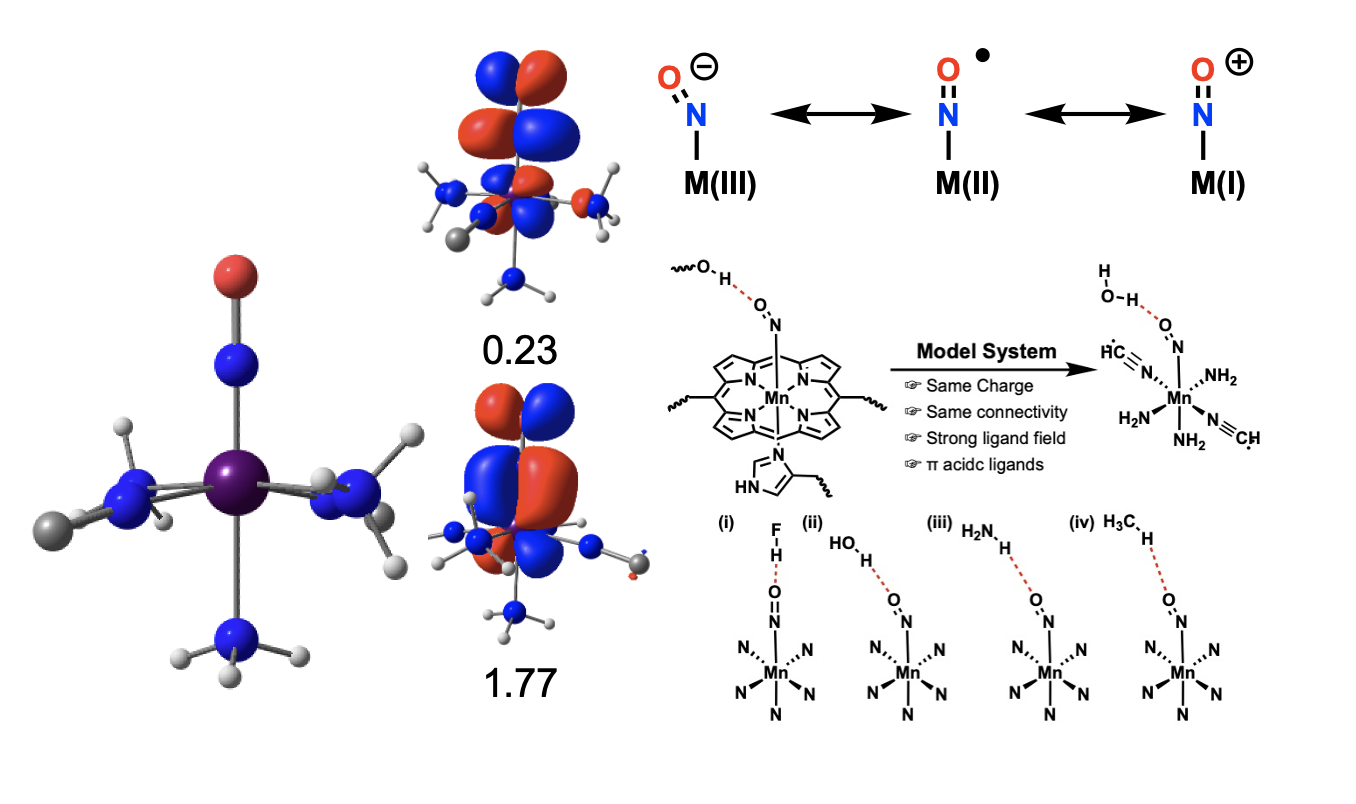

Supplement: SC-014-D2SC05896K-s001 [file SC-014-D2SC05896K-s001.zip › Manuscript_tex/Figures/MnNO_scheme.png]

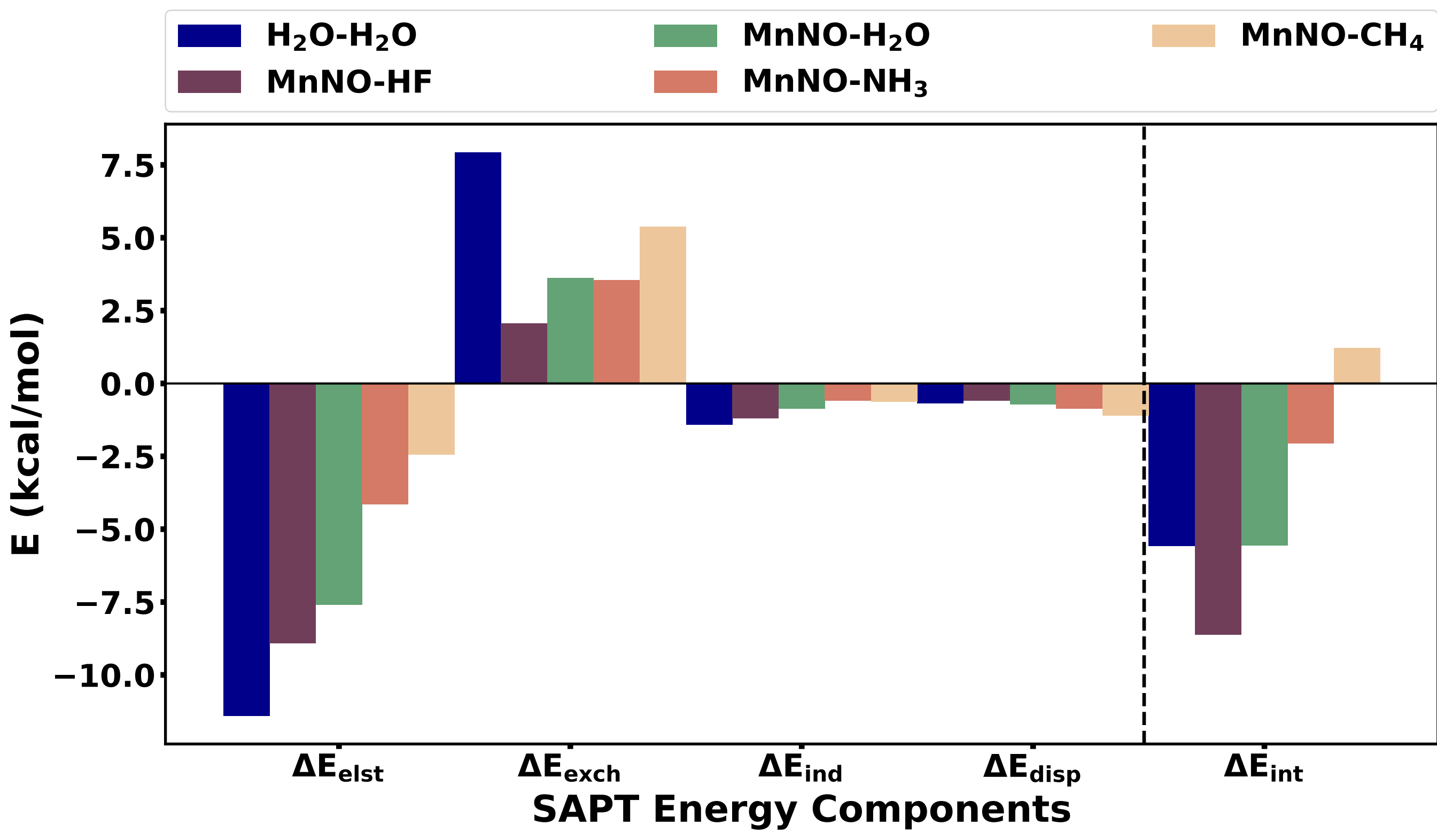

Supplement: SC-014-D2SC05896K-s001 [file SC-014-D2SC05896K-s001.zip › Manuscript_tex/Figures/SAPT_Mn_r_fixed.pdf]

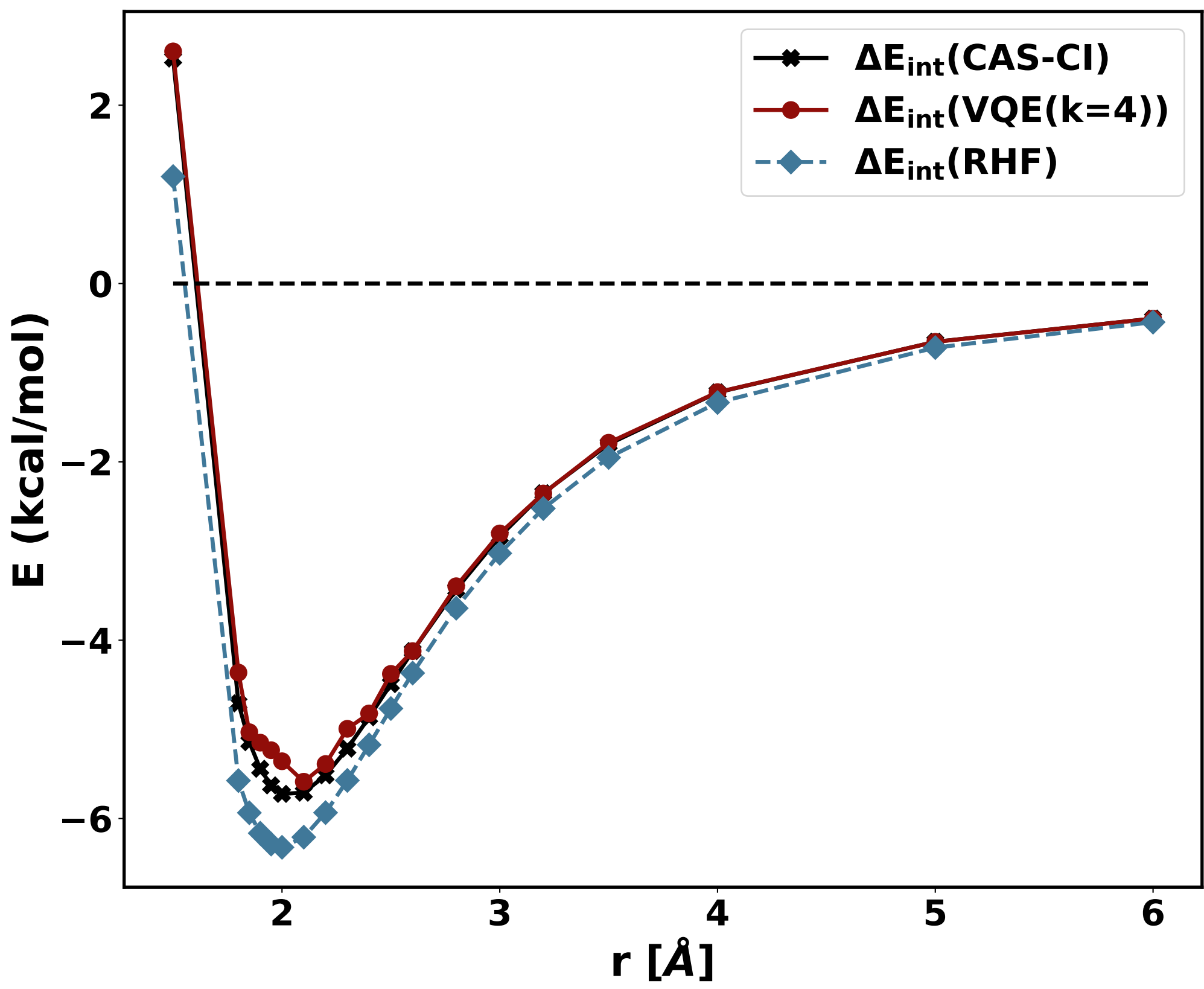

Supplement: SC-014-D2SC05896K-s001 [file SC-014-D2SC05896K-s001.zip › Manuscript_tex/Figures/PES_H2O.pdf]

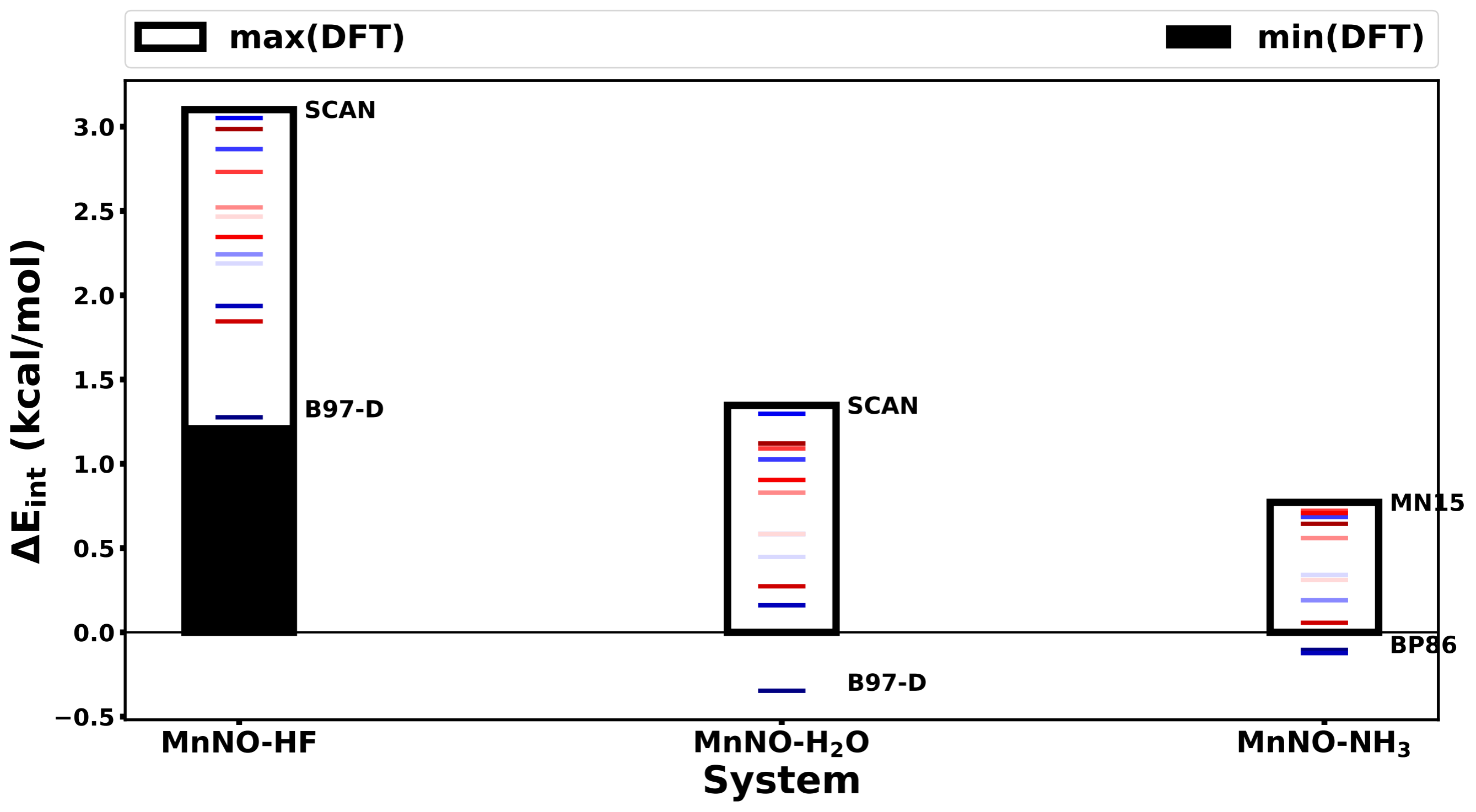

Supplement: SC-014-D2SC05896K-s001 [file SC-014-D2SC05896K-s001.zip › Manuscript_tex/Figures/DFT_large_basis_Mn.pdf]

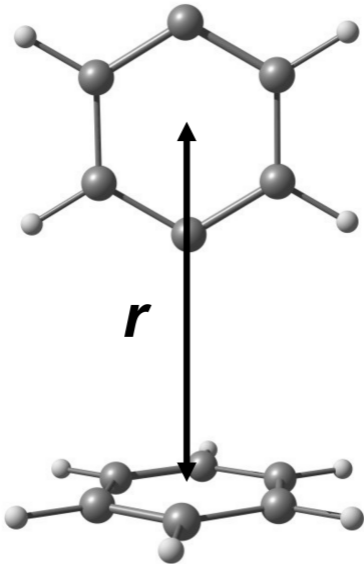

Supplement: SC-014-D2SC05896K-s001 [file SC-014-D2SC05896K-s001.zip › Manuscript_tex/Figures/Bz_xyz.pdf]

Error  $\varepsilon$  (kcal/mol) H<sub>2</sub>O Dimer

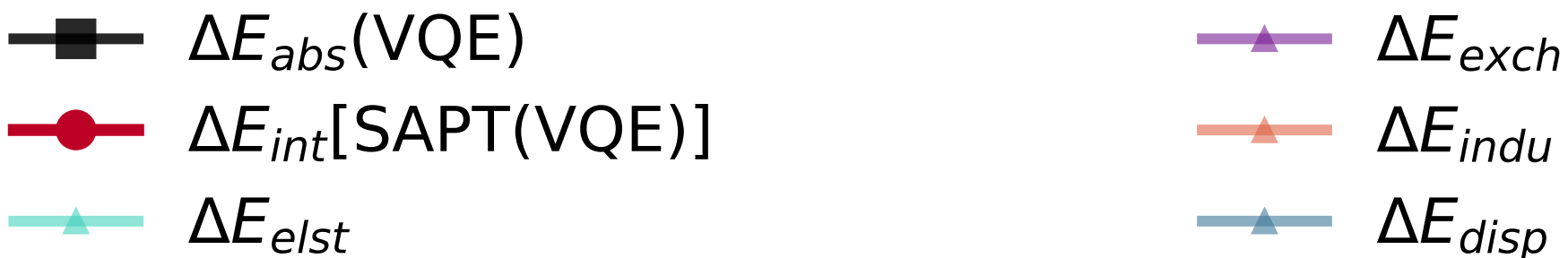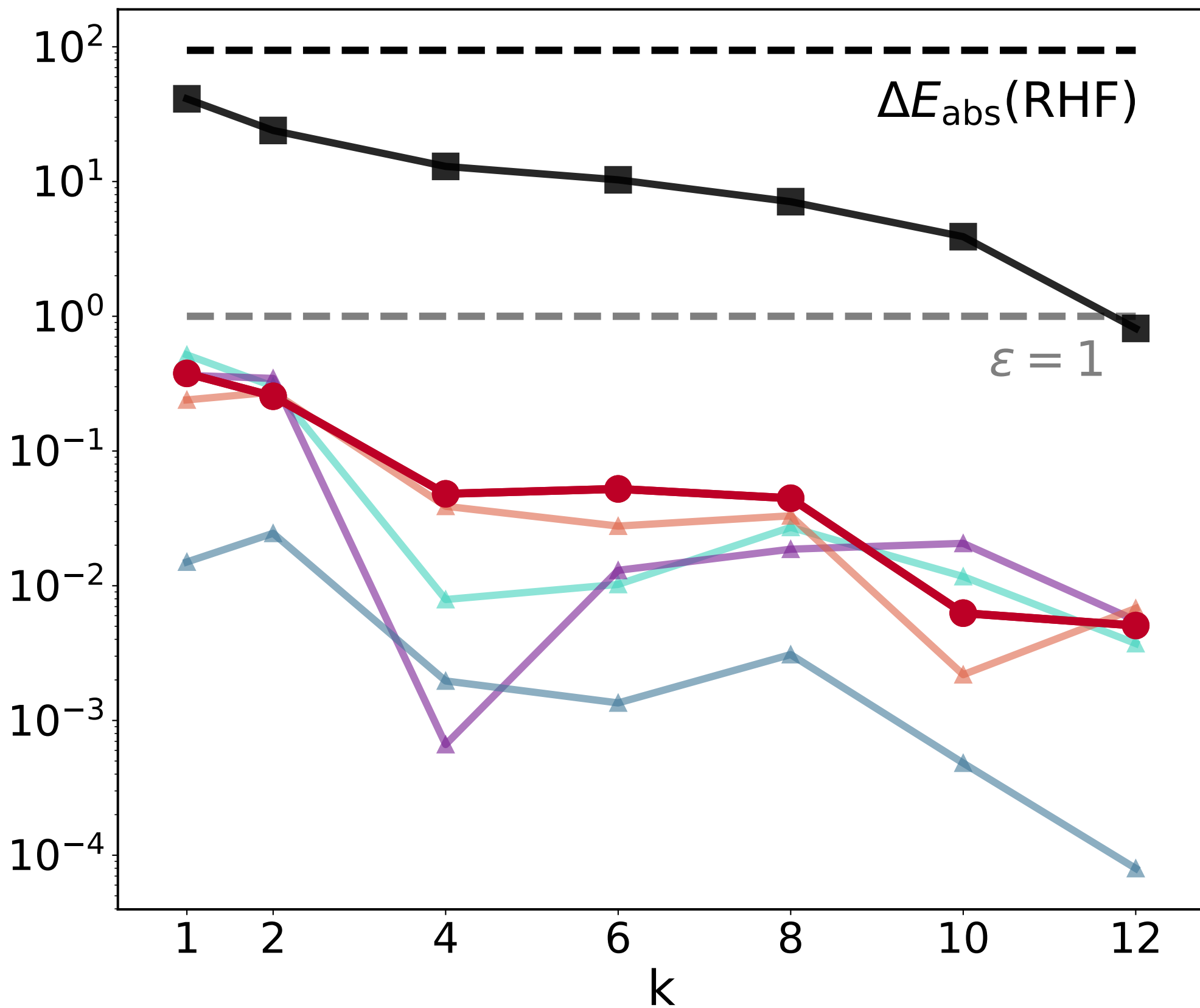

Supplement: SC-014-D2SC05896K-s001 [file SC-014-D2SC05896K-s001.zip › Manuscript_tex/Figures/Error_H2O.pdf]

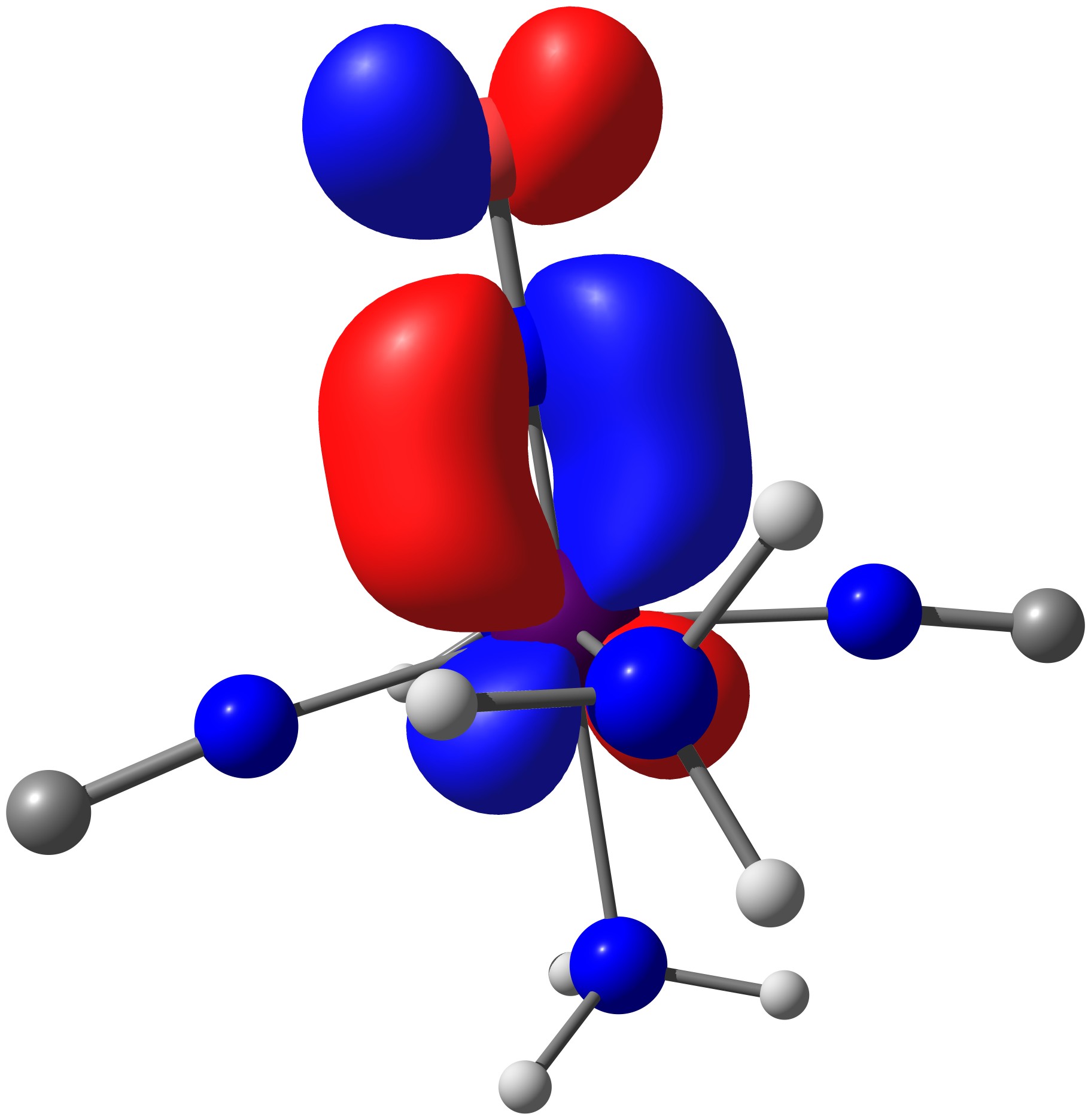

Supplement: SC-014-D2SC05896K-s001 [file SC-014-D2SC05896K-s001.zip › Manuscript_tex/Figures/CASSCF/MO2.jpg]

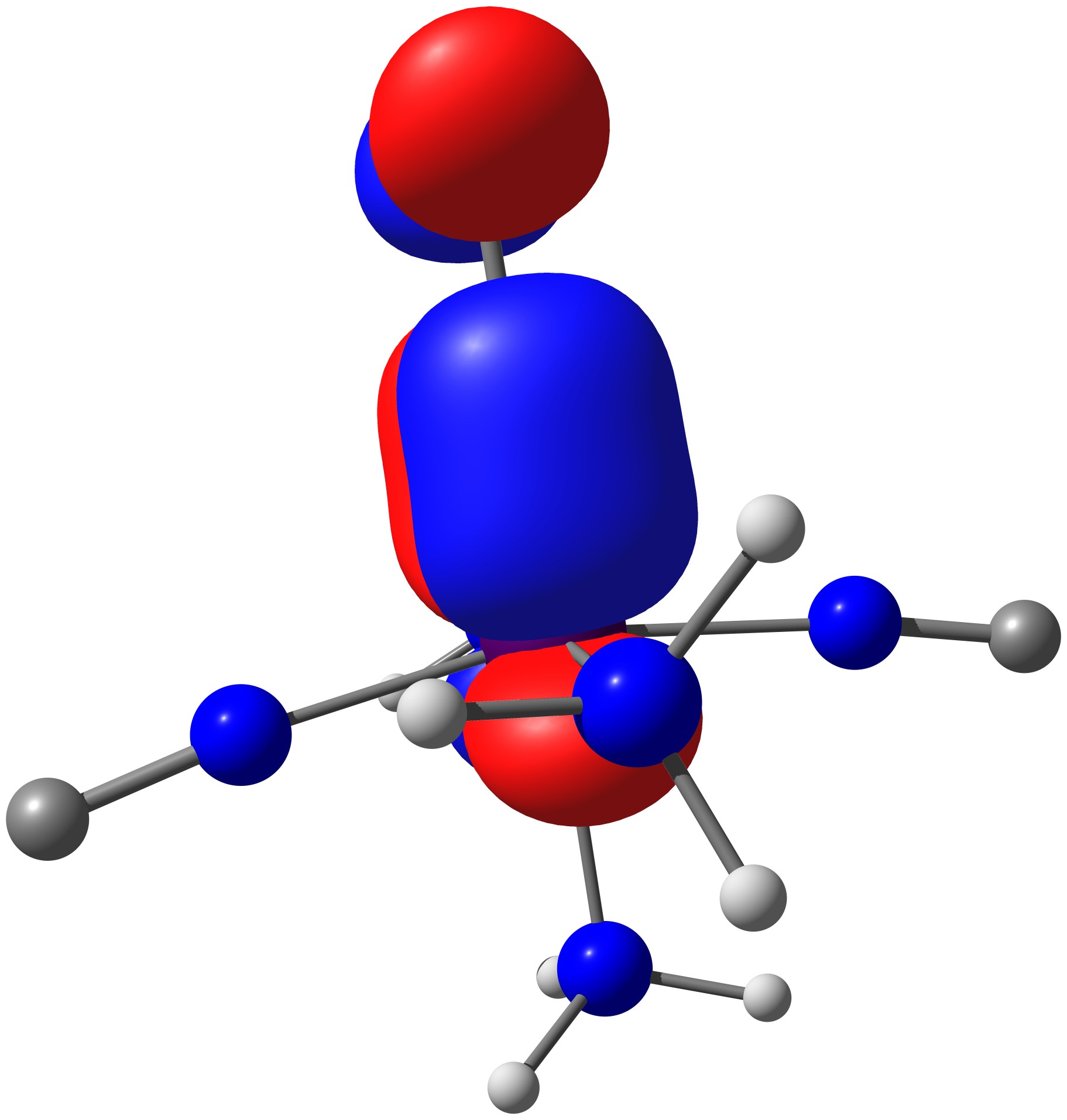

Supplement: SC-014-D2SC05896K-s001 [file SC-014-D2SC05896K-s001.zip › Manuscript_tex/Figures/CASSCF/MO3.jpg]

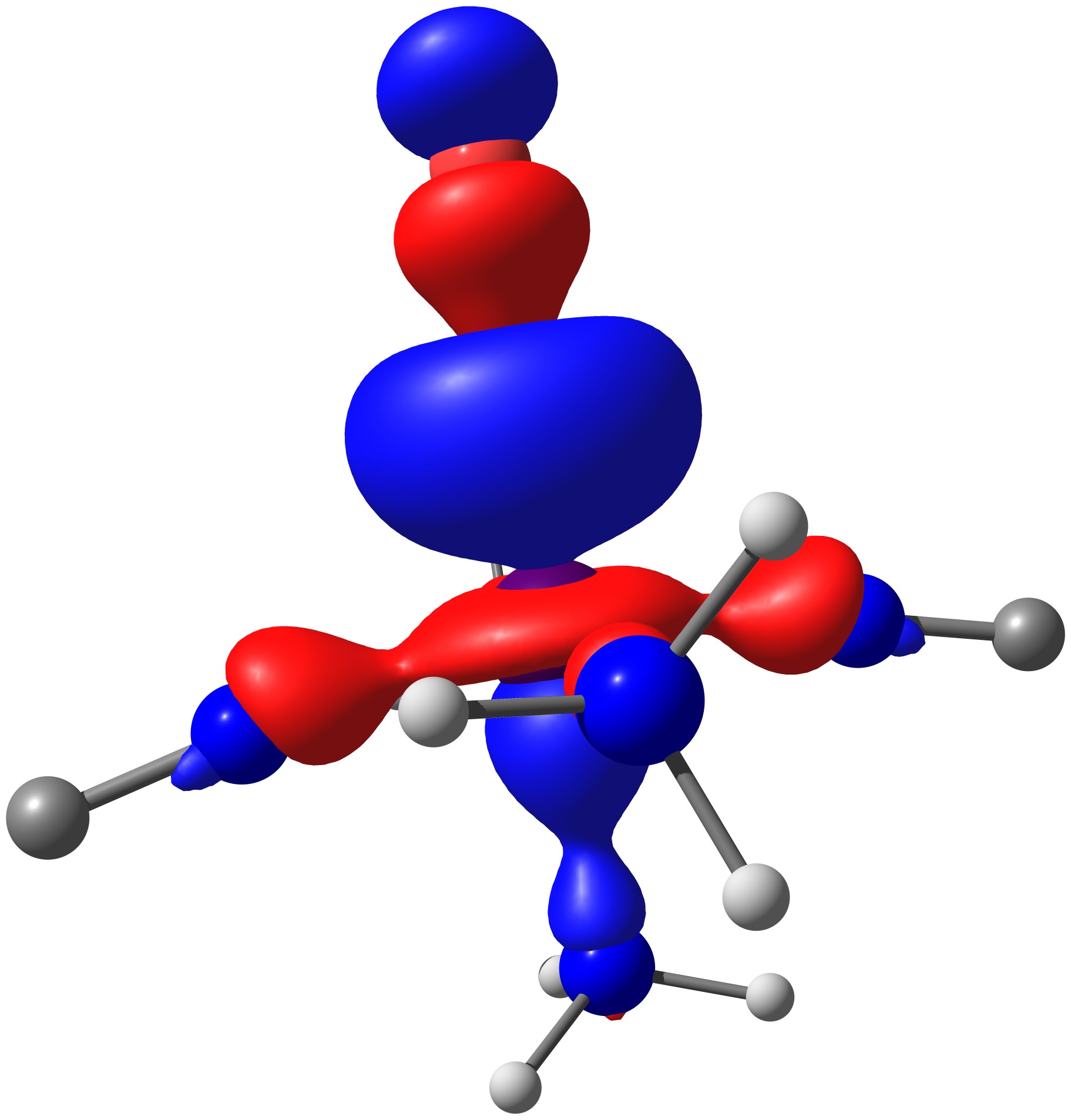

Supplement: SC-014-D2SC05896K-s001 [file SC-014-D2SC05896K-s001.zip › Manuscript_tex/Figures/CASSCF/MO1.jpg]

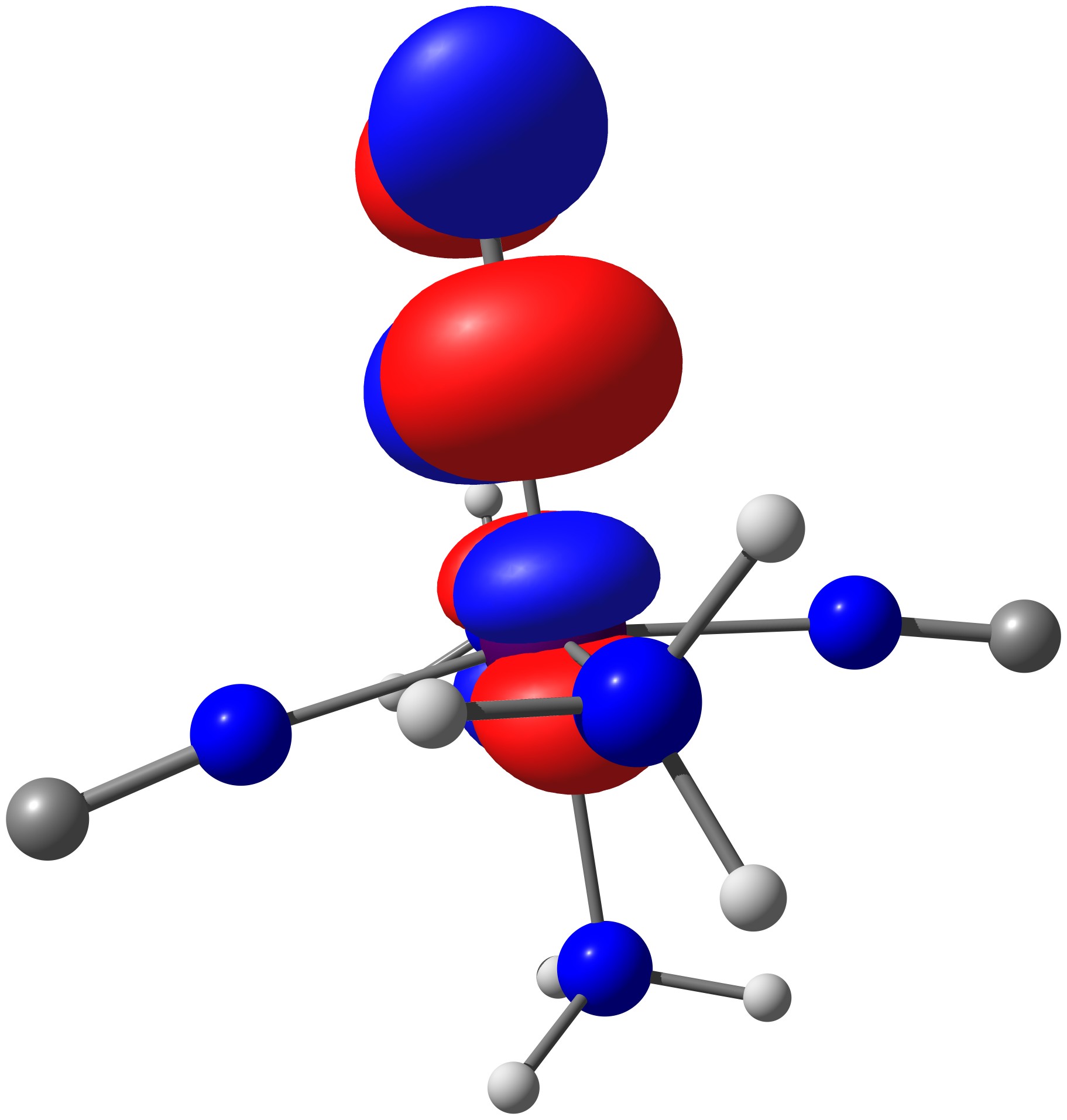

Supplement: SC-014-D2SC05896K-s001 [file SC-014-D2SC05896K-s001.zip › Manuscript_tex/Figures/CASSCF/MO4.jpg]

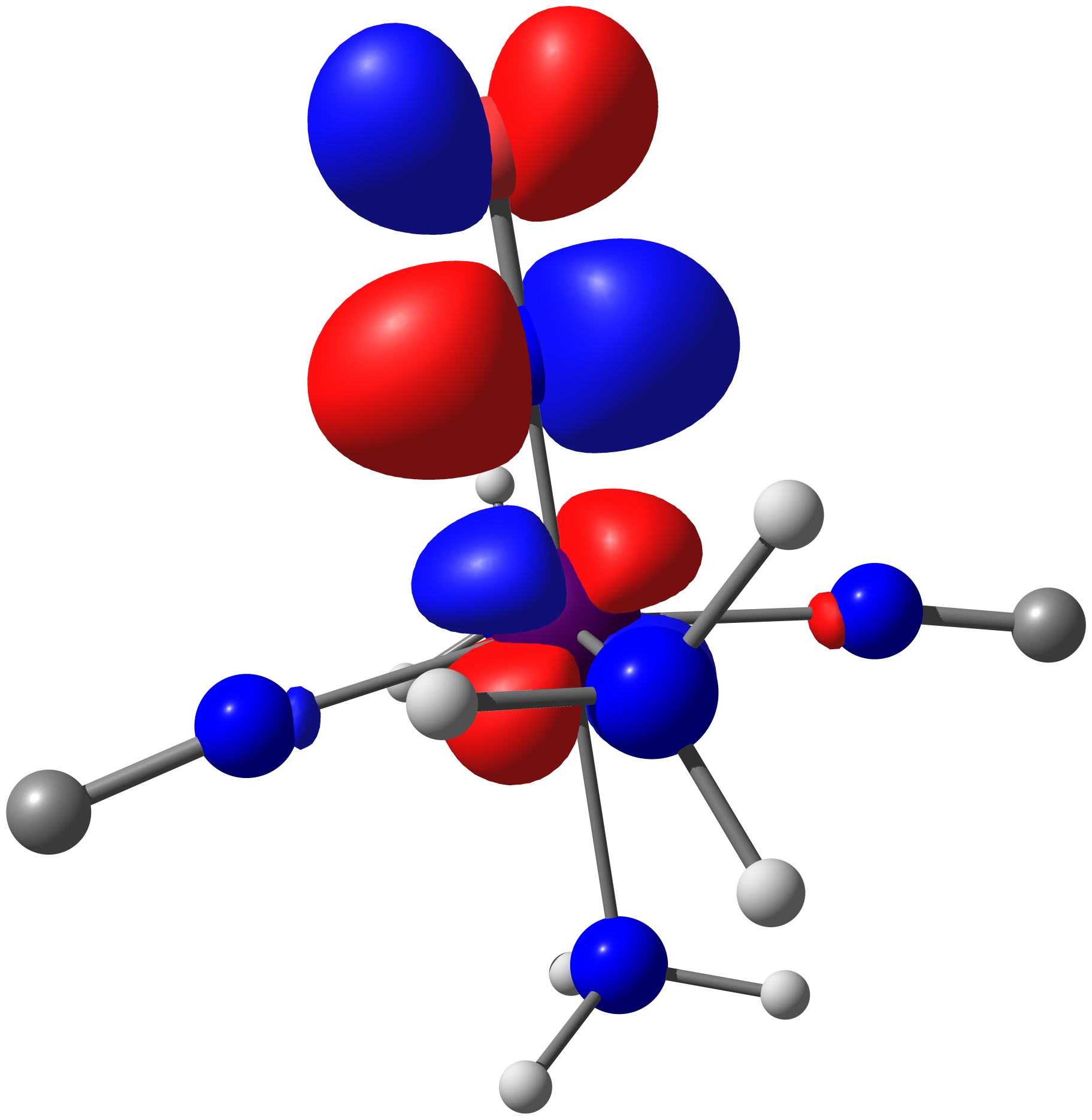

Supplement: SC-014-D2SC05896K-s001 [file SC-014-D2SC05896K-s001.zip › Manuscript_tex/Figures/CASSCF/MO5.jpg]

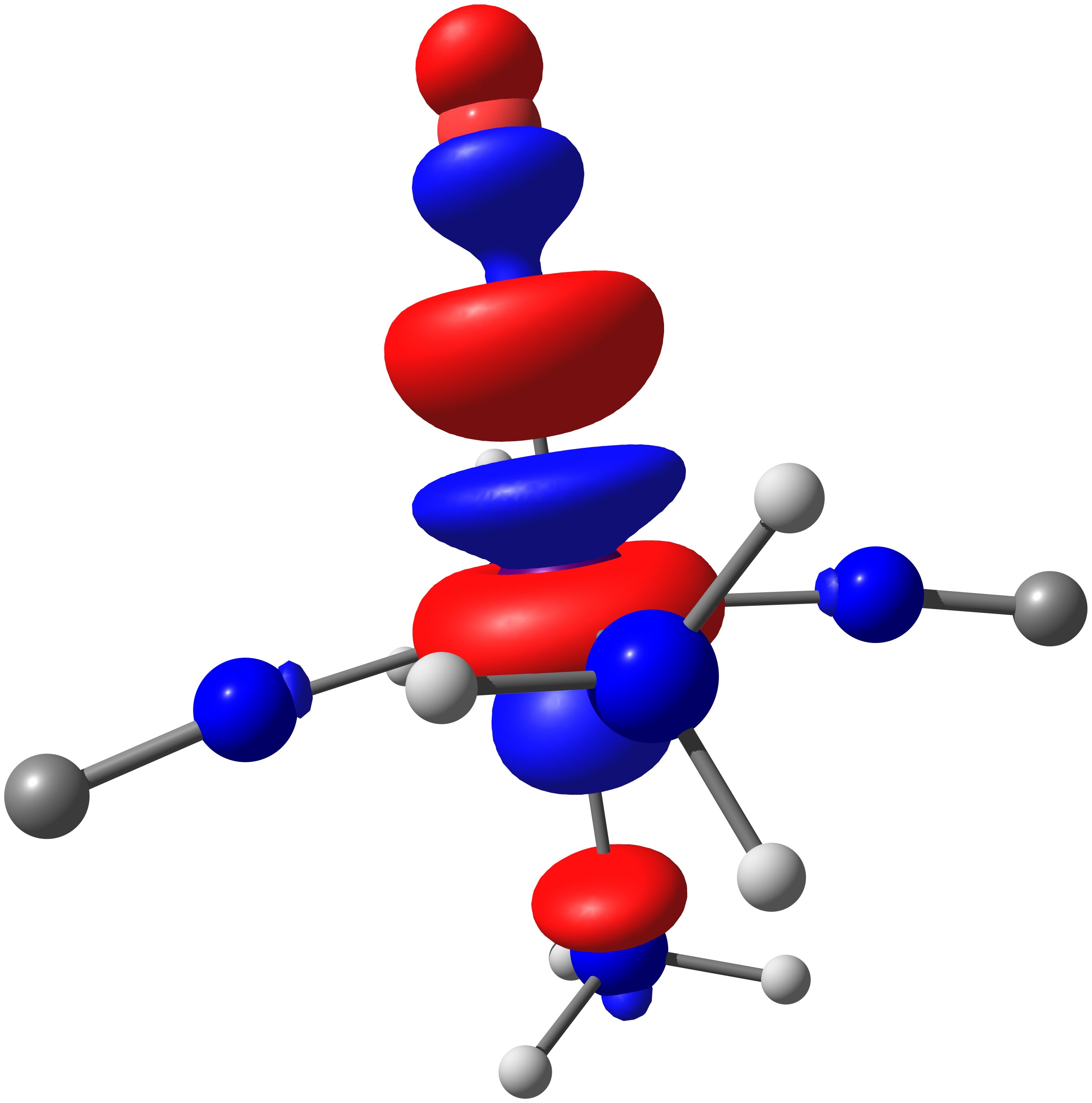

Supplement: SC-014-D2SC05896K-s001 [file SC-014-D2SC05896K-s001.zip › Manuscript_tex/Figures/CASSCF/MO6.jpg]
