# Supplementary material for: Accurate non-covalent interaction energies on noisy intermediate-scale quantum computers via second-order symmetry-adapted perturbation theory
Source: Chem Sci. 2023 Feb 23;14(13):3587–99. doi: 10.1039/d2sc05896k (PMC10055839; doi:10.1039/d2sc05896k)
Supplement: SC-014-D2SC05896K-s002 [file SC-014-D2SC05896K-s002.pdf]

Electronic Supplementary Material (ESI) for Chemical Science.
This journal is © The Royal Society of Chemistry 2023

Supporting Information: Accurate Non-Covalent Interaction Energies on Noisy Intermediate-Scale Quantum Computers via Second-Order Symmetry-Adapted Perturbation Theory

Matthias Loipersberger [†,‖] Fionn D. Malone [†,‡,‖] Alicia R. Welden [†] Robert M. Parrish [*,†] Thomas Fox [¶] Matthias Degroote [§] Elica Kyoseva [§] Nikolaj Moll [*,§] Raffaele Santagati [§] and Michael Streif [§]

†*QC Ware Corporation, Palo Alto, CA, 94301, USA*

‡*Present address: Google Quantum AI, Mountain View, CA, 94043*

¶*Medicinal Chemistry, Boehringer Ingelheim Pharma GmbH & Co. KG, Birkendorfer Straße 65, 88397 Biberach an der Riß, Germany*

§*Quantum Lab, Boehringer Ingelheim, 55218 Ingelheim am Rhein, Germany*

‖*These authors contributed equally to this work.*

E-mail: rob.parrish@qcware.com; nikolaj.moll@boehringer-ingelheim.com

1 Theory

1.1 Indices and Labels

We adopt the following notation for orbital sets used in this work:

- A/B - monomer nuclear indices.
- μ/ν - nonorthogonal atomic spatial orbital basis indices (i.e., Gaussian basis indices).
- p/q - orthogonal molecular spatial orbital basis indices.
- i/j - orthogonal occupied spatial orbital basis indices.
- t/u - orthogonal active spatial orbital basis indices.
- a/b - orthogonal virtual spatial orbital basis indices.

Repeated indices within a monomer will be denoted with primes, e.g., p, p', p'', p'''. When dealing with spin-orbital quantities, we use the context specific notation of an "unbarred" orbital index to denote α and a "barred" orbital index to denote β, i.e., $p^\dagger$ is an α spin-orbital creation operator on spatial orbital index p, while $\bar{p}^\dagger$ is a β spin-orbital creation operator on spatial orbital index p.

1.2 Symmetry Adapted Perturbation Theory

The interaction energy between two monomers is defined as the total energy of the combined system minus the total energies of the two separated monomers:

$$E_{\text{int}} = E_{AB} - E_A - E_B, \tag{1}$$

where the total energies represent the full CI solution at the basis set limit. In practice, approximate methods such as density functional theory or coupled cluster methods are used to compute accurate enough total energies to resolve the binding energy accurately.

An alternative approach to computing intermolecular interaction energies is symmetry adapted perturbation theory (SAPT) which is valid for non-covalent interactions. Instead of computing total energies, SAPT assumes that the intermolecular interactions are weak and thus we can compute the interaction energy via perturbation theory. In particular, we can write the Hamiltonian of the combined system as

$$\hat{H} = \hat{H}_A + \hat{H}_B + \hat{V}, \tag{2}$$

where we assume $\hat{H}_X|\Psi_X\rangle = E_X|\Psi_X\rangle$, where $|\Psi_X\rangle$ is the ground state wavefunction of monomer X and $\hat{V}$ contains only the Coulombic interactions between monomer A and B. With this partitioning of the Hamiltonian, we can build a perturbation theory for the intermolecular interaction energy directly thus avoiding computing potentially very large total energies. More explicitly we have

$$E_{\text{int}} = \sum_n (E^{(n)}_{\text{pol}} + E^{(n)}_{\text{exch}}), \tag{3}$$

where $E^{(n)}_{\text{pol}}$ and $E^{(n)}_{\text{exch}}$ are nth-order polarization and exchange energies respectively. An added benefit of SAPT is that we obtain an intuitive breakdown of the interaction energy components, into electrostatic, induction, dispersion and exchange components which can be used to provide chemical insight into the binding process. Truncation after the second order gives rise to SAPT[S1] yielding:

$$E_{\text{int}} \approx E_{SAPT} = E^{(1)}_{\text{elst}} + E^{(1)}_{\text{exch}} + E^{(2)}_{\text{ind}} + E^{(2)}_{\text{disp}} + E^{(2)}_{\text{exch-disp}} + E^{(2)}_{\text{exch-ind}}, \tag{4}$$

where $E^{(1)}_{\text{elst}}$ corresponds to the first order electrostatics term, $E^{(1)}_{\text{exch}}$ to the first order exchange term, $E^{(1)}_{\text{ind}}$ to the first order induction term, $E^{(2)}_{\text{disp}}$ to the second order dispersion term, $E^{(2)}_{\text{exch-disp}}$ to the second order exchange-dispersion term, $E^{(2)}_{\text{exch-ind}}$ to the second order exchange-induction term (see below for a detailed derivation). In the main manuscript, we

combine both dispersion and inductions terms and drop the superscripts yielding:

$$E_{\text{int}} \approx E_{\text{elst}} + E_{\text{exch}} + E_{\text{ind}} + E_{\text{disp}}. \tag{5}$$

Ref. S2 shows that this level of SAPT (SAPT0) using Hartee-Fock wavefunctions and a medium-size jun-cc-pvdz basis can yield highly accurate results for a broad range of non-covalent interactions. We discuss the relationship between the different SAPT methods in section 3.5

As in-depth discussed in our previous work,[S3] we use the density matrix formulation of SAPT[S4–S7] as recently fully implemented for complete active space self-consistent field (CASSCF) wavefunctions by Hapka and *et al.*[S8] This formalism allows for the evaluation of the terms appearing in Eq. (4) using just the ground state one- and two-particle reduced density matrices of the monomers with additional response terms for the second order terms. Instead of Hartee-Fock density matrices, we use a quantum computer via a VQE ansatz to determine an accurate ground state wave function of the system (for more details on those algorithms, see the next sections). The detailed derivation of the first order terms ($E^{(1)}_{\text{elst}}$, $E^{(1)}_{\text{exch}}$) be found in Ref.[S3]

The second order SAPT terms for induction and dispersion energies (as well as their exchange counterparts) requires the calculation of excited state properties on a quantum computer (see below for a more detailed derivation). Although several approaches have been suggested in the literature to compute excited state properties on NISQ-era quantum computers, they often require a significant measurement overhead.[S9–S12] To reduce this burden we employ the extended random phase approximation (ERPA),[S13] which requires only the one- and two-body reduced density matrices to be evaluated on the quantum computer.[S14] This approximation has previously been shown by others to produce quite accurate interaction energies when employed in SAPT based on CASSCF wavefunctions, and we will show that this carries over to VQE wavefunctions.

Given that NISQ-era devices are currently limited to tens of qubits (spin-orbitals), we will use an active space approach analogous to CAS-CI methods. In the active space approach, we partition the one-electron orbital set into N_c core orbitals, N_a active orbitals and N_i virtual orbitals. This partitioning gives rise to modified monomer Hamiltonian given by (for example, for monomer A)

$$\begin{aligned}\hat{H}_A{}' = &\sum_{tt'\sigma} \tilde{h}_{tt'} a^\dagger_{t\sigma} a_{t'\sigma} + \\ &\frac{1}{2} \sum_{\sigma\sigma'} \sum_{tt't''t'''} (tt''|t't''') a^\dagger_{t\sigma} a^\dagger_{t'\sigma'} a_{t'''\sigma'} a_{t''\sigma}\end{aligned}, \tag{6}$$

where the modified one-electron integrals $\tilde{h}_{tt'}$ now include core-active space interactions

$$\tilde{h}_{tt'} = h_{tt'} + \sum_{ii'} \left[(tt'|ii') - \frac{1}{2}(ti'|it') \right] \gamma_{ii'}. \tag{7}$$

The key approximation in the active space approach is that a (small) set of "active" orbitals and electrons are defined *a priori*, and the FCI expansion is constrained to that subset of electrons and orbitals. The quality of the CAS-CI and VQE results depends strongly on the selected active space.[S15–S17]

1.3 Variational Quantum Eigensolver

This section briefly summarizes the VQE ansatz used in this work, it is identical to the approach taken in our previous work[S3] and discussed in more detail. The SAPT post processing step only relies on the reduced density matrices which can in principle be generated with a quantum algorithm such as other VQE flavors[S18] or Quantum Krylov methods.[S9]

We generate the active space wave function of the strongly correlated monomer using the VQE ansatz described below (it is also possible that both monomer wave functions are evaluated on the quantum computer, but in practice, usually only one monomer will exhibit strong correlation, and thus require a VQE treatment):

$$|\Psi_{\text{VQE}}\rangle \equiv \hat{U}_{\text{VQE}}|\Phi_I\rangle \tag{8}$$

where $|\Phi_I\rangle$ guess state (typically the Hartree–Fock).

Throughout this study, we only use real active space wavefunction $|\Psi_{\text{VQE}}\rangle$ and they will be a definite eigenfunction of the $\hat{N}_\alpha$, $\hat{N}_\beta$, and $\hat{S}^2$ operators.

The paper is using the following Jordan-Wigner representation:

$$p^{\pm} = \bigotimes_{p'=0}^{p'=p-1} \hat{Z}_{p'}(\hat{X}_p \mp i\hat{Y}_p)/2, \tag{9}$$

$$\bar{p}^{\pm} = \bigotimes_{p'=0}^{p'=N_\alpha-1} \hat{Z}_{p'} \bigotimes_{\bar{p}'=0}^{\bar{p}'=\bar{p}-1} \hat{Z}_{\bar{p}'}(\hat{X}_{\bar{p}} \mp i\hat{Y}_{\bar{p}})/2, \tag{10}$$

where $p^+ = p^\dagger$ and $p^- = p$ and we order the Jordan-Wigner strings in α-then-β order and $\hat{Z}, \hat{Y}$ and $\hat{X}$ are the usual Pauli operators.

In this work, we use a modified version of the unitary cluster Jastrow wavefunction[S19] (k-uCJ) which takes the form

$$|\Psi_{\text{VQE}}\rangle = \prod_k \exp(-\hat{K}^{(k)}) \exp(\hat{T}^{(k)}) \exp(+\hat{K}^{(k)})|\Phi_I\rangle, \tag{11}$$

where $\hat{K}^{(k)}$ and $\hat{T}^{(k)}$ are one- and two-body operators and k is a parameter that controls the depth of the circuit and as a result its variational freedom. The key difference in our k-uCJ ansatz from Ref. S19 is that the two-body operator and the restriction to real anti-symmetric matrices.

The one-body orbital transformations (spin restricted) are defined as

$$\hat{K}^{(k)} \equiv \sum_{pp'} \kappa_{pp'}^{(k)} \left[(p^\dagger p' - p'^\dagger p) + (\bar{p}^\dagger \bar{p}' - \bar{p}'^\dagger \bar{p})\right] \tag{12}$$

where $\kappa_{pp'}^{(k)} = -\kappa_{p'p}^{(k)}$ is a real, antisymmetric $N_a \times N_a$ matrix of orbital rotation generators,

which is equivalent to the one-particle spin-restricted orbital transformation:

$$U_{pp'}^{(k)} \equiv \left[\exp(\kappa^{(k)})\right]_{pp'} \tag{13}$$

This spin-restricted orbital rotation is expressed as quantum circuits using a fabric of Givens rotations.[S20]

The two-particle operator is defined as

$$\hat{T}^{(k)} \equiv \sum_{p=0}^{M-1} \sum_{\substack{p'=p \mod 2 \\ p+=2}}^{M-2} \tau_{pp'}^{(k)} [(p'+1)^{\dagger} \overline{(p'+1)}^{\dagger} p' \bar{p}' - p'^{\dagger} \bar{p}'^{\dagger} (p'+1) \overline{(p'+1)}] \tag{14}$$

The uCJ implementation is similar but not exact to Refs.[S18] and.[S21] This it is denoted as k-muCJ for clarity, with the 'm' standing for modified. It is important to point out that the choice of VQE ansatz is largely irrelevant from a SAPT perspective and is not a major point in this paper. An example of one layer of the muCJ circuit ansatz is given in Fig. S1.

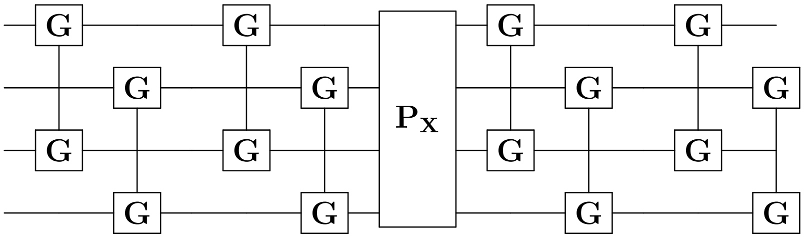

Figure S1: Quantum circuit of a single layer ($k = 1$) k-muCJ VQE entangler circuit for $M = 2$ spatial orbitals or $N = 4$ qubits. Even (odd) qubits represent α (β) spin-orbitals. The quantum circuit starts with two-qubit Givens rotation among α and β orbitals. The next steps are a double substitution operator (four qubit exchange gate) and another layer of Givens rotations.

Using the ansatz discussed above, the VQE objective function is defined as

$$E_{\text{VQE}}(\kappa_{pq}^{k}, \tau_{pq}^{k}) \equiv \langle \Psi_{\text{VQE}}(\kappa_{pq}^{k}, \tau_{pq}^{k}) | \hat{H} | \Psi_{\text{VQE}}(\kappa_{pq}^{k}, \tau_{pq}^{k}) \rangle \tag{15}$$

$$= \langle \Phi_{\text{I}} | \hat{U}^{\dagger}(\kappa_{pq}^{k}, \tau_{pq}^{k}) \hat{H} \hat{U}(\kappa_{pq}^{k}, \tau_{pq}^{k}) | \Phi_{\text{I}} \rangle. \tag{16}$$

The number of measurements scales $\mathcal{O}(N_a^4)$; however, we point out that efficient estimation of the density matrices are currently intensively investigated by several research groups, e.g. Ref. S22 or Ref. S23.

2 Derivation of the Second Order SAPT Terms

It is helpful to first recall standard Rayleigh-Schrödinger perturbation theory for the intermolecular interaction energy. To begin, we can write the Hamiltonian of the combined system as

$$\hat{H} = \hat{H}_A + \hat{H}_B + \hat{V}, \tag{17}$$

where we assume $\hat{H}_X|\Psi_X\rangle = E_X|\Psi_X\rangle$, where $|\Psi_X\rangle$ is the ground state wavefunction of monomer X and $\hat{V}$ contains only the Coulomb interactions between monomer A and B:

$$\hat{V} = \sum_{i=1}^{N_A}\sum_{j=1}^{N_B}\frac{1}{|\mathbf{r}_i - \mathbf{r}_j|} - \sum_{i=1}^{N_A}\sum_{\beta}\frac{Z_\beta}{|\mathbf{R}_\beta - \mathbf{r}_i|} - \sum_{j}^{N_B}\sum_{\alpha}\frac{Z_\alpha}{|\mathbf{R}_\alpha - \mathbf{r}_j|} + \sum_{\alpha}\sum_{\beta}\frac{Z_\alpha Z_\beta}{|\mathbf{R}_\alpha - \mathbf{R}_\beta|}, \tag{18}$$

where it is understood that i and j are distinct indices and the sum over α/β runs over the number of atoms in monomer A/B respectively. Under the assumption that the potential $\hat{V}$ is a small perturbation (valid for weak intermolecular interactions), we can, in principle, now use perturbation theory. Following Ref. S1, one typically starts SAPT by first constructing a perturbation series for the so-called polarization energy

$$|\Psi_{\mathrm{pol}}^{(n)}\rangle = -\hat{R}_0\hat{V}|\Psi_{\mathrm{pol}}^{(n-1)}\rangle + \sum_{k}^{n-1} E_{\mathrm{pol}}^{(k)}\hat{R}_0|\Psi_{\mathrm{pol}}^{(n-k)}\rangle, \tag{19}$$

where

$$\hat{R}_0 = (\hat{H}_0 - E_0 + \hat{P}_0)^{-1}\hat{Q}_0, \tag{20}$$

is the resolvent operator, $\hat{H}_0 = \hat{H}_A + \hat{H}_B$, $\hat{P} = |\Psi_0\rangle\langle\Psi_0|$, $\hat{Q} = 1 - \hat{P}$ and $|\Psi_{\mathrm{pol}}^0\rangle = |\Psi_0\rangle = |\Psi_A^0\Psi_B^0\rangle$. Here we have that $\hat{H} = \hat{H}_A + \hat{H}_B + \hat{V}$ and we assume $\hat{H}_0|\Psi_0\rangle = (E_A^0 + E_B^0)|\Psi_0\rangle$.

The first order polarization energy (usually called the electrostatic energy E_{elst}) is then given by

$$E^{(1)}_{\text{pol}} = \langle \Psi_0 | \hat{V} | \Psi_0 \rangle \tag{21}$$

$$= \bar{\gamma}_{pp'} v^{p'q'}_{pq} \bar{\gamma}_{qq'}, \tag{22}$$

where we used the definition $E^{(n)}_{\text{pol}} = \langle \Psi_0 | \hat{V} | \Psi^{(n)}_{\text{pol}} \rangle$

For the second order polarisation energy, it is conventional in SAPT to split into the induction and dispersion energy, i.e., $E^{(2)}_{\text{pol}} = E_{\text{ind}} + E_{\text{disp}}$. The distinction arises by considering that the induction term accounts for contributions from terms where one monomer is in an excited state, and the dispersion term considers contributions from terms where both monomers are in excited states. For the induction energy we have $|\Psi^{(\text{ind})}_A\rangle \equiv \hat{R}^A_0 \hat{\Omega}^B |\Psi^0_A\rangle$, where $\hat{R}^A_0 = (\hat{H}_A - E^A_0 + \hat{P}_A)^{-1} \hat{Q}_A$ and

$$\hat{\Omega}^{\text{B}} = \sum_i^{N_A} \left(\hat{V}_B(\mathbf{r}_i) + \int d\mathbf{r}_j \frac{\hat{\rho}_B(\mathbf{r}_j)}{|\mathbf{r}_i - \mathbf{r}_j|} \right) \tag{23}$$

is the effective electrostatic field of monomer B. Using the sum over states formula for the resolvent we have then that

$$|\Psi^{(\text{ind})}_A\rangle \equiv \hat{R}^A_0 \hat{\Omega}^B |\Psi^0_A\rangle \tag{24}$$

$$= -\sum_\mu \frac{|\Psi^\mu_A\rangle \langle \Psi^\mu_A | \hat{\Omega}^B | \Psi^0_A \rangle}{E^\mu_A - E^0_A}, \tag{25}$$

from which it follows

$$E_{\text{ind}}(A \leftarrow B) = -\sum_\mu \frac{|\langle \Psi^\mu_A | \hat{\Omega}^B | \Psi^0_A \rangle|^2}{E^\mu_A - E^0_A}, \tag{26}$$

with a similar expression for $E_{\text{ind}}(B \leftarrow A)$. By introducing the (spin-summed) transition

one-particle reduced density matrix

$$\bar{\gamma}^{\mu}_{pp'} = \langle\Psi^0|p^\dagger p'|\Psi^\mu\rangle + \langle\Psi^0|\bar{p}^\dagger\bar{p}'|\Psi^\mu\rangle \tag{27}$$

we can write Eq. (26) as

$$E_{\text{ind}}(A \leftarrow B) = -\sum_{\mu} \frac{(\bar{\gamma}^{\mu}_{pp'}\Omega_{pp'})^2}{E^{\mu}_A - E^0_A}. \tag{28}$$

Similar expressions exist for monomer B.

For the dispersion contribution, we first define

$$|\Psi^{\text{disp}}_{AB}\rangle = -\frac{|\Psi^\mu_A\Psi^\nu_B\rangle\langle\Psi^\mu_A\Psi^\nu_B|\hat{V}_{ee}|\Psi^0_A\Psi^0_B\rangle}{E^\mu_A - E^0_A + E^\nu_B - E^0_B}, \tag{29}$$

so that

$$E^{(2)}_{\text{disp}} = -\sum_{\mu\nu} \frac{|\langle\Psi^0_A\Psi^0_B|\hat{V}_{ee}|\Psi^\mu_A\Psi^\nu_B\rangle|^2}{E^\mu_A - E^0_A + E^\nu_B - E^0_B} \tag{30}$$

$$= -\sum_{\mu\nu} \frac{\left(\bar{\gamma}^{\mu}_{pp'} v^{p'q'}_{pq} \bar{\gamma}^{\nu}_{qq'}\right)^2}{E^\mu_A - E^0_A + E^\nu_B - E^0_B} \tag{31}$$

Equations (22), (28) and (31) provide all polarization energy contributions up to the second order in the intermolecular interaction energy. Unfortunately, without further modification, one finds the RS perturbation theory does not converge for many-electron systems[S1]. Symmetry adapted perturbation theory (SAPT) attempts to fix this issue by explicitly accounting for fermionic anti-symmetry in the wavefunction when electrons undergo exchange processes between monomers.

The SAPT expression for the intermolecular interaction energy is given by[S1]

$$E^{(n)}_{\text{SAPT}} = \frac{\langle\Psi_0|\hat{V}|\mathcal{A}\Psi^{(n-1)}_{\text{pol}}\rangle - \sum_{k=1}^{n-1} E^{(k)}_{\text{SAPT}}\langle\Psi_0|\mathcal{A}\Psi^{(n-k)}_{\text{pol}}\rangle}{\langle\Psi_0|\mathcal{A}\Psi_0\rangle}, \tag{32}$$

where $|\Psi^{(n)}_{\text{pol}}\rangle$ are the nth-order polarization wavefunctions given previously and $\mathcal{A}$ is the

antisymmetrizer operator. With this definition, the SAPT interaction takes the following form

$$E^{(n)}_{\text{SAPT}} = E^{(n)}_{\text{pol}} + E^{(n)}_{\text{exch}}. \tag{33}$$

If one neglects all electron exchange processes other than those that exchange a single electron pair between monomer A and B one arrives at the so-called S^2 approximation to the exchange energies (through second order)[S8,S24–S26]:

$$E^{(1)}_{\text{exch}} = \langle \Psi_0 | (\hat{V} - \bar{V})(\hat{P} - \bar{P}) | \Psi_0 \rangle \tag{34}$$

$$E^{(2)}_{\text{exch-ind}}(A \leftarrow B) = \langle \Psi^0_A \Psi^0_B | (\hat{V} - \bar{V})(\hat{P} - \bar{P}) | \Psi^{\text{ind}}_A \Psi^0_B \rangle, \tag{35}$$

$$E^{(2)}_{\text{exch-disp}} = \langle \Psi^0_A \Psi^0_B | (\hat{V} - \bar{V})(\hat{P} - \bar{P}) | \Psi^{\text{disp}}_{AB} \rangle, \tag{36}$$

where $|\Psi^{\text{ind}}\rangle$ and $|\Psi^{\text{disp}}_{AB}\rangle$ have been defined previously and $\hat{P}$ is defined as the electron exchange operator

$$\hat{P} = -\sum_{i}^{N_A} \sum_{j}^{N_B} \hat{P}_{ij} \tag{37}$$

where $\hat{P}_{ij}$ exchanges an electron from monomer A to monomer B. Note a similar expression exists for $E_{\text{exch-ind}}(A \rightarrow B)$.

It is helpful to note that Eqs. (34) to (36) all share a similar structure. Let us first example Eq. (34):

$$E_{\text{exch}} = \langle \Psi^0_A \Psi^0_B | (\hat{V} - \bar{V}) \hat{P} | \Psi^0_A \Psi^0_B \rangle \tag{38}$$

which can be written in the density matrix formalism of SAPT as

$$E^{(1)}_{\text{exch}}(S^2) = \int \gamma_{\text{int}}(\mathbf{x}_i, \mathbf{x}_j) \left(\tilde{v}(\mathbf{r}_i, \mathbf{r}_j) - \frac{E^{(1)}_{\text{pol}}}{N_A N_B} \right) d\mathbf{x}_i d\mathbf{x}_j, \tag{39}$$

where $\mathbf{x}_i$ and $\mathbf{x}_j$ denote both spin and spatial coordinates of electrons in monomer A and B

respectively, and the 'interaction' density matrix is defined as

$$
\begin{aligned}
&\gamma_{\text{int}}(\mathbf{x}_i,\mathbf{x}_j) = \\
&-\gamma_A(\mathbf{x}_i,\mathbf{x}_j)\gamma_B(\mathbf{x}_j,\mathbf{x}_i) \\
&-\int \gamma_A(\mathbf{x}_i,\mathbf{x}_{j'})\Gamma_B(\mathbf{x}_j,\mathbf{x}_{j'},\mathbf{x}_j,\mathbf{x}_i)d\mathbf{x}_{j'} \\
&-\int \Gamma_A(\mathbf{x}_i,\mathbf{x}_{i'},\mathbf{x}_i,\mathbf{x}_j)\gamma_B(\mathbf{x}_j,\mathbf{x}_{i'})d\mathbf{x}_{i'} \\
&-\int\int \Gamma_A(\mathbf{x}_i,\mathbf{x}_{i'},\mathbf{x}_i,\mathbf{x}_{j'})\Gamma_B(\mathbf{x}_j,\mathbf{x}_{j'},\mathbf{x}_j,\mathbf{x}_{i'})d\mathbf{x}_{i'}d\mathbf{x}_{j'}.
\end{aligned} \tag{40}
$$

By inspection Eq. (35) is *identical* to Eq. (38) up to the replacement of $|\Psi_A^0\rangle$ by the excited state wavefunction $|\Psi_A^\mu\rangle$. Thus we have

$$
\langle\Psi_A^0\Psi_B^0|(\hat{V}-\bar{V})\hat{P}|\Psi_\mu^A\Psi_B^0\rangle = \int \gamma_{\text{int}}^\mu(\mathbf{x}_i,\mathbf{x}_j)\left(\tilde{v}(\mathbf{r}_i,\mathbf{r}_j) - \frac{E_{\text{pol}}^{(1)}}{N_A N_B}\right)d\mathbf{x}_i d\mathbf{x}_j, \tag{41}
$$

where

$$
\begin{aligned}
&\gamma_{\text{int}}^\mu(\mathbf{x}_i,\mathbf{x}_j) = \\
&-\gamma_A^\mu(\mathbf{x}_i,\mathbf{x}_j)\gamma_B(\mathbf{x}_j,\mathbf{x}_i) \\
&-\int \gamma_A^\mu(\mathbf{x}_i,\mathbf{x}_{j'})\Gamma_B(\mathbf{x}_j,\mathbf{x}_{j'},\mathbf{x}_j,\mathbf{x}_i)d\mathbf{x}_{j'} \\
&-\int \Gamma_A^\mu(\mathbf{x}_i,\mathbf{x}_{i'},\mathbf{x}_i,\mathbf{x}_j)\gamma_B(\mathbf{x}_j,\mathbf{x}_{i'})d\mathbf{x}_{i'} \\
&-\int\int \Gamma_A^\mu(\mathbf{x}_i,\mathbf{x}_{i'},\mathbf{x}_i,\mathbf{x}_{j'})\Gamma_B(\mathbf{x}_j,\mathbf{x}_{j'},\mathbf{x}_j,\mathbf{x}_{i'})d\mathbf{x}_{i'}d\mathbf{x}_{j'}.
\end{aligned} \tag{42}
$$

where γ^μ and Γ^μ are transition one- and two-particle density matrices. Expanding the

transition density matrix in orbital space and performing spin summations, we find

$$\langle\Psi_A^0\Psi_B^0|(\hat{V}-\bar{V})\hat{P}|\Psi_A^\mu\Psi_B^0\rangle = -\frac{1}{2}(\bar{\gamma}^\mu_{pp'}\bar{\gamma}_{qq'}\tilde{v}^{q'p'}_{pq} \tag{43}$$

$$\begin{aligned} &+ \bar{\gamma}^\mu_{pp'}\bar{\Gamma}^{qq'}_{q''q'''}S_{p'q'}\tilde{v}^{q'''q''}_{pq} \\ &+ \bar{\gamma}_{qq'}[\bar{\Gamma}^\mu]^{pp'}_{p''p'''}S_{q'p'}\tilde{v}^{p''p'''}_{pq} \\ &+ [\bar{\Gamma}^\mu]^{pp'}_{p''p'''}\bar{\Gamma}^{qq'}_{q''q'''}S_{p'q'''}S_{q'p'''}\tilde{v}^{p''q''}_{pq} \\ &- E^{(1)}_{\text{pol}}S_{pq'}\bar{\gamma}_{qq'}S_{qp'}\bar{\gamma}^\mu_{pp'}). \end{aligned} \tag{44}$$

(45)

It is helpful to define the exchange of functional

(46)

$$F^{\mu\nu} \equiv F[\bar{\gamma}^\mu,\bar{\Gamma}^\mu,\bar{\gamma}^\nu,\bar{\Gamma}^\nu] = -\frac{1}{2}([\bar{\gamma}^\mu]_{pp'}[\bar{\gamma}^\nu]_{qq'}\tilde{v}^{q'p'}_{pq} \tag{47}$$

$$\begin{aligned} &+ [\bar{\gamma}^\mu]_{pp'}[\bar{\Gamma}^\nu]^{qq'}_{q''q'''}S_{p'q'}\tilde{v}^{q'''q''}_{pq} \\ &+ [\bar{\gamma}^\nu]_{qq'}[\bar{\Gamma}^\mu]^{pp'}_{p''p'''}S_{q'p'}\tilde{v}^{p''p'''}_{pq} \\ &+ [\bar{\Gamma}^\mu]^{pp'}_{p''p'''}[\bar{\Gamma}^\nu]^{qq'}_{q''q'''}S_{p'q'''}S_{q'p'''}\tilde{v}^{p''q''}_{pq} \\ &- E^{(1)}_{\text{pol}}S_{pq'}[\bar{\gamma}^\nu]_{qq'}S_{qp'}[\bar{\gamma}^\mu]^{pp'}), \end{aligned} \tag{48}$$

(49)

with the understanding that $\gamma^0 = \gamma$ etc., so that for the exchange-dispersion contribution, we have

$$\langle\Psi_A^0\Psi_B^0|(\hat{V}-\bar{V})\hat{P}|\Psi_A^\mu\Psi_B^\nu\rangle = F^{\mu\nu} = F[\bar{\gamma}^\mu_A,\bar{\Gamma}^\mu_A,\bar{\gamma}^\nu_B,\bar{\Gamma}^\nu_B], \tag{50}$$

which follows by inspection.

With these notational conventions, we can evaluate the exchange-induction term we have

(in the S^2 approximation)

$$E_{\text{exch-ind}}(A \to B) = \langle \Psi_A^0 \Psi_B^0 | (\hat{V} - \bar{V})(\hat{P} - \bar{P}) | \Psi_A^{(\text{ind})} \Psi_B^0 \rangle. \tag{51}$$

Inserting Eq. (25) into Eq. (51) and breaking the expectation value into two terms, we find (dropping $B \to A$ notation)

$$E_{\text{exch-ind}} = -\sum_{\mu} \frac{\langle \Psi_A^0 \Psi_B^0 | (\hat{V} - \bar{V})(\hat{P} - \bar{P}) | \Psi_A^{\mu} \Psi_B^0 \rangle \langle \Psi_A^{\mu} \Psi_B^0 | \hat{\Omega}^B | \Psi_A^0 \Psi_B^0 \rangle}{E_A^{\mu} - E_A^0}. \tag{52}$$

Let us first look at the term in the numerator, $\langle \Psi_A^0 \Psi_B^0 | (\hat{V} - \bar{V})(\hat{P} - \bar{P}) | \Psi_A^{\mu} \Psi_B^0 \rangle$ which we will break down into two pieces:

$$\langle \Psi_A^0 \Psi_B^0 | (\hat{V} - \bar{V}) \hat{P} | \Psi_A^{\mu} \Psi_B^0 \rangle \tag{53}$$

and

$$\bar{P} \langle \Psi_A^0 \Psi_B^0 | (\hat{V} - \bar{V}) | \Psi_A^{\mu} \Psi_B^0 \rangle. \tag{54}$$

Eq. (53) is given by F^{μ} as discussed previously while Eq. (54) is simply (note $\langle \Psi_A^0 | \Psi_A^{\mu} \rangle = 0$ so the $\bar{P}\bar{V}$ contribution is zero)

$$\bar{P} \langle \Psi_A^0 \Psi_B^0 | \hat{V} | \Psi_A^{\mu} \Psi_B^0 \rangle = \bar{P} \langle \Psi_A^0 \Psi_B^0 | \hat{\Omega} | \Psi_A^{\mu} \Psi_B^0 \rangle \tag{55}$$

$$= \bar{P} \bar{\gamma}_{pp'}^{\mu} \Omega_{pp'}^B \tag{56}$$

and by inspection with Eq. (39)

$$\bar{P} \equiv \langle \Psi_A^0 \Psi_B^0 | \hat{P} | \Psi_A^0 \Psi_B^0 \rangle = \frac{1}{N_A N_B} \int dx \; dx' \; \gamma_{\text{int}}(\mathbf{x}, \mathbf{x}') \tag{57}$$

$$= -\frac{1}{2} S_{pq'} \bar{\gamma}_{qq'} S_{qp'} \bar{\gamma}_{pp'}. \tag{58}$$

So, putting all of this together, we have

$$E_{\text{exch-ind}}(B \to A) = -\left(\frac{F^\mu t^\mu}{\omega_A^\mu} + E_{\text{ind}}(B \to A)\bar{P} \right), \tag{59}$$

where

$$t^\mu = \gamma^\mu_{pp'} \Omega^B_{pp'}, \tag{60}$$

and we have used the fact that

$$\frac{(\bar{\gamma}^\mu_{pp'} \Omega^B_{pp'})^2}{\omega_A^\mu} \equiv -E_{\text{ind}}(B \to A). \tag{61}$$

with

$$\omega_A^\mu = E_A^\mu - E_A^0 \tag{62}$$

and The full expression is then

$$E_{\text{exch-ind}} = E_{\text{exch-ind}}(A \to B) + E_{\text{exch-ind}}(B \to A) = -\left(\frac{F^\mu t^\mu}{\omega_A^\mu} + \frac{F^\nu t^\nu}{\omega_B^\nu} + E_{\text{ind}}\bar{P} \right). \tag{63}$$

The second-order exchange-dispersion energy can be found in a similar fashion. We can start from the definition in the S^2 approximation

$$E_{\text{exch-disp}}(S^2) = \langle \Psi_A^0 \Psi_B^0 | (\hat{V} - \bar{V})(\hat{P} - \bar{P}) | \Psi_{AB}^{\text{disp}} \rangle, \tag{64}$$

In contrast to the induction term, the dispersion interaction includes terms where both monomers are in an excited state. Much like the exchange-induction interaction, we can split Eq. (64) into two terms. Inserting Eq. (29) into Eq. (64) we first have to evaluate

$$\langle \Psi_A^0 \Psi_B^0 | (\hat{V} - \bar{V}) \hat{P} | \Psi_A^\mu \Psi_B^\nu \rangle = F^{\mu\nu} = F[\bar{\gamma}_A^\mu, \bar{\Gamma}_A^\mu, \bar{\gamma}_B^\nu, \bar{\Gamma}_B^\nu], \tag{65}$$

which follows by inspection. We also identify the usual induction numerator term

$$\langle \Psi_A^0 \Psi_B^0 | V_{ee} | \Psi_A^\mu \Psi_B^\nu \rangle = \upsilon_{pq}^{p'q'} \gamma_{pp'}^\mu \gamma_{qq'}^\nu = t^{\mu\nu}. \tag{66}$$

Noting that

$$E_{\text{disp}}^{(2)} = -\sum_{\mu\nu} \frac{(t^{\mu\nu})^2}{\omega_A^\mu + \omega_B^\nu} \tag{67}$$

and combining all of these with Eq. (58) we find

$$E_{\text{exch-disp}} = -\left(\frac{F^{\mu\nu} t^{\mu\nu}}{\omega_A^\mu + \omega_B^\nu} + E_{\text{disp}} \bar{P} \right). \tag{68}$$

3 Extended Random Phase Approximation

Evaluating the SAPT expressions given in Section 2 requires a knowledge of the one- and two-particle transition density matrices at a given level of theory. There are two challenges to achieving this given current NISQ hardware. First is that we can only simulate a small fragment of the problem in a quantum computer, which is achieved here using an active space approach. Second is that, in principle, we would need to compute all eigenvalues and excited states to compute the transition density matrices, which is significantly more challenging than just computing the ground state energy.

To overcome these issues, we instead include only the subset of particle-hole excitations. In particular, we follow Hapka *et al.*[S8,S26,S27] and use the extended random phase approximation[S13,S28,S29] (ERPA) to approximately determined excited state properties from CASSCF quality wavefunctions. For notational clarity, in this section, we will not distinguish between monomers, and orbitals are general spatial orbitals.

Following Pernal[S13,S29], in the ERPA solves the following eigenvalue problem

$$\begin{bmatrix} A & B \\ B & A \end{bmatrix} \begin{bmatrix} X_\nu \\ Y_\nu \end{bmatrix} = \omega_\nu \begin{bmatrix} -N & 0 \\ 0 & N \end{bmatrix} \begin{bmatrix} X_\nu \\ Y_\nu \end{bmatrix}. \tag{69}$$

where

$$A_{pq,rs} = \langle \Psi_0 | [p^\dagger q, [\hat{H}, s^\dagger r] | \Psi_0 \rangle \tag{70}$$

$$B_{pq,rs} = \langle \Psi_0 | [p^\dagger q, [\hat{H}, r^\dagger s] | \Psi_0 \rangle \tag{71}$$

$$N_{pq,rs} = \langle \Psi_0 | [p^\dagger q, s^\dagger r] | \Psi_0 \rangle = \delta_{ps}\delta_{qr}(n_q - n_p), \tag{72}$$

where the above equations are valid in the natural spin-orbital basis corresponding to $|\Psi_0\rangle$, and we take $p > q$ and $r > s$. Computing the Hessian matrices in Eq. (69) is somewhat tedious, but expressions are available in the literature[S13,S29].

Note that using excited states from the ERPA introduces two approximations in the quality of the resulting SAPT interaction energies. The first approximation is that even with an exact FCI wavefunction, the ERPA will not yield all the excited states of the Hamiltonian as only particle-hole-like excitations are included. The second issue is that the CASSCF wavefunction is not an eigenstate of the zeroth order Hamiltonian in the SAPT perturbation theory. In principle, this last problem can be addressed within the context of the adiabatic-connection formalism based upon the ERPA[S8,S26,S27,S30,S31] which yields the so-called coupled approximation used in this work. This amounts to using the full Hamiltonian in Eq. (69) with the CASSCF (or VQE) wavefunction as $|\Psi_0\rangle$. The adiabatic-connection approach assumes the one- and two-particle reduced density matrices remain constant across the adiabatic-connection pathway. This approximation appears to perform well in the sense that SAPT(CASSCF) compares favourably to FCI interaction energies[S8,S26,S27].

3.1 Spin-Summation

In this work, we only target singlet excited states and thus $X_{\beta\alpha} = X_{\alpha\beta} = 0$; therefore, we explicitly form the spin-restricted ERPA equations. In particular, we form

$$A_{pq,rs} = \langle\Psi_0|\hat{E}_{pq}, [\hat{H}, \hat{E}_{sr}]|\Psi_0\rangle \tag{73}$$

$$B_{pq,rs} = \langle\Psi_0|[\hat{E}_{pq}, [\hat{H}, \hat{E}_{rs}]|\Psi_0\rangle \tag{74}$$

$$N_{pq,rs} = \langle\Psi_0|[\hat{E}_{pq}, \hat{E}_{sr}]|\Psi_0\rangle = \delta_{ps}\delta_{qr}(n_q - n_p), \tag{75}$$

where $\hat{E}_{rs} = (r^\dagger s + \bar{r}^\dagger \bar{s})$. It is helpful to note the following identities:

$$[AB, C] = A\{B, C\} - \{A, C\}B \tag{76}$$

$$[AB, CD] = [AB, C]D + C[AB, D] \tag{77}$$

So, for example,

$$\begin{aligned}[\hat{E}_{rs}, \hat{E}_{qp}] &= [r^\dagger s + \bar{r}^\dagger \bar{s}, q^\dagger p + \bar{q}^\dagger \bar{p}] \\ &= [r^\dagger s, q^\dagger p] + [r^\dagger s, \bar{q}^\dagger \bar{p}] + [\bar{r}^\dagger \bar{s}, q^\dagger p] + [\bar{r}^\dagger \bar{s}, \bar{q}^\dagger \bar{p}],\end{aligned} \tag{78}$$

and

$$[r^\dagger s, q^\dagger p] = [r^\dagger s, q^\dagger]p + q^\dagger[r^\dagger s, p] \tag{79}$$

$$= (r^\dagger\{s, q^\dagger\} - \{r^\dagger, q^\dagger\}s)p + q^\dagger(r^\dagger\{s, p\} - \{r^\dagger, p\}s) \tag{80}$$

$$= r^\dagger p\delta_{sq} - q^\dagger s\delta_{rp} \tag{81}$$

$$[\bar{r}^\dagger \bar{s}, \bar{q}^\dagger \bar{p}] = \bar{r}^\dagger \bar{p}\delta_{\bar{s}\bar{q}} - \bar{q}^\dagger \bar{s}\delta_{\bar{r}\bar{p}} \tag{82}$$

$$[\bar{r}^\dagger \bar{s}, q^\dagger p] = [r^\dagger s, \bar{q}^\dagger \bar{p}] = 0 \tag{83}$$

Similarly[S32],

$$[\hat{E}_{mn}, \hat{E}_{pq}] = \hat{E}_{mq}\delta_{pn} - \hat{E}_{pn}\delta_{mq} \tag{84}$$

$$[\hat{E}_{rs}, [\hat{E}_{ij}, \hat{E}_{qp}]] = [\hat{E}_{rs}, \hat{E}_{ip}]\delta_{qj} - [\hat{E}_{rs}, \hat{E}_{qj}]\delta_{ip} \tag{85}$$

$$= \hat{E}_{rp}\delta_{is}\delta_{qj} - \hat{E}_{is}\delta_{rp}\delta_{qj} - \hat{E}_{rj}\delta_{qs}\delta_{ip} + \hat{E}_{qs}\delta_{rj}\delta_{ip} \tag{86}$$

$$[\hat{E}_{mn}, \hat{e}_{pqrs}] = \delta_{pn}\hat{e}_{mqrs} - \delta_{mq}\hat{e}_{pnrs} + \delta_{rn}\hat{e}_{pqms} - \delta_{ms}\hat{e}_{pqrn}, \tag{87}$$

where $\hat{e}_{pqrs} = p^\dagger r^\dagger sq + p^\dagger \bar{r}^\dagger \bar{s}q + \bar{p}^\dagger r^\dagger s\bar{q} + \bar{p}^\dagger \bar{r}^\dagger \bar{s}\bar{q}$. We can now evaluate the commutator on the left hand side of Eq. (73) where we take

$$\hat{H} = \sum_{pq} h_{pq}(p^\dagger q + \bar{p}^\dagger \bar{q}) + \frac{1}{2}\sum_{pqrs}(pq|rs)\hat{e}_{pqrs}. \tag{88}$$

Let us begin with the one-body term (dropping hats on operators for brevity, and here all orbital labels refer to general spatial orbitals, no distinction is made between active, core or inactive)

$$\sum_{ij} h_{ij}[E_{rs}, [E_{ij}, E_{qp}]] = \sum_{ij} h_{ij}(\hat{E}_{rp}\delta_{is}\delta_{qj} - \hat{E}_{is}\delta_{rp}\delta_{qj} - \hat{E}_{rj}\delta_{qs}\delta_{ip} + \hat{E}_{qs}\delta_{rj}\delta_{ip}) \tag{89}$$

$$= h_{sq}\hat{E}_{rp} - \sum_i h_{iq}\hat{E}_{is}\delta_{rp} - \sum_j h_{pj}\hat{E}_{rj}\delta_{qs} + h_{pr}\hat{E}_{qs} \tag{90}$$

If we work in the natural orbital basis $\langle\Psi_0|\hat{E}_{pq}|\Psi_0\rangle = \bar{\gamma}_{pq} = n_p\delta_{pq}$, with $0 \leq n_p \leq 2$[1], then we have

$$\langle\sum_{ij} h_{ij}[E_{rs}, [E_{ij}, E_{qp}]]\rangle = h_{sq}\delta_{rp}(n_r - n_s) + h_{pr}\delta_{qs}(n_q - n_r). \tag{91}$$

[1] Note we assume $n_p < n_q$ for $p > q$ (i.e. the basis is ordered in descending order of natural orbital occupancy) which is the opposite ordering that is usually taken in the ERPA literature[S13,S29]

For the two-body part, we have to evaluate expressions like (see Eq. (74))

$$\sum_{ijkl}(ij|kl)[E_{rs},[e_{ijkl},E_{pq}]] \tag{92}$$

Let us look at

$$[E_{rs},[e_{ijkl},E_{pq}]] = -[E_{rs},[E_{pq},e_{ijkl}]] = -[E_{rs},\delta_{iq}\hat{e}_{pjkl} - \delta_{pj}\hat{e}_{iqkl} + \delta_{kq}\hat{e}_{ijpl} - \delta_{pl}\hat{e}_{ijkq}] \tag{93}$$

$$= -[E_{rs},\delta_{iq}\hat{e}_{pjkl} - \delta_{pj}\hat{e}_{iqkl} + \delta_{kq}\hat{e}_{ijpl} - \delta_{pl}\hat{e}_{ijkq}] \tag{94}$$

$$[\hat{E}_{rs},\delta_{iq}\hat{e}_{pjkl}] = \delta_{iq}(\delta_{ps}\hat{e}_{rjkl} - \delta_{rj}\hat{e}_{pskl} + \delta_{ks}\hat{e}_{pjrl} - \delta_{rl}\hat{e}_{pjks}) \tag{95}$$

$$[\hat{E}_{rs},\delta_{pj}\hat{e}_{iqkl}] = \delta_{pj}(\delta_{is}\hat{e}_{rqkl} - \delta_{rq}\hat{e}_{iskl} + \delta_{ks}\hat{e}_{iqrl} - \delta_{rl}\hat{e}_{iqks}) \tag{96}$$

$$[\hat{E}_{rs},\delta_{kq}\hat{e}_{ijpl}] = \delta_{kq}(\delta_{is}\hat{e}_{rjpl} - \delta_{rj}\hat{e}_{ispl} + \delta_{ps}\hat{e}_{ijrl} - \delta_{rl}\hat{e}_{ijps}) \tag{97}$$

$$[\hat{E}_{rs},\delta_{pl}\hat{e}_{ijkq}] = \delta_{pl}(\delta_{is}\hat{e}_{rjkq} - \delta_{rj}\hat{e}_{iskq} + \delta_{ks}\hat{e}_{ijrq} - \delta_{rq}\hat{e}_{ijks}) \tag{98}$$

so,

$$\begin{aligned}\langle\sum_{ijkl}(ij|kl)[E_{rs},[e_{ijkl},E_{pq}]]\rangle = -\sum_{ijkl}(ij|kl)\Big[&\delta_{iq}(\delta_{ps}\bar{\Gamma}_{rjkl} - \delta_{rj}\bar{\Gamma}_{pskl} + \delta_{ks}\bar{\Gamma}_{pjrl} - \delta_{rl}\bar{\Gamma}_{pjks}) \\ &- \delta_{pj}(\delta_{is}\bar{\Gamma}_{rqkl} - \delta_{rq}\bar{\Gamma}_{iskl} + \delta_{ks}\bar{\Gamma}_{iqrl} - \delta_{rl}\bar{\Gamma}_{iqks}) \\ &+ \delta_{kq}(\delta_{is}\bar{\Gamma}_{rjpl} - \delta_{rj}\bar{\Gamma}_{ispl} + \delta_{ps}\bar{\Gamma}_{ijrl} - \delta_{rl}\bar{\Gamma}_{ijps}) \\ &- \delta_{pl}(\delta_{is}\bar{\Gamma}_{rjkq} - \delta_{rj}\bar{\Gamma}_{iskq} + \delta_{ks}\bar{\Gamma}_{ijrq} - \delta_{rq}\bar{\Gamma}_{ijks})\Big],\end{aligned} \tag{99}$$

where the spin-summed two-particle reduced density matrix is

$$\bar{\Gamma}_{pqrs} = \langle\Psi_0|\hat{e}_{pqrs}|\Psi_0\rangle. \tag{100}$$

Removing the Kroenecker deltas we find

$$\frac{1}{2}\langle\sum_{ijkl}(ij|kl)[E_{rs},[e_{ijkl},E_{pq}]]\rangle =$$
$$-\frac{1}{2}\Big[\sum_{jkl}(qj|kl)\delta_{ps}\bar{\Gamma}_{rjkl}-\sum_{kl}(qr|kl)\bar{\Gamma}_{pskl}+\sum_{jl}(qj|sl)\bar{\Gamma}_{pjrl}-\sum_{jk}(qj|kr)\bar{\Gamma}_{pjks}$$
$$-\sum_{kl}(sp|kl)\bar{\Gamma}_{rqkl}+\sum_{ikl}(ip|kl)\delta_{rq}\bar{\Gamma}_{iskl}-\sum_{il}(ip|sl)\bar{\Gamma}_{iqrl}+\sum_{ik}(ip|kr)\bar{\Gamma}_{iqks}$$
$$+\sum_{jl}(sj|ql)\bar{\Gamma}_{rjpl}-\sum_{il}(ir|ql)\bar{\Gamma}_{ispl}+\sum_{ijl}(ij|ql)\delta_{ps}\bar{\Gamma}_{ijrl}-\sum_{ij}(ij|qr)\bar{\Gamma}_{ijps}$$
$$-\sum_{jk}(sj|kp)\bar{\Gamma}_{rjkq}+\sum_{ik}(ir|kp)\bar{\Gamma}_{iskq}-\sum_{ij}(ij|sp)\bar{\Gamma}_{ijrq}+\sum_{ijk}(ij|kp)\delta_{rq}\bar{\Gamma}_{ijks}\Big] \tag{101}$$

which can be simplified using symmetries in the ERIs and TPDMs. Note Eq. (101) reduces to the expression given in Ref. S13 if working in the natural spin orbital basis.

3.2 Numerical Solution

With the expressions for the Hessians at hand we can now solve Eq. (69). First, it is helpful to bring this expression into a symmetric eigenvalue problem of half the dimension. This has two advantages, namely the computational cost will be reduced by a factor of eight and we can use a symmetric eigenvalue solver.

To proceed it is important to note some features of Eq. (69). First, we choose the normalization:

$$Y_\nu^\dagger N Y_\nu - X_\nu^\dagger N X_\nu = 1, \tag{102}$$

$$(Y-X)_\nu^\dagger N (Y+X)_\nu = 1. \tag{103}$$

Next, we note that

$$(A+B)(X+Y)_\nu = \omega_\nu N(Y-X)_\nu \tag{104}$$

$$(A-B)(Y-X)_\nu = \omega_\nu N(X+Y)_\nu, \tag{105}$$

which implies

$$(A+B)(X+Y)_\nu = \omega_\nu^2 N(A-B)^{-1} N(X+Y)_\nu \tag{106}$$

or

$$(A+B)N^{-1/2}N^{1/2}(X+Y)_\nu = \omega_\nu^2 N^{1/2}N^{1/2}(A-B)^{-1}N^{1/2}N^{1/2}(X+Y)_\nu \tag{107}$$

$$A_+ T_\nu = \omega_\nu^2 A_-^{-1} T_\nu \tag{108}$$

$$A_- A_+ T_\nu = \omega_\nu^2 T_\nu \tag{109}$$

if $(A-B)$ is positive definite, and we have defined

$$A_+ = N^{-1/2}(A+B)N^{-1/2} \tag{110}$$

$$A_- = N^{-1/2}(A-B)N^{-1/2} \tag{111}$$

$$T_\nu = N^{1/2}(X+Y)_\nu. \tag{112}$$

If $(A+B)$ is also positive definite we can write

$$A_+^{1/2}A_+^{1/2}A_-A_+^{1/2}A_+^{1/2}T_\nu = \omega_n^2 A_+ T_\nu \tag{113}$$

$$A_+^{1/2}A_-A_+^{1/2}A_+^{1/2}T_\nu = \omega_n^2 A_+^{1/2} T_\nu \tag{114}$$

$$A_+^{1/2}A_-A_+^{1/2}Q_\nu = \omega_n^2 Q_\nu \tag{115}$$

$$\tag{116}$$

where

$$Q_n = A_+^{1/2} T_\nu = A_+^{1/2} N^{1/2} (X+Y)_\nu, \tag{117}$$

$Q^\dagger Q = 1$, and

$$(X+Y)_\nu = N^{-1/2} A_+^{-1/2} Q_\nu. \tag{118}$$

This allows us to write combinations of the X_ν and Y_ν matrices necessary for building transition density matrices in terms of the solutions of the Hermitian eigenvalue problem. The factors of $N^{-1/2}$ can be determined using canonical orthogonalization with a threshold to discard small eigenvalues.

3.3 Normalization

A useful sanity check at this point is that we have

$$(A+B)(X+Y)_\nu = \omega_\nu N (Y-X)_\nu. \tag{119}$$

Multiplying on the right by $(X+Y)^\dagger$ we have and using Eq. (103)

$$(X+Y)_\nu (A+B)(X+Y)_\nu^\dagger = \omega_\nu (X+Y)_\nu N (Y-X)_\nu^\dagger \tag{120}$$

$$(X+Y)_\nu (A+B)(X+Y)_\nu^\dagger = \omega_\nu \tag{121}$$

Inserting Eq. (118) in Eq. (121) we find

$$(X+Y)_\nu (A+B)(X+Y)_\nu^\dagger = Q_\nu A_+^{-1/2} N^{-1/2} (A+B) N^{-1/2} A_+^{-1/2} Q_\nu = Q_\nu A_+^{-1/2} A_+ A_+^{-1/2} Q_\nu = 1 \neq \omega_\nu! \tag{122}$$

A more consistent normalization of Eq. (118) can be found by

$$(X+Y)_\nu \equiv (T_+)_\nu = \sqrt{\omega_\nu} N^{-1/2} A_+^{-1/2} Q_\nu \tag{123}$$

and by Eq. (103) we can deduce

$$(Y - X)_\nu \equiv (T_-)_\nu = \frac{1}{\sqrt{\omega_\nu}} N^{-1/2} A_+^{1/2} Q_\nu \tag{124}$$

from which it follows that

$$X_\nu = \frac{1}{2}(T_+ - T_-)_\nu \tag{125}$$

$$Y_\nu = \frac{1}{2}(T_+ + T_-)_\nu. \tag{126}$$

To compute the symmetrized (spin summed) transition density matrix we follow Pernal[S13,S33] and use the identity

$$\bar{\gamma}_{pq}^{\nu} = \langle \Psi_0 | \hat{E}_{pq} | \Psi_\nu \rangle = \langle 0 | [\hat{E}_{pq}, \hat{O}_\nu^\dagger] | 0 \rangle, \tag{127}$$

then

$$\gamma_{pq}^{\nu} + \gamma_{qp}^{\nu} = [N(Y - X)]_{pq}^{\nu} \qquad \forall_{p>q}, \tag{128}$$

where

$$\hat{O}_\nu^\dagger = \sum_{p>q} \left(X_{pq}^{\nu} \hat{E}_{pq} + Y_{pq}^{\nu} \hat{E}_{qp} \right) \tag{129}$$

For the transition two-particle reduced density matrix, we have

$$\bar{\Gamma}_{pqrs}^{\nu} = \langle \Psi_0 | [\hat{e}_{pqrs}, \hat{O}_\nu^\dagger] | \Psi_0 \rangle. \tag{130}$$

Using Eq. (87) and looking at the X part of $\hat{O}^\dagger_\nu$ first we have

$$\bar{\Gamma}^{\nu(X)}_{pqrs} = -\sum_{m>n} X^\nu_{mn} \langle [\hat{E}_{mn}, \hat{e}_{pqrs}] \rangle \tag{131}$$

$$= -\sum_{m>n} X^\nu_{mn} \left(\delta_{pn}\bar{\Gamma}_{mqrs} - \delta_{mq}\bar{\Gamma}_{pnrs} + \delta_{rn}\bar{\Gamma}_{pqms} - \delta_{ms}\bar{\Gamma}_{pqrn} \right) \tag{132}$$

and similarly, we have

$$\bar{\Gamma}^{\nu(Y)}_{pqrs} = -\sum_{m>n} Y^\nu_{mn} \langle [\hat{E}_{nm}, \hat{e}_{pqrs}] \rangle \tag{133}$$

$$= -\sum_{m>n} Y^\nu_{mn} \left(\delta_{pm}\bar{\Gamma}_{nqrs} - \delta_{nq}\bar{\Gamma}_{pmrs} + \delta_{rm}\bar{\Gamma}_{pqns} - \delta_{ns}\bar{\Gamma}_{pqrm} \right) \tag{134}$$

and naturally $\bar{\Gamma}^\nu_{pqrs} = \bar{\Gamma}^{\nu(X)}_{pqrs} + \bar{\Gamma}^{\nu(Y)}_{pqrs}$, and we have raised the eigenvalue index for clarity. It is helpful to remove the Kroenecker deltas so that

$$\begin{aligned} \bar{\Gamma}^\nu_{pqrs} = &- \left(\sum_{m=p+1}^{M} X^\nu_{mp}\bar{\Gamma}_{mqrs} + \sum_{m=0}^{p} Y^\nu_{pm}\bar{\Gamma}_{mqrs} \right) \\ &+ \left(\sum_{m=0}^{q} X^\nu_{qm}\bar{\Gamma}_{pmrs} + \sum_{m=q+1}^{M} Y^\nu_{mq}\bar{\Gamma}_{pmrs} \right) \\ &- \left(\sum_{m=r+1}^{M} X^\nu_{mr}\bar{\Gamma}_{pqms} + \sum_{m=0}^{r} Y^\nu_{rm}\bar{\Gamma}_{pqms} \right) \\ &+ \left(\sum_{m=0}^{s} X^\nu_{sm}\bar{\Gamma}_{pqrm} + \sum_{m=s+1}^{M} Y^\nu_{sm}\bar{\Gamma}_{pqrm} \right), \end{aligned} \tag{135}$$

where M is the number of (natural) spatial orbitals. If we define

$$Q^\nu_{pq} = X^\nu_{pq} \ \forall \ p > q \tag{136}$$

$$Q^\nu_{pq} = Y^\nu_{qp} \ \ \forall \ q > p \tag{137}$$

$$R^\nu_{pq} = Y^\nu_{pq} \ \ \forall \ p > q \tag{138}$$

$$R^\nu_{pq} = X^\nu_{qp} \ \forall \ q > p \tag{139}$$

then we have

$$\bar{\Gamma}^{\nu}_{pqrs} = \sum_{m} \left(-Q^{\nu}_{mp}\bar{\Gamma}_{mqrs} + R^{\nu}_{mq}\bar{\Gamma}_{pmrs} - Q^{\nu}_{mr}\bar{\Gamma}_{pqms} + R^{\nu}_{ms}\bar{\Gamma}_{pqrm} \right). \tag{140}$$

A helpful reference point to benchmark is the Hartree–Fock limit $|\Psi_0\rangle \rightarrow |\mathrm{RHF}\rangle$ then

$$\bar{\Gamma}_{pqrs} = \bar{\gamma}_{pq}\bar{\gamma}_{rs} - \frac{1}{2}\bar{\gamma}_{ps}\bar{\gamma}_{rq} \tag{141}$$

$$= n_q\delta_{pq}n_s\delta_{rs} - \frac{1}{2}n_s\delta_{ps}n_q\delta_{rq}, \tag{142}$$

where $n_i = 2$ and $n_a = 0$ for i and occupied MO and a a virtual MO. Let us first insert the Coulomb like terms from Eq. (142) into the first two terms of Eq. (140). We have

$$\sum_{m} -Q^{\nu}_{mp}\bar{\Gamma}_{mqrs} + R^{\nu}_{mq}\bar{\Gamma}_{pmrs} \rightarrow \sum_{m}(R^{\nu}_{mq}n_p\delta_{mp}n_s - Q^{\nu}_{mp}n_q\delta_{mq})n_s\delta_{rs} \tag{143}$$

$$= (R^{\nu}_{pq}n_p - Q^{\nu}_{qp}n_q)n_s\delta_{rs}. \tag{144}$$

Now for $p > q$ we have

$$\sum_{m} -Q^{\nu}_{mp}\bar{\Gamma}_{mqrs} + R^{\nu}_{mq}\bar{\Gamma}_{pmrs} \rightarrow (Y^{\nu}_{pq}n_p - Y^{\nu}_{pq}n_q)n_s\delta_{rs} \tag{145}$$

$$= (n_p - n_q)Y^{\nu}_{pq}n_s\delta_{rs} \tag{146}$$

and for $p < q$ we have

$$\sum_{m} -Q^{\nu}_{mp}\bar{\Gamma}_{mqrs} + R^{\nu}_{mq}\bar{\Gamma}_{pmrs} \rightarrow (X^{\nu}_{qp}n_p - X_{qp}n_q)n_s\delta_{rs} \tag{147}$$

$$= (n_p - n_q)X_{qp}n_s\delta_{rs} \tag{148}$$

$$= -(n_p - n_q)X_{pq}n_s\delta_{rs}\ \forall\ p > q, \tag{149}$$

where we relabelled p and q in the last line. Thus we have

$$\sum_m -Q^{\nu}_{mp}\bar{\Gamma}_{mqrs} + R^{\nu}_{mq}\bar{\Gamma}_{pmrs} \xrightarrow{\text{Coulomb-like}} \gamma^{\nu}_{pq} n_s \delta_{rs}, \tag{150}$$

and by inspection

$$\sum_m -Q^{\nu}_{mr}\bar{\Gamma}_{pqms} + R^{\nu}_{ms}\bar{\Gamma}_{pqrm} \xrightarrow{\text{Coulomb-like}} \gamma^{\nu}_{rs} n_q \delta_{pq}. \tag{151}$$

For the exchange terms we just need to set $q \to s$ so putting it together we find

$$\bar{\Gamma}^{\nu}_{pqrs} = \gamma^{\nu}_{pq} n_s \delta_{rs} + \gamma^{\nu}_{rs} n_p \delta_{pq} - \frac{1}{2}\gamma^{\nu}_{ps} n_q \delta_{rq} - \frac{1}{2}\gamma^{\nu}_{rq} n_s \delta_{ps}. \tag{152}$$

This expression is useful for checking that exchange-induction and exchange-dispersion terms reduce to their appropriate SAPT0 expressions.

3.4 Numerical Implementation of SAPT Expressions Within the ERPA

In principle, one could proceed to evaluate the SAPT expressions in Section 2 by building the transition one- and two-particle density matrices. Indeed, this is possible for the first-order SAPT terms that do not contain any response term, and the second-order polarization energies which are functions of the transition one-particle reduced density matrix only. However, forming the transition two-particle density matrix, which is required for exchange-induction and exchange-induction is not practical in general so some work needs to be done. We follow Refs. S8,S26 and try to optimize the contraction order of the intermediate tensors. Let us first look at the exchange-induction term. The first two terms only contain the one-particle

(transition) density matrix; we start with the derivation of the third term:

$$F^{\mu}_{3a} = -\sum_{m>p} \tilde{Q}^{\mu}_{mp} \bar{\Gamma}^{mp'}_{p''p'''} \bar{\gamma}_{qq'} S_{q'p'} \tilde{v}^{p''p'''}_{pq}. \tag{153}$$

Let

$$T^{mp'}_{pq} = \bar{\Gamma}^{mp'}_{p''p'''} \tilde{v}^{p''p'''}_{pq} \tag{154}$$

and

$$N_{p'q} = \bar{\gamma}_{qq'} S_{q'p'} \tag{155}$$

then

$$W_{pm} = T^{mp'}_{pq} N_{p'q} \tag{156}$$

so

$$F^{\mu}_{3a} = -\sum_{m>p} \tilde{Q}^{\mu}_{mp} W_{pm}. \tag{157}$$

$$F^{\mu}_{3b} = \sum_{m>p''} \tilde{R}^{\mu}_{mp''} \bar{\Gamma}^{pp'}_{mp'''} \bar{\gamma}_{qq'} S_{q'p'} \tilde{v}^{p''p'''}_{pq} \tag{158}$$

$$= \sum_{m>p''} \tilde{R}^{\mu}_{mp''} \left(\bar{\Gamma}^{pp'}_{mp'''} \tilde{v}^{p''p'''}_{pq} \right) \left(\bar{\gamma}_{qq'} S_{q'p'} \right) \tag{159}$$

$$= \sum_{m>p''} \tilde{R}^{\mu}_{mp''} T^{p''p'}_{mq} N_{p'q} \tag{160}$$

$$= \sum_{m>p''} \tilde{R}^{\mu}_{mp''} W_{p''m}. \tag{161}$$

Next we have

$$F^{\mu}_{3c} = -\sum_{m>p'} \tilde{Q}^{\mu}_{mp'} \bar{\Gamma}^{pm}_{p''p'''} \bar{\gamma}_{qq'} S_{q'p'} \tilde{v}^{p''p'''}_{pq} \tag{162}$$

$$= -\sum_{m>p'} \tilde{Q}^{\mu}_{mp'} \left(\bar{\Gamma}^{pm}_{p''p'''} \tilde{v}^{p''p'''}_{pq} \right) \left(\bar{\gamma}_{qq'} S_{q'p'} \right) \tag{163}$$

$$= -\sum_{m>p'} \tilde{Q}^{\mu}_{mp'} T_{mq} N_{p'q} \tag{164}$$

$$= -\sum_{m>p'} \tilde{Q}^{\mu}_{mp'} W_{p'm} \tag{165}$$

and finally

$$F^{\mu}_{3d} = \sum_{m>p'''} \tilde{R}^{\mu}_{mp'''} \bar{\Gamma}^{pp'}_{p'm} \bar{\gamma}_{qq'} S_{q'p'} \tilde{v}^{p''p'''}_{pq} \tag{166}$$

$$= \sum_{m>p'''} \tilde{R}^{\mu}_{mp'''} \left(\bar{\Gamma}^{pp'}_{p''m} \tilde{v}^{p''p'''}_{pq} \right) \left(\bar{\gamma}_{qq'} S_{q'p'} \right) \tag{167}$$

$$= \sum_{m>p'''} \tilde{R}^{\mu}_{mp'''} T^{p'p'''}_{mq} N_{p'q} \tag{168}$$

$$= \sum_{m>p'''} \tilde{R}^{\mu}_{mp'''} W_{p''m}. \tag{169}$$

For the fourth term, we have to evaluate something like

$$F^{\mu}_{4a} = \left(\sum_{m>p} X^{\nu}_{mp} + \sum_{p>m} Y^{\nu}_{pm} \right) \bar{\Gamma}^{mp'}_{p''p'''} \bar{\Gamma}^{qq'}_{q''q'''} S_{p'q'''} S_{q'p'''} \tilde{v}^{p''q''}_{pq} \tag{170}$$

we can define

$$U^{p''q'}_{pp'} = \bar{\Gamma}^{qq'}_{q''q'''} \tilde{v}^{p''q''}_{pq} S_{p'q'''} \tag{171}$$

and

$$M^{mp'}_{p''q'} = \bar{\Gamma}^{mp'}_{p''p'''} S_{q'p'''} \tag{172}$$

then

$$Z_{pm} = U^{p''q'}_{pp'} M^{mp'}_{p''q'} \tag{173}$$

$$F_{4a}^{\mu} = \left(\sum_{m>p} X_{mp}^{\nu} + \sum_{p>m} Y_{pm}^{\nu} \right) Z_{pm} \tag{174}$$

The exchange-dispersion part is much more verbose and lets us look at the most complex part which contains contributions like

$$F_4^{\mu\nu} = \left(\sum_{m>p} X_{mp}^{\mu} + \sum_{p>m} Y_{pm}^{\mu} \right) \left(\sum_{n>q} X_{nq}^{\nu} + \sum_{p>n} Y_{pn}^{\nu} \right) \bar{\Gamma}_{p''p'''}^{mp'} \bar{\Gamma}_{q''q'''}^{nq'} S_{p'q'''} S_{q'p'''} \tilde{v}_{pq}^{p''q''} \tag{175}$$

of which there are sixteen different terms arising from all combinations of the m and n indices in the TPDMs. Let us go about this systematically, and build some common intermediates (inspired by Hapka[S26]). For convenience let us use upper case indices for monomer B and lower case indices for monomer A and lower all indices. Given the finite number of letters in the alphabet, intermediates will often have reuse symbols. For safety, we will have to assume they are only defined locally (i.e. only for one of the 16 terms), although by inspection many of them are identical (up to permutation of indices). Let us first at least define the protected intermediates (similar to Hapka[S26]) which can be used in practice:

$$N_{mqrR}^{A} = \Gamma_{mqrs} S_{sR} \tag{176}$$

$$N_{MQRr}^{B} = \Gamma_{MQRS} S_{rS} \tag{177}$$

$$Z_{mpMP}^{AB} = N_{mqrR} N_{MQRr} \tilde{v}_{pP}^{qQ} \tag{178}$$

$$U_{rsPQ} = \Gamma_{pqrs} \tilde{v}_{pP}^{qQ} \tag{179}$$

$$W_{rsRS}^{AB} = \Gamma_{pqrs} \Gamma_{PQRS} \tilde{v}_{pP}^{qQ} \tag{180}$$

$$= U_{rsPQ} \Gamma_{PQRS}, \tag{181}$$

with obvious analogues if monomer B's indices are contracted over.

$$\Gamma_{mqrs}\Gamma_{MQRS}S_{rS}S_{sR}\tilde{v}_{pqPQ} = N^{A}_{mqrR}N^{B}_{MQRr}\tilde{v}_{pqPQ} \tag{182}$$

$$= T^{AB}_{mqMQ}\tilde{v}_{pqPQ} \tag{183}$$

$$= V^{AB}_{mpMP} \tag{184}$$

then

$$F^{\mu\nu}_{4(1)} = Q^{\mu}_{mp}Q^{\nu}_{MP}V^{AB}_{mpMP} \tag{185}$$

$$= G^{\mu}_{MP}Q^{\nu}_{MP}. \tag{186}$$

Now there is some pattern in this, namely we will have contributions where the contracted Q/R matrix index (m, M) will be in either half of the 2PDM. Let us proceed systematically along μ first then ν. For the second term we have:

$$\Gamma_{pmrs}\Gamma_{MQRS}S_{rS}S_{sR}\tilde{v}_{pqPQ} = N^{A}_{pmrR}N^{B}_{MQRr}\tilde{v}_{pqPQ} \tag{187}$$

$$= T^{AB}_{pmMQ}\tilde{v}_{pqPQ} \tag{188}$$

$$= V^{AB}_{mqMP} \tag{189}$$

$$F^{\mu\nu}_{4(2)} = R^{\mu}_{mq}Q^{\nu}_{MP}V^{AB}_{mqMP} \tag{190}$$

$$= G^{\mu}_{MP}Q^{\nu}_{MP}. \tag{191}$$

For the third term we have:

$$\Gamma_{pqms}\Gamma_{MQRS}S_{rS}S_{sR}\tilde{v}_{pqPQ} = N^{A}_{pqmR}N^{B}_{MQRr}\tilde{v}_{pqPQ} \tag{192}$$

$$= T^{AB}_{mRPQ}N^{B}_{MQRr} \tag{193}$$

$$= V^{AB}_{mrMP} \quad \text{or} \tag{194}$$

$$= U_{rsPQ}(S_{sR}N^{B}_{MQRr}) \tag{195}$$

$$= V^{AB}_{mrMP} \tag{196}$$

$$F^{\mu\nu}_{4(3)} = Q^{\mu}_{mr}Q^{\nu}_{MP}V_{mrMP} \tag{197}$$

$$= G^{\mu}_{MP}Q^{\nu}_{MP}. \tag{198}$$

For the fourth term we have:

$$\Gamma_{pqrm}\Gamma_{MQRS}S_{rS}S_{sR}\tilde{v}_{pqPQ} = N^{A}_{pqSm}N^{B}_{MQsS}\tilde{v}_{pqPQ} \tag{199}$$

$$= T^{AB}_{mSPQ}N^{B}_{MQsS} \tag{200}$$

$$= V^{AB}_{msMP} \tag{201}$$

$$F^{\mu\nu}_{4(4)} = R^{\mu}_{ms}Q^{\nu}_{MP}V^{AB}_{msMP} \tag{202}$$

$$= G^{\mu}_{MP}Q^{\nu}_{MP}. \tag{203}$$

For the fifth term we have:

$$\Gamma_{mqrs}\Gamma_{PMRS}S_{rS}S_{sR}\tilde{v}_{pqPQ} = N^{A}_{mqrR}N^{B}_{PMRr}\tilde{v}_{pqPQ} \tag{204}$$

$$= T^{AB}_{mqMP}\tilde{v}_{pqPQ} \tag{205}$$

$$= V^{AB}_{mpMQ} \tag{206}$$

$$F^{\mu\nu}_{4(5)} = Q^{\mu}_{mp} R^{\nu}_{MQ} V^{AB}_{mpMQ} \tag{207}$$

$$= G^{\mu}_{MQ} R^{\nu}_{MQ}. \tag{208}$$

For the sixth term we have:

$$\Gamma_{pmrs}\Gamma_{PMRS} S_{rS} S_{sR} \tilde{v}_{pqPQ} = N^{A}_{pmrR} N^{B}_{PMRr} \tilde{v}_{pqPQ} \tag{209}$$

$$= T^{AB}_{pmPM} \tilde{v}_{pqPQ} \tag{210}$$

$$= V^{AB}_{mqMQ} \tag{211}$$

$$F^{\mu\nu}_{4(6)} = R^{\mu}_{mq} R^{\nu}_{MQ} V^{AB}_{mqMQ} \tag{212}$$

$$= G^{\mu}_{MQ} R^{\nu}_{MQ}. \tag{213}$$

For the seventh term we have:

$$\Gamma_{pqms}\Gamma_{PMRS} S_{rS} S_{sR} \tilde{v}_{pqPQ} = N^{A}_{pqmR} N^{B}_{PMRr} \tilde{v}_{pqPQ} \tag{214}$$

$$= T^{AB}_{mRPQ} N^{B}_{PMRr} \tag{215}$$

$$= V^{AB}_{mrMQ} \tag{216}$$

$$F^{\mu\nu}_{4(7)} = Q^{\mu}_{mr} R^{\nu}_{MQ} V_{mrMQ} \tag{217}$$

$$= G^{\mu}_{MQ} R^{\nu}_{MQ}. \tag{218}$$

For the eight term we have:

$$\Gamma_{pqrm}\Gamma_{PMRS}S_{rS}S_{sR}\tilde{v}_{pqPQ} = N^{A}_{pqSm}N^{B}_{PMsS}\tilde{v}_{pqPQ} \tag{219}$$

$$= T^{AB}_{mSPQ}N^{B}_{PMsS} \tag{220}$$

$$= V^{AB}_{msMQ} \tag{221}$$

$$F^{\mu\nu}_{4(8)} = R^{\mu}_{ms}R^{\nu}_{MQ}V^{AB}_{msMQ} \tag{222}$$

$$= G^{\mu}_{MQ}Q^{\nu}_{MQ}. \tag{223}$$

For the ninth term we have:

$$\Gamma_{mqrs}\Gamma_{PQMS}S_{rS}S_{sR}\tilde{v}_{pqPQ} = N^{A}_{mqrR}N^{B}_{PQMr}\tilde{v}_{pqPQ} \tag{224}$$

$$= N^{A}_{mqrR}T^{AB}_{Mrpq} \tag{225}$$

$$= V^{AB}_{mpMR} \tag{226}$$

$$F^{\mu\nu}_{4(9)} = Q^{\mu}_{mp}Q^{\nu}_{MR}V^{AB}_{mpMR} \tag{227}$$

$$= G^{\mu}_{MR}Q^{\nu}_{MR}. \tag{228}$$

For the tenth term we have:

$$\Gamma_{pmrs}\Gamma_{PQMS}S_{rS}S_{sR}\tilde{v}_{pqPQ} = N^{A}_{pmrR}N^{B}_{PQMr}\tilde{v}_{pqPQ} \tag{229}$$

$$= N^{A}_{pmrR}T^{AB}_{Mrpq} \tag{230}$$

$$= V^{AB}_{mqMR} \tag{231}$$

$$F^{\mu\nu}_{4(10)} = R^{\mu}_{mq} Q^{\nu}_{MR} V^{AB}_{mqMR} \tag{232}$$

$$= G^{\mu}_{MR} Q^{\nu}_{MR}. \tag{233}$$

For the eleventh term we have:

$$\Gamma_{pqms}\Gamma_{PQMS} S_{rS} S_{sR} \tilde{v}_{pqPQ} = T^{AB}_{msPQ} N^{B}_{PQMr} S_{sR} \tag{234}$$

$$= V^{AB}_{mrMR} \tag{235}$$

$$F^{\mu\nu}_{4(11)} = Q^{\mu}_{mr} Q^{\nu}_{MR} V^{AB}_{mrMR} \tag{236}$$

$$= G^{\mu}_{MR} Q^{\nu}_{MR}. \tag{237}$$

For the twelfth term we have:

$$\Gamma_{pqrm}\Gamma_{PQMS} S_{rS} S_{sR} \tilde{v}_{pqPQ} = T^{AB}_{mrPQ} N^{B}_{PQMr} S_{sR} \tag{238}$$

$$= V^{AB}_{msMR} \tag{239}$$

$$F^{\mu\nu}_{4(12)} = R^{\mu}_{ms} Q^{\nu}_{MR} V^{AB}_{msMR} \tag{240}$$

$$= G^{\mu}_{MR} Q^{\nu}_{MR}. \tag{241}$$

For the thirteenth term we have:

$$\Gamma_{mqrs}\Gamma_{PQRM} S_{rS} S_{sR} \tilde{v}_{pqPQ} = N^{A}_{mqSR} T^{AB}_{pqRM} \tag{242}$$

$$= V^{AB}_{mpMS} \tag{243}$$

$$F^{\mu\nu}_{4(13)} = Q^{\mu}_{mp} R^{\nu}_{MS} V^{AB}_{mpMS} \tag{244}$$

$$= G^{\mu}_{MS} Q^{\nu}_{MS}. \tag{245}$$

For the fourteenth term we have:

$$\Gamma_{pmrs}\Gamma_{PQRM} S_{rS} S_{sR} \tilde{v}_{pqPQ} = N^{A}_{mqSR} T^{AB}_{pqRM} \tag{246}$$

$$= V^{AB}_{mqMS} \tag{247}$$

$$F^{\mu\nu}_{4(14)} = R^{\mu}_{mq} R^{\nu}_{MS} V^{AB}_{mqMS} \tag{248}$$

$$= G^{\mu}_{MS} Q^{\nu}_{MS}. \tag{249}$$

For the fifteenth term we have:

$$\Gamma_{pqms}\Gamma_{PQRM} S_{rS} S_{sR} \tilde{v}_{pqPQ} = \Gamma_{pqms} S_{rS} S_{sR} T^{AB}_{pqRM} \tag{250}$$

$$= U^{AB}_{msRM} S_{rS} S_{sR} \tag{251}$$

$$= V^{AB}_{mrMS} \tag{252}$$

$$F^{\mu\nu}_{4(15)} = Q^{\mu}_{mr} R^{\nu}_{MS} V^{AB}_{mrMS} \tag{253}$$

$$= G^{\mu}_{MS} Q^{\nu}_{MS}. \tag{254}$$

For the sixteenth term we have:

$$\Gamma_{pqrm}\Gamma_{PQRM} S_{rS} S_{sR} \tilde{v}_{pqPQ} = \Gamma_{pqrm} S_{rS} S_{sR} T^{AB}_{pqRM} \tag{255}$$

$$= U^{AB}_{rmRM} S_{rS} S_{sR} \tag{256}$$

$$= V^{AB}_{msMS} \tag{257}$$

$$F^{\mu\nu}_{4(16)} = R^{\mu}_{ms} R^{\nu}_{MS} V^{AB}_{msMS} \tag{258}$$

$$= G^{\mu}_{MS} Q^{\nu}_{MS}. \tag{259}$$

Naturally,

$$F^{\mu\nu}_{4} = \sum_{i} F^{\mu\nu}_{4(i)}. \tag{260}$$

3.5 Relationship of the SAPT expansion to SAPT0

In this section, we to compare the SAPT truncation in this work with the popular SAPT0 single reference method. This work (same as Eq. (4)):

$$E_{SAPT} = E^{(1)}_{\text{elst}} + E^{(1)}_{\text{exch}} + E^{(2)}_{\text{ind,erpa}} + E^{(2)}_{\text{disp,erpa}} + E^{(2)}_{\text{exch-disp,erpa}} + E^{(2)}_{\text{exch-ind,erpa}}, \tag{261}$$

where we added an extra erpa subscript to denote that the transition densities are approximated using ERPA.

The popular SAPT0 is defined as:

$$E_{SAPT0} = E^{(1)}_{\text{elst}} + E^{(1)}_{\text{exch}} + E^{(2)}_{\text{ind,resp}} + E^{(2)}_{\text{disp,u}} + E^{(2)}_{\text{exch-disp}} + E^{(2)}_{\text{exch-ind,resp}} + \delta^{(2)}_{HF}, \tag{262}$$

where the subscript resp denotes that orbital relaxation effects are included via linear response (CP-HF), u indicates uncoupled and

$$\delta^{(2)}_{HF} = E^{\text{HF}}_{\text{int}} - (E^{(1)}_{\text{elst}} + E^{(1)}_{\text{exch}} + E^{(2)}_{\text{ind,resp}} + E^{(2)}_{\text{exch-ind,resp}}), \tag{263}$$

where $E^{\text{HF}}_{\text{int}}$ is supermolecular the Hartree-Fock interaction energy. The addition of $\delta^{(2)}_{HF}$ accounts for higher-order induction effects (see Ref.[S34] and references therein for a detailed derivation of the SAPT0 terms).

There are three key differences in the two expansions:

First, the induction term (and its exchange counterpart) in SAPT0 includes orbital relaxation effects in response to the perturbation field of the other monomer via linear response; in contrast, the induction term in this work uses approximate transition densities from ERPA instead of linear response.

Second, the dispersion term (and its exchange counterpart) in SAPT0 uses an uncoupled approach where the above mentioned orbital response is neglected; this work uses a coupled approach as used in SAPT(DFT) (again using approximated transition densities via ERPA).

Third, SAPT0 accounts for higher order induction effects by including the $\delta_{HF}^{(2)}$ term, such a term is omitted in our work because it would require the VQE calculation of the supersystem. Alternatively, higher order induction effects could be approximated $\delta_{approx}^{(2)}$ using the Hartee-Fock supermolecular binding energy:

$$\delta_{approx}^{(2)} = E_{\mathrm{int}}^{\mathrm{HF}} - (E_{\mathrm{elst}}^{(1)} + E_{\mathrm{exch}}^{(1)} + E_{\mathrm{ind,erpa}}^{(2)} + E_{\mathrm{exch-ind,erpa}}^{(20)}). \tag{264}$$

Hapka *et al.*[S35] suggest this approximation for their SAPT(MC) methods where they face a similar issue. They found that the SAPT(MC) methods for single reference systems (using a very small active space) were more accurate than SAPT0 using the (small) TK21 benchmark set. We confirm this finding for the single reference HNO complexes from the main text: The SAPT expansion with a RHF reference (SAPT(RHF)) performs similar to SAPT0 even without the addition of the higher order induction term $\delta^{(2)}$. The results are summarized in table S3. We chose to omit such an approximate term in this work because extensive numerical testing is required to properly decide this issue. Thus, the three differences in the SAPT expansion and the difference reference wave functions make it difficult to determine an expected accuracy differences between SATP0 and SAPT(VQE) without extensive numerical benchmarks.

4 Computational Details

All density functional theory calculations were performed with the pyscf software package[S36] and used a Lebedev grid used for the numerical integration of the exchange correlation functionals with 70, 105, 140 radial grid points and 590 770, 770 angular grid points, for atoms of the first, second and fourth period respectively (grid level 5). The geometry optimizations were performed with pyscf and the geomeTRIC[S37] package. All CASSCF calculation were performed with pyscf software package[S36] and selected CI (semistochastic heat-bath configuration interaction (SHCI)) calculations with the DICE[S38,S39] program package via the pyscf plugin. We used a loose screening parameter of 0.001 (sweep ϵ) for computational efficiency and in order to afford large active spaces. We used the 6-31G[S40,S41] basis for the "stretched water" dimer and a mixed basis (C: 6-31G; H: STO-3G[S42,S43]) for the benzene p-benzyne dimer. We also employed a mixed basis for the $[Mn(NH_3)_3(CN)_2NO]^0 \cdots X$ (X = HF,H_2O,NH_3 and CH_4), where the crucial moieties were described with the 6-31G basis (Mn, NO and X) and the ligand framework (CN and NH_3) STO-3G was used. The mixed basis was chosen due to the memory constraints of the GPU for the subsequent ERPA calculations. This is because our current implementation is limited in system size due to a non-optimal treatment of core/active/virtual simpliciation and a lack of density fitting of the response functions. Note that all VQE calculations were performed on ideal statevector simulators, which were carried out on an GPU accelerated in-house QC Ware package (quasar/vulcan). In addition, double factorization was used for evaluating the total energy.[S44,S45] All SAPT and VQE calculations were performed on the newest NVIDIA A100 GPUs provided by Amazon Web Services.

The geometries of the water dimer and benzene were taken from the SI of our previous study.[S3] The $[Mn(NH_3)_3(CN)_2NO]^0$ monomer was optimized without constraints and the geometry was frozen for subsequent geometry optimization of the hydrogen bonded dimer complexes. This ensures that the active space orbitals remain as similar as possible in each complex.

The active spaces for the "stretched" water dimer and benzene p-benzyne dimer, were selected based on MP2 natural orbitals followed by a CASSCF calculation with (8e, 8o) and (6e, 6o), respectively. The active space of $[Mn(NH_3)_3(CN)_2NO]^0$ was selected via the automated construction of molecular active spaces from atomic valence orbitals (AVAS),[S17] where we included all Mn 3-d and the p-orbitals of the NO ligand. Additionally, we systematically added orbitals to the active space based on SHCI resulting in a (16e, 22o) active space. The natural occupation numbers are plotted in Fig. S2 and show several orbitals with strong deviation from integer values confirming the multi-reference character of the system. The subsequent CASSCF calculation used the SHCI natural orbitals as a starting guess and included (6e, 6o) in the active space. The corresponding natural orbitals are depicted in Fig. S5. The strongest deviation are seen in Fig. S5 (c)–(f); the orbitals show both bonding and antibonding π type orbitals of Mn-d_{xz} and Mn-d_{yz} with two NO π^* orbitals. These orbitals correspond to metal-to-ligand π backbonding in the Dewar–Chatt–Duncanson picture.[S46,S47] The strong static correlation of these four electrons indicates that the electronic structure of this complex is a superposition of the two configurations [Mn(II)-$NO^\bullet$] and [Mn(II)-NO^+]. The corresponding NOONs are depicted in Fig. S2 and the values only slightly change in comparison to the (approximate) larger active space SHCI results.

In order to converge the k-mUCJ ansatz with larger repetition factors a read-in algorithm inspired by Ref. S48 was implemented. For a given circuit repetition number N, the optimal VQE parameters from the $N-1^{\text{th}}$ step are used as a starting guess and the remaining extra parameters are populated with random parameters from a normal Gaussian distribution ($\mathcal{N}$) with mean $\mu = 0.0$ and variance $\sigma^2 = 0.001$ ($\mathcal{N}(0.0,\ 0.001)$). We increased the repetition factor in the following order: $N = 1, 2, 4, \ldots, m$. All calculations were performed in the dimer-centered basis (ghost atoms on the other monomer) and a gradient threshold of 1×10^{-6} for the L-BFGS-B solver in scipy or with a maximum iteration of 1500 whichever occurred first. In cases when the maximum iteration was reached first we found the norm of the gradient to be $\approx 1 \times 10^{-5}$.

For the subsequent SAPT calculations we constructed the one- and two-particle reduced density matrices in the full set of natural orbitals. In the case of RHF these correspond to the canonical Hartree–Fock orbitals. For SAPT(CASSCF) and SAPT(VQE) we used the CASSCF natural orbitals. These density matrices were then used to construct the ERPA and SAPT equations given in Eq. (69) and Section 3.4. We used a threshold of 1×10^{-5} for the canonical orthogonalization step when solving the ERPA generalized (symmetric) eigenvalue problem. The ERPA and SAPT expressions given in in Eq. (69) and Section 3.4 were implemented in an in-house python code. NumPy[S49,S50] was used for tensor manipulation and linear algebra operations with certain steps accelerated using CuPy[S51]. This prototyping code does not exploit core-active-virtual partitioning and is thus limited to systems containing roughly 130 orbitals.

5 Additional Information for the Results Section

Table S1: Detailed SAPT energy contributions for all system studied in this manuscript from Fig. 2, 3 and 5 from the main manuscript (energies in kcal/mol, intermolecular distance r in Å)

System	Method	$E^{(1)}_{\mathrm{elst}}$	$E^{(1)}_{\mathrm{exch}}$	$E^{(2)}_{\mathrm{disp}}$	$E^{(2)}_{\mathrm{exch-disp}}$	$E^{(2)}_{\mathrm{ind}}$	$E^{(2)}_{\mathrm{exch-ind}}$	E_{int}	r
$H_2O \cdots H_2O$	SAPT(CAS-CI)	-11.41	7.89	-0.88	0.20	-3.57	2.15	-5.63	2.0
$H_2O \cdots H_2O$	SAPT(VQE) (k=1)	-10.89	7.75	-0.90	0.19	-3.33	1.93	-5.25	2.0
$H_2O \cdots H_2O$	SAPT(VQE) (k=4)	-11.40	7.93	-0.88	0.20	-3.53	2.11	-5.58	2.0
p−Bz···Bz	SAPT(CAS-CI)	0.13	0.26	-0.61	0.03	-0.10	0.05	-0.23	3.9
p−Bz···Bz	SAPT(VQE) (k=1)	0.08	0.26	-0.61	0.03	-0.10	0.05	-0.29	3.9
p−Bz···Bz	SAPT(VQE) (k=4)	0.12	0.26	-0.61	0.03	-0.10	0.05	-0.25	3.9
MnNO···HF	SAPT(CAS-CI)	-11.88	5.6	-1.09	0.13	-3.21	1.12	-9.33	1.8
MnNO···HF	SAPT(VQE) (k=1)	-12.19	5.58	-1.08	0.13	-3.23	1.12	-9.67	1.8
MnNO···HF	SAPT(VQE) (k=4)	-11.87	5.60	-1.09	0.13	-3.21	1.12	-9.31	1.8
MnNO···HOH	SAPT(CAS-CI)	-7.59	3.62	-0.86	0.14	-1.60	0.74	-5.55	2.1
MnNO···HOH	SAPT(VQE) (k=1)	-7.78	3.60	-0.85	0.14	-1.61	0.74	-5.76	2.1
MnNO···HOH	SAPT(VQE) (k=4)	-7.6	3.62	-0.85	0.14	-1.6	0.74	-5.56	2.1
$MnNO \cdots HNH_2$	SAPT(CAS-CI)	-2.77	1.31	-0.62	0.06	-0.56	0.20	-2.37	2.3
$MnNO \cdots HNH_2$	SAPT(VQE) (k=1)	-2.83	1.31	-0.61	0.06	-0.57	0.19	-2.45	2.3
$MnNO \cdots HNH_2$	SAPT(VQE) (k=4)	-2.77	1.31	-0.62	0.06	-0.56	0.20	-2.37	2.3
$MnNO \cdots HCH_3$	SAPT(CAS-CI)	-0.10	0.02	-0.13	0.00	-0.07	0.00	-0.27	3.5
$MnNO \cdots HCH_3$	SAPT(VQE) (k=1)	-0.10	0.02	-0.13	0.00	-0.07	0.00	-0.28	3.5
$MnNO \cdots HCH_3$	SAPT(VQE) (k=4)	-0.10	0.02	-0.13	0.00	-0.07	0.00	-0.27	3.5

5.1 Detailed SAPT Analysis of the MnNO Complexes

The detailed analysis of the energy term decomposition helps to unravel the origin of the interaction. Fig. 5 (b) plots each component of the SAPT(VQE) (k = 4) calculation of the series of hydrogen bonded complexes plus the "stretched" water dimer as a reference of a typical hydrogen bond. We see that the electrostatic term dominates the interaction as expected for hydrogen bonds. The MnNO···HF complex has the largest electrostatic contribution (−11.9 kcal/mol) which is stronger than a water hydrogen bond (−11.4 kcal/mol). The electrostatic interaction of MnNO···HOH is significantly weaker despite the fact that the dipole moments of both H−F and H_2O are around 1.8 D. When comparing the exchange terms, we see that the terms rapidly decrease from MnNO···HF to MnNO···HCH_3 which can be rationalized by the change in bond distances and by the exponential decay of this term. Interestingly, the water dimer and MnNO···HF have similar bond distances but MnNO···HF exhibits a significantly less repulsive term. This difference is the main driving force for the difference in interaction energies and can be rationalized by the diffuseness of the lone pairs. The bound NO becomes (partly) NO^+, which makes the lone pair more compact in space than the lone pair in the water dimer; thus, resulting in less exchange repulsion.

5.2 Additional Comments on the Comparison Between DFT and SAPT(VQE)

We compare the SAPT(VQE) interaction energies to DFT based supermolecular (BSSE corrected[S52]) interaction energies. We note that exact comparisons are difficult for two reasons: first, the static correlation makes it difficult to generate reliable reference energies as the "gold standard" CCSD(T) for non-covalent interactions is not reliable anymore;[S53] second, SAPT0 produces most accurate energies with the jun-cc-pVDZ basis set[S2] which cannot be employed due to technical limits in the current implementation. However, nitrosyl complexes are an example of non-universality problem of approximate density functionals as the hydrogen bond-

ing moiety and the nitrosyl moiety prefer different approximate density functionals[S54–S56] and thus reliable prediction are only possible with careful system specific benchmarking when experimental data is available.[S57] In contrast, SAPT is expected to give accurate results for hydrogen bonds given that proper monomer wavefunctions are used. The calculation of accurate interaction energies poses a challenge for DFT as different types of approximate density functionals are recommended for the different molecular moieties: non-hybrid functionals are recommended for an accurate description of the metal-NO moiety,[S54,S55] while for hydrogen bonds range-separated hybrids are recommended.[S58] These two competing requirements make these systems very sensitive to the choice of specific approximate exchange correlation functional illustrating the non-universality problem of approximate density functionals. When using DFT, careful benchmark against experimental data is necessary.[S57] Our findings indicate that the k-uCJ VQE ansatz is able to accurately describe the difficult electronic structure of the nitrosyl monomer in the hydrogen bonding complex (see Fig. S6) To illustrate this point, Fig. 6 (main text) plots the interaction energies of SAPT(VQE), SAPT(CAS-CI) and several popular DFT functionals (supramolecular). We included many popular functionals as well as several top performing functionals for non-covalent interactions.[S58] We see in Fig. 6 (main text) that the SAPT(VQE) (k = 4) is almost identical to the SAPT(CAS-CI) in all four cases. We also note that the DFT functionals exhibit a significant spread for each complex. The B97-D functional predicted the smallest binding energy in all four cases, but the highest interaction energy is predicted by a different functional. Furthermore, we see the relative ordering of the functionals change for each system (color sequence in each plot). This illustrates the non-universality problem for approximate exchange correlation functionals even for very similar nitrosyl complexes (this also holds true for larger basis set as illustrated in Fig. S8). We note that the SAPT(CAS-CI) results are the reference for the SAPT(VQE) calculations and do *not* represent the true interaction energy, thus, only the SAPT(VQE) not the DFT interaction energies should be compared against this reference. B97-D,[S59] BP86,[S60,S61] SCAN,[S62] PBE,[S63] M06-L,[S64] TPSSh,[S65] B3LYP,[S61,S66,S67] PBE0,[S68]

MN15,[S69] PWB6K,[S70] CAM-B3LYP,[S71] ωB97X-D.[S72] This diverse list includes local DFT functionals, hybrid functionals with a wide range of exact exchange (10%–46%) and range separated hybrids.

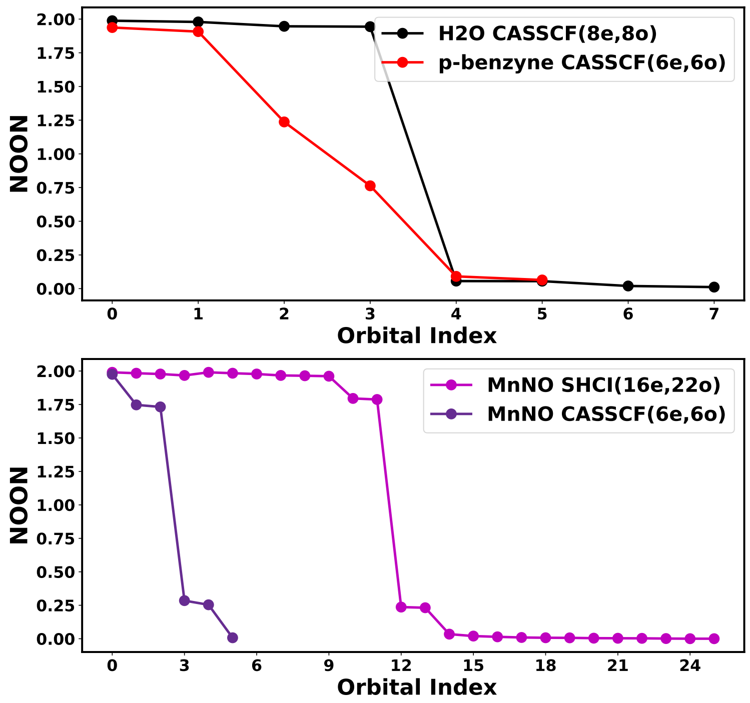

Figure S2: Natural occupation number of the SHCI/CASSCF natural orbitals for the strongly correlated monomer of each test case: upper panel "stretched" H_2O and p-benzyne; lower panel: $[Mn(CN)_2(NH_3)_3NO]^0$.

Table S2: Quantum hardware resource requirements with respect to the repetition factor k (Number of qubits, number of two qubit gates (2-Q-G), number of parameter, quantum circuit depth) for the VQE simulations using the k-muCJ ansatz for the simulation of the $[Mn(CN)_2(NH_3)_3NO]^0$ with an (6e, 6o) active space.

k	1	2	3	4	5	6	7	8
# Qubits	12	12	12	12	12	12	12	12
# 2-Q-G	435	750	1065	1380	1695	2010	2325	2640
# Param.	45	75	105	135	165	195	225	255
Depth	216	378	540	702	864	1026	1188	1350

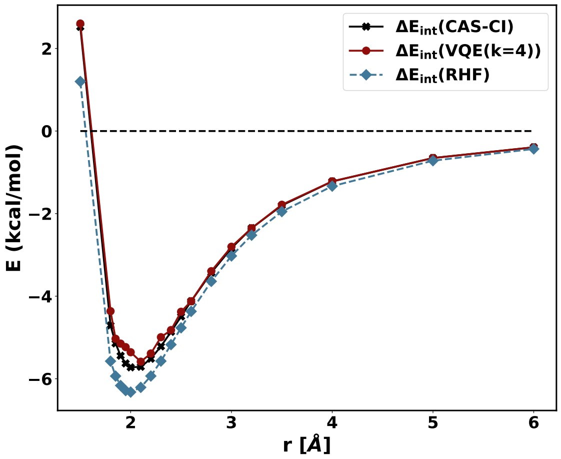

(a)

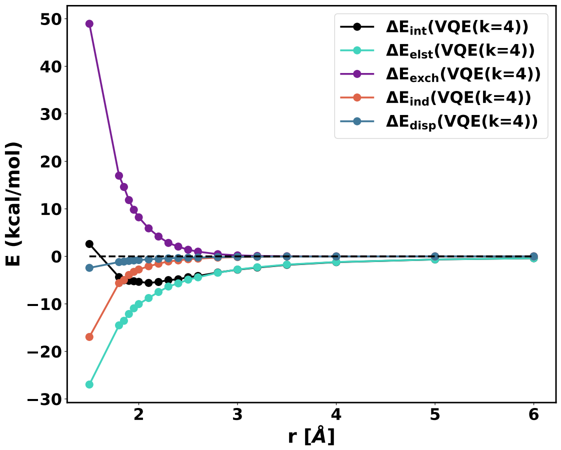

(b)

Figure S3: (a) potential energy scan along r using SAPT(CAS-CI), SAPT(RHF) and SAPT(4-uCJ); (b) distance dependence of each SAPT(4-uCJ) energy term with respect to the intermolecular distance r for the "stretched" water dimer.

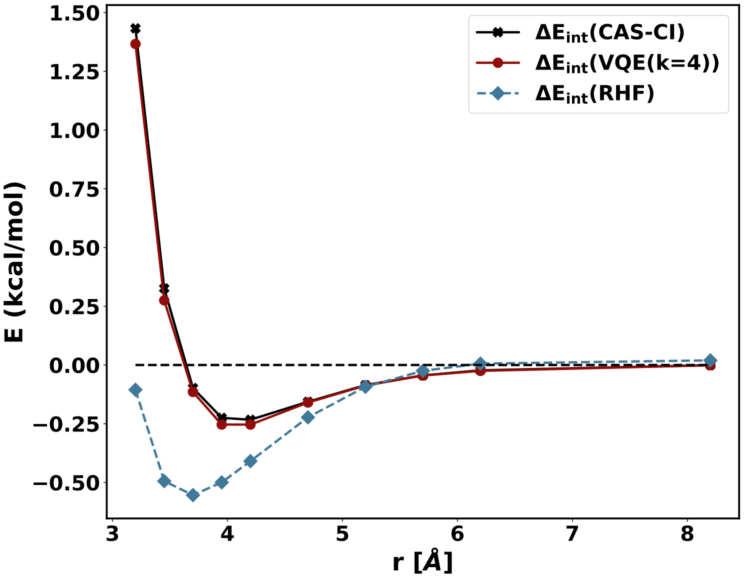

Figure S4: Potential energy scan along r using SAPT(CAS-CI), SAPT(RHF) and SAPT(VQE) ($k = 4$) for the p-benzyne-benzene dimer.

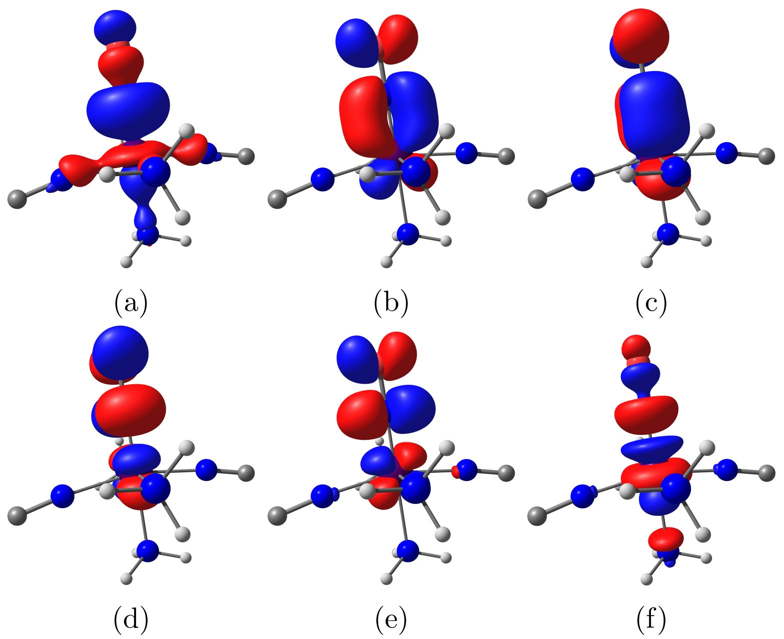

Figure S5: Natural orbitals of the (6e, 6o) CASSCF calculation of $[Mn(CN)_2(NH_3)_3NO]^0$.

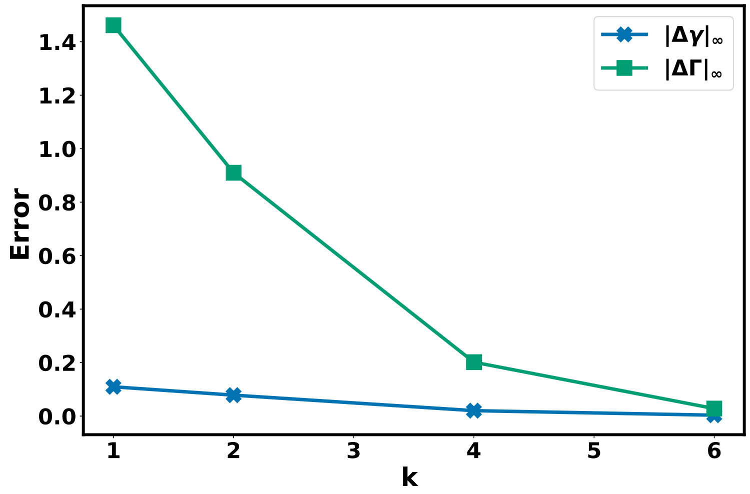

Figure S6: Error in the one and two particle density matrix as a function of the circuit repetition factor k for the $[Mn(CN)_2(NH_3)_3NO]^0$ monomer. The errors are defined as $|\Delta\gamma|_\infty = |\gamma_{\text{CASCI}} - \gamma_{k-\text{muCJ}}|_\infty$ and $|\Delta\Gamma|_\infty = |\Gamma_{\text{CASCI}} - \Gamma_{k-\text{muCJ}}|_\infty$.

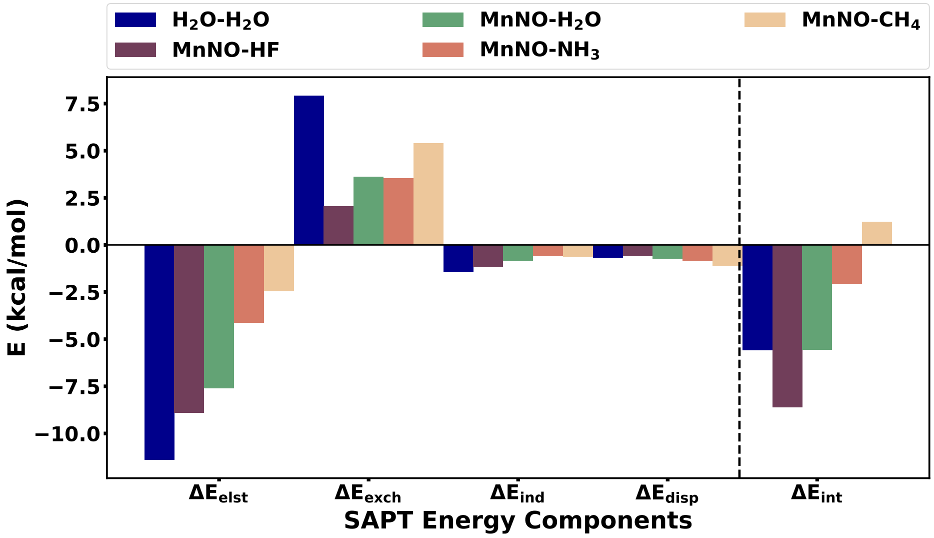

Figure S7: Term-by-term decomposition of the SAPT(4-uCJ) binding energies of each heme-nitrosyls hydrogen complex at a fixed bond distance of r(O−H) = 2.08 Å (equilibrium distance of the H_2O complex).

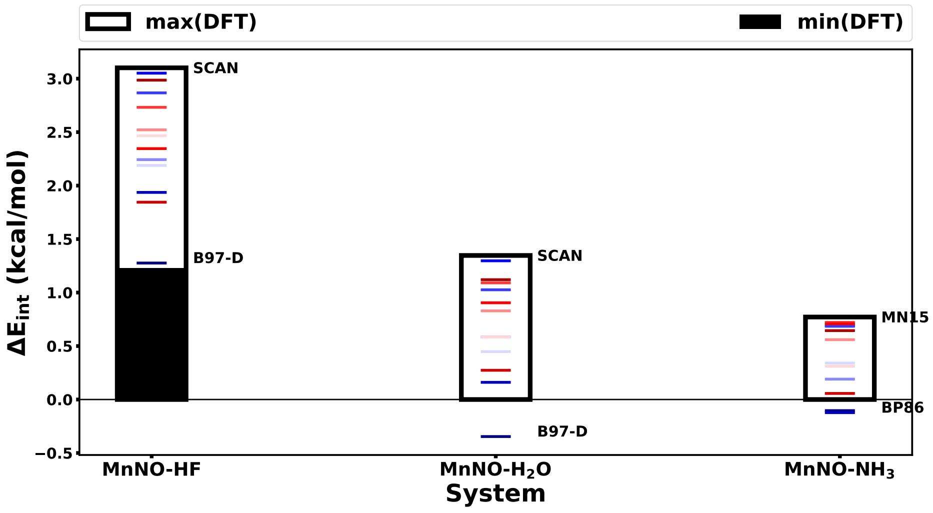

Figure S8: Supramolecuar DFT interaction energies in the def2-TZVPD[S73] basis for the HF, H_2O and NH_3 nitrosyl hydrogen complexes.

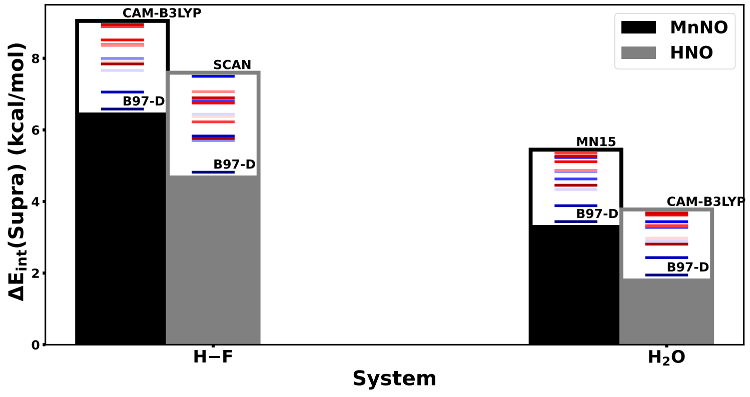

(a)

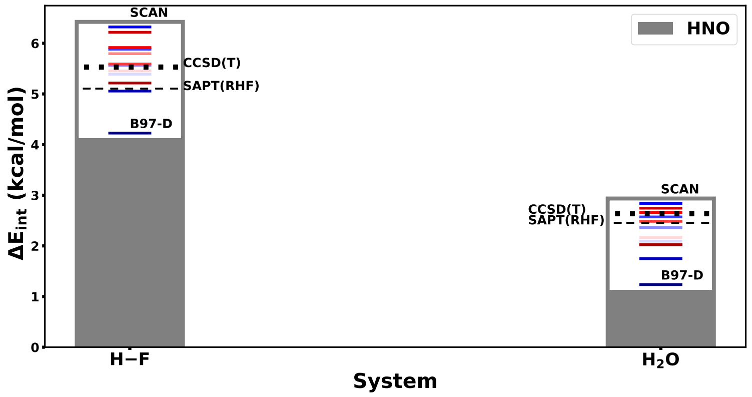

(b)

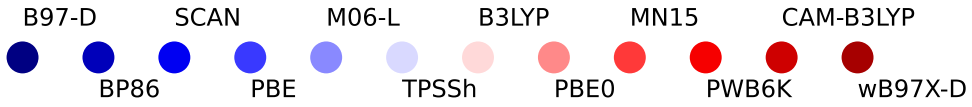

(c)

Figure S9: (a) Comparison between supramolecular DFT interaction energies for the nitrosyl and azanone hydrogen complexes (small basis); (b) Comparison between supramolecular DFT and SAPT in their most common basis sets using the azanone hydrogen complexes. High level CCSD(T) reference interaction energies are also provided for reference (DFT: def2-TZVPD & BSSE corrected; SAPT: SAPT in the jun-cc-pVDZ basis (skips the VQE calculation and uses the RHF wave function instead) and CCSD(T): aug-cc-pVQZ & BSSE corrected; for the DFT functional color coding see (c)).

Table S3: Detailed interaction energies from Fig. 6 (main text) using the supramolecular approach for DFT and CCSD(T) calculation (BSSE corrected). CCSD(T)/aug-cc-pVQZ can be considered very close to the true E_{int} using both a very accurate wave function ansatz (for non strongly correlated systems) and large basis set; DFT calculations using the def2-TZVPD basis set as recommended by practical best practices from Ref. S74 and SAPT calculation use the jun-cc-pVDZ basis set as recommended by Ref. S2; SAPT0 calculation were performed with the Psi4 package.[S75]

System	Basis	B97-D	BP86	SCAN	PBE	M06-L	TPSSh	B3LYP	PBE0	MN15	PWB6K	CAM-B3LYP	ωB97X-D
MnNO···HF	mixed	-6.58	-7.06	-8.37	-7.88	-7.99	-7.66	-7.91	-8.36	-8.89	-8.51	-8.94	-7.84
MnNO···HOH	mixed	-3.44	-3.88	-5.23	-4.63	-4.83	-4.33	-4.45	-4.87	-5.35	-5.11	-5.26	-4.45
HNO···HF	6-31G	-4.82	-5.83	-7.5	-6.81	-5.7	-6.44	-6.38	-7.07	-6.22	-6.75	-6.9	-5.76
H_2O···H_2O	6-31G	-1.95	-2.43	-3.44	-3.28	-2.81	-2.9	-2.98	-3.34	-3.31	-3.62	-3.68	-2.81
HNO···HF	def2-TZVPD	-4.23	-5.06	-6.32	-5.88	-5.57	-5.39	-5.45	-5.79	-5.59	-5.92	-6.22	-5.22
HNO···H_2O	def2-TZVPD	-1.24	-1.75	-2.84	-2.57	-2.36	-2.09	-2.17	-2.49	-2.49	-2.66	-2.75	-2.02

System	CCSD(T) (Basis)	SAPT(RHF) (Basis)	SAPT0 (Basis)
HNO···HF	-5.5 (aug-cc-pVQZ)	-5.1 (jun-cc-pVDZ)	-6.1 (jun-cc-pVDZ)
HNO···H_2O	-2.6 (aug-cc-pVQZ)	-2.4 (jun-cc-pVDZ)	-2.9 (jun-cc-pVDZ)

References

(S1) Jeziorski, B.; Moszynski, R.; Szalewicz, K. Perturbation Theory Approach to Intermolecular Potential Energy Surfaces of van der Waals Complexes. *Chem. Rev.* **1994**, *94*, 1887–1930.

(S2) Parker, T. M.; Burns, L. A.; Parrish, R. M.; Ryno, A. G.; Sherrill, C. D. Levels of symmetry adapted perturbation theory (SAPT). I. Efficiency and performance for interaction energies. *J. Chem. Phys.* **2014**, *140*, 094106.

(S3) Malone, F. D.; Parrish, R. M.; Welden, A. R.; Fox, T.; Degroote, M.; Kyoseva, E.; Moll, N.; Santagati, R.; Streif, M. Towards the simulation of large scale protein-ligand interactions on nisq-era quantum computers. *Chem. Sci.* **2022**,

(S4) Moszynski, R.; Jeziorski, B.; Rybak, S.; Szalewicz, K.; Williams, H. L. Many-body theory of exchange effects in intermolecular interactions. Density matrix approach and applications to He-F-, He-HF, H2-HF, and Ar-H2 dimers. *J. Chem. Phys.* **1994**, *100*, 5080.

(S5) Korona, T. First-order exchange energy of intermolecular interactions from coupled cluster density matrices and their cumulants. *J. Chem. Phys.* **2008**, *128*, 224104.

(S6) Korona, T. Second-order exchange-induction energy of intermolecular interactions from coupled cluster density matrices and their cumulants. *Phys. Chem. Chem. Phys.* **2008**, *10*, 6509–6519.

(S7) Korona, T. Exchange-Dispersion Energy: A Formulation in Terms of Monomer Properties and Coupled Cluster Treatment of Intramonomer Correlation. *J. Chem. Theory Comput.* **2009**, *5*, 2663–2678.

(S8) Hapka, M.; Przybytek, M.; Pernal, K. Symmetry-Adapted Perturbation Theory Based

on Multiconfigurational Wave Function Description of Monomers. *J. Chem. Theory Comput.* **2021**, *17*, 5538–5555.

(S9) Parrish, R. M.; McMahon, P. L. Quantum filter diagonalization: Quantum eigendecomposition without full quantum phase estimation. *arXiv preprint arXiv:1909.08925* **2019**,

(S10) Parrish, R. M.; Hohenstein, E. G.; McMahon, P. L.; Martínez, T. J. Quantum Computation of Electronic Transitions Using a Variational Quantum Eigensolver. *Phys. Rev. Lett.* **2019**, *122*, 230401.

(S11) Cai, X.; Fang, W.-H.; Fan, H.; Li, Z. Quantum computation of molecular response properties. *Phys. Rev. Res.* **2020**, *2*, 033324.

(S12) Ollitrault, P. J.; Kandala, A.; Chen, C.-F.; Barkoutsos, P. K.; Mezzacapo, A.; Pistoia, M.; Sheldon, S.; Woerner, S.; Gambetta, J. M.; Tavernelli, I. Quantum equation of motion for computing molecular excitation energies on a noisy quantum processor. *Phys. Rev. Res.* **2020**, *2*, 043140.

(S13) Chatterjee, K.; Pernal, K. Excitation energies from extended random phase approximation employed with approximate one- and two-electron reduced density matrices. *J. Chem. Phys.* **2012**, *137*, 204109.

(S14) McClean, J. R.; Kimchi-Schwartz, M. E.; Carter, J.; De Jong, W. A. Hybrid quantum-classical hierarchy for mitigation of decoherence and determination of excited states. *Phys. Rev. A* **2017**, *95*, 042308.

(S15) Veryazov, V.; Malmqvist, P. A.; Roos, B. O. How to select active space for multiconfigurational quantum chemistry? *Int. J. Quantum Chem.* **2011**, *111*, 3329–3338.

(S16) Sun, Q.; Chan, G. K.-L. Exact and optimal quantum mechanics/molecular mechanics boundaries. *J. Chem. Theory Comput.* **2014**, *10*, 3784–3790.

(S17) Sayfutyarova, E. R.; Sun, Q.; Chan, G. K.-L.; Knizia, G. Automated construction of molecular active spaces from atomic valence orbitals. *J. Chem. Theory Comput.* **2017**, *13*, 4063–4078.

(S18) Anselmetti, G.-L. R.; Wierichs, D.; Gogolin, C.; Parrish, R. M. Local, expressive, quantum-number-preserving VQE ansätze for fermionic systems. *New J. Phys.* **2021**, *23*, 113010.

(S19) Matsuzawa, Y.; Kurashige, Y. Jastrow-type Decomposition in Quantum Chemistry for Low-Depth Quantum Circuits. *J. Chem. Theory Comput.* **2020**, *16*, 944–952.

(S20) Kivlichan, I. D.; McClean, J.; Wiebe, N.; Gidney, C.; Aspuru-Guzik, A.; Chan, G. K.-L.; Babbush, R. Quantum Simulation of Electronic Structure with Linear Depth and Connectivity. *Phys. Rev. Lett.* **2018**, *120*, 110501.

(S21) O'Gorman, B.; Huggins, W. J.; Rieffel, E. G.; Whaley, K. B. Generalized swap networks for near-term quantum computing. *arXiv preprint arXiv:1905.05118* **2019**,

(S22) Google AI,; Collaborators, Hartree-Fock on a superconducting qubit quantum computer. *Science* **2020**, *369*, 1084–1089.

(S23) Tilly, J.; Sriluckshmy, P. V.; Patel, A.; Fontana, E.; Rungger, I.; Grant, E.; Anderson, R.; Tennyson, J.; Booth, G. H. Reduced density matrix sampling: Self-consistent embedding and multiscale electronic structure on current generation quantum computers. *Phys. Rev. Res.* **2021**, *3*, 033230.

(S24) Korona, T. Second-order exchange-induction energy of intermolecular interactions from coupled cluster density matrices and their cumulants. *Phys. Chem. Chem. Phys.* **2008**, *10*, 6509–6519.

(S25) Korona, T. Exchange-Dispersion Energy: A Formulation in Terms of Monomer Prop-

erties and Coupled Cluster Treatment of Intramonomer Correlation. *J. Chem. Theory Comput.* **2009**, *5*, 2663–2678.

(S26) Hapka, M.; Przybytek, M.; Pernal, K. Second-Order Exchange-Dispersion Energy Based on a Multireference Description of Monomers. *J. Chem. Theory Comput.* **2019**, *15*, 6712–6723.

(S27) Hapka, M.; Przybytek, M.; Pernal, K. Second-order dispersion energy based on multireference description of monomers. *J. Chem. Theory Comput.* **2018**, *15*, 1016–1027.

(S28) van Aggelen, H.; Verstichel, B.; Acke, G.; Degroote, M.; Bultinck, P.; Ayers, P. W.; Van Neck, D. Extended random phase approximation method for atomic excitation energies from correlated and variationally optimized second-order density matrices. *Comput. Theor. Chem* **2013**, *1003*, 50–54.

(S29) Pernal, K.; Chatterjee, K.; Kowalski, P. H. How accurate is the strongly orthogonal geminal theory in predicting excitation energies? Comparison of the extended random phase approximation and the linear response theory approaches. *J. Chem. Phys.* **2014**, *140*, 014101.

(S30) Pernal, K. Electron correlation from the adiabatic connection for multireference wave functions. *Phys. Rev. Lett.* **2018**, *120*, 013001.

(S31) Pastorczak, E.; Pernal, K. Correlation energy from the adiabatic connection formalism for complete active space wave functions. *J. Chem. Theory Comput.* **2018**, *14*, 3493–3503.

(S32) Helgaker, T.; Jorgensen, P.; Olsen, J. *Molecular electronic-structure theory*; John Wiley & Sons: New York, 2014.

(S33) Pernal, K. Intergeminal correction to the antisymmetrized product of strongly or-

thogonal geminals derived from the extended random phase approximation. *J. Chem. Theory Comput.* **2014**, *10*, 4332–4341.

(S34) Hohenstein, E. G.; Sherrill, C. D. Density fitting of intramonomer correlation effects in symmetry-adapted perturbation theory. *J. Chem. Phys.* **2010**, *133*, 014101.

(S35) Hapka, M.; Przybytek, M.; Pernal, K. Symmetry-adapted perturbation theory based on multiconfigurational wave function description of monomers. *J. Chem. Theory Comput.* **2021**, *17*, 5538–5555.

(S36) Sun, Q.; Zhang, X.; Banerjee, S.; Bao, P.; Barbry, M.; Blunt, N. S.; Bogdanov, N. A.; Booth, G. H.; Chen, J.; Cui, Z.-H., et al. Recent developments in the PySCF program package. *J. Chem. Phys.* **2020**, *153*, 024109.

(S37) Wang, L.-P.; Song, C. Geometry optimization made simple with translation and rotation coordinates. *J. Chem. Phys.* **2016**, *144*, 214108.

(S38) Holmes, A. A.; Tubman, N. M.; Umrigar, C. Heat-bath configuration interaction: An efficient selected configuration interaction algorithm inspired by heat-bath sampling. *J. Chem. Theory Comput.* **2016**, *12*, 3674–3680.

(S39) Sharma, S.; Holmes, A. A.; Jeanmairet, G.; Alavi, A.; Umrigar, C. J. Semistochastic heat-bath configuration interaction method: Selected configuration interaction with semistochastic perturbation theory. *J. Chem. Theory Comput.* **2017**, *13*, 1595–1604.

(S40) Hehre, W. J.; Ditchfield, R.; Pople, J. A. Self-Consistent Molecular Orbital Methods. XII. Further Extensions of Gaussian-Type Basis Sets for Use in Molecular Orbital Studies of Organic Molecules. *J. Chem. Phys.* **1972**, *56*, 2257–2261.

(S41) Rassolov, V. A.; Pople, J. A.; Ratner, M. A.; Windus, T. L. 6-31G* basis set for atoms K through Zn. *J. Chem. Phys.* **1998**, *109*, 1223–1229.

(S42) Hehre, W. J.; Stewart, R. F.; Pople, J. A. self-consistent molecular-orbital methods. i. use of gaussian expansions of Slater-type atomic orbitals. *J. Chem. Phys.* **1969**, *51*, 2657–2664.

(S43) Pietro, W. J.; Hehre, W. J. Molecular orbital theory of the properties of inorganic and organometallic compounds. 3. STO-3G basis sets for first-and second-row transition metals. *J. Comput. Chem.* **1983**, *4*, 241–251.

(S44) Motta, M.; Ye, E.; McClean, J. R.; Li, Z.; Minnich, A. J.; Babbush, R.; Chan, G. K. Low rank representations for quantum simulation of electronic structure. *npj Quantum Information* **2021**, *7*, 1–7.

(S45) Huggins, W. J.; McClean, J. R.; Rubin, N. C.; Jiang, Z.; Wiebe, N.; Whaley, K. B.; Babbush, R. Efficient and noise resilient measurements for quantum chemistry on near-term quantum computers. *npj Quantum Information* **2021**, *7*, 1–9.

(S46) Chatt, J.; Duncanson, L. 586. Olefin co-ordination compounds. Part III. Infra-red spectra and structure: attempted preparation of acetylene complexes. *Journal of the Chemical Society (Resumed)* **1953**, 2939–2947.

(S47) Chatt, J.; Duncanson, L.; Venanzi, L. Directing effects in inorganic substitution reactions. Part I. A hypothesis to explain the trans-effect. *Journal of the Chemical Society (Resumed)* **1955**, 4456–4460.

(S48) Huggins, W. J.; Lee, J.; Baek, U.; O'Gorman, B.; Whaley, K. B. A non-orthogonal variational quantum eigensolver. *New J. Phys.* **2020**, *22*, 073009.

(S49) Van Der Walt, S.; Colbert, S. C.; Varoquaux, G. The NumPy array: a structure for efficient numerical computation. *Computing in science & engineering* **2011**, *13*, 22–30.

(S50) Harris, C. R.; Millman, K. J.; Van Der Walt, S. J.; Gommers, R.; Virtanen, P.; Cour-

napeau, D.; Wieser, E.; Taylor, J.; Berg, S.; Smith, N. J., et al. Array programming with NumPy. *Nature* **2020**, *585*, 357–362.

(S51) Nishino, R.; Loomis, S. H. C. Cupy: A numpy-compatible library for nvidia gpu calculations. *31st confernce on neural information processing systems* **2017**, *151*.

(S52) Boys, S. F.; Bernardi, F. The calculation of small molecular interactions by the differences of separate total energies. Some procedures with reduced errors. *Mol. Phys.* **1970**, *19*, 553–566.

(S53) Bulik, I. W.; Henderson, T. M.; Scuseria, G. E. Can single-reference coupled cluster theory describe static correlation? *J. Chem. Theory Comput.* **2015**, *11*, 3171–3179.

(S54) Radon, M.; Pierloot, K. Binding of CO, NO, and O2 to heme by density functional and multireference ab initio calculations. *J. Phys. Chem. A* **2008**, *112*, 11824–11832.

(S55) Radon, M.; Broclawik, E.; Pierloot, K. Electronic structure of selected {FeNO} 7 complexes in heme and non-heme architectures: A density functional and multireference ab initio study. *J. Phys. Chem. B* **2010**, *114*, 1518–1528.

(S56) Mardirossian, N.; Head-Gordon, M. ωB97X-V: A 10-parameter, range-separated hybrid, generalized gradient approximation density functional with nonlocal correlation, designed by a survival-of-the-fittest strategy. *Phys. Chem. Chem. Phys.* **2014**, *16*, 9904–9924.

(S57) Lehnert, N.; Scheidt, W. R.; Wolf, M. W. *Nitrosyl Complexes in Inorganic Chemistry, Biochemistry and Medicine II*; Springer: United States, 2013; pp 155–223.

(S58) Mardirossian, N.; Head-Gordon, M. Thirty years of density functional theory in computational chemistry: an overview and extensive assessment of 200 density functionals. *Mol. Phys.* **2017**, *115*, 2315–2372.

(S59) Grimme, S. Semiempirical GGA-type density functional constructed with a long-range dispersion correction. *J. Comput. Chem.* **2006**, *27*, 1787–1799.

(S60) Perdew, J. P.; Yue, W. Accurate and simple density functional for the electronic exchange energy: Generalized gradient approximation. *Phys. Rev. B* **1986**, *33*, 8800.

(S61) Becke, A. D. Density-functional exchange-energy approximation with correct asymptotic behavior. *Phys. Rev. A* **1988**, *38*, 3098.

(S62) Sun, J.; Ruzsinszky, A.; Perdew, J. P. Strongly constrained and appropriately normed semilocal density functional. *Phys. Rev. Lett.* **2015**, *115*, 036402.

(S63) Perdew, J. P.; Burke, K.; Ernzerhof, M. Generalized gradient approximation made simple. *Phys. Rev. Lett.* **1996**, *77*, 3865.

(S64) Zhao, Y.; Truhlar, D. G. A new local density functional for main-group thermochemistry, transition metal bonding, thermochemical kinetics, and noncovalent interactions. *J. Chem. Phys.* **2006**, *125*, 194101.

(S65) Staroverov, V. N.; Scuseria, G. E.; Tao, J.; Perdew, J. P. Comparative assessment of a new nonempirical density functional: Molecules and hydrogen-bonded complexes. *J. Chem. Phys.* **2003**, *119*, 12129–12137.

(S66) Lee, C.; Yang, W.; Parr, R. G. Development of the Colle-Salvetti correlation-energy formula into a functional of the electron density. *Phys. Rev. B* **1988**, *37*, 785.

(S67) Becke, A. D. Density-functional thermochemistry. III. The role of exact exchange. *J. Chem. Phys.* **1993**, *98*, 5648–5652.

(S68) Adamo, C.; Barone, V. Toward reliable density functional methods without adjustable parameters: The PBE0 model. *J. Chem. Phys.* **1999**, *110*, 6158–6170.

(S69) Haoyu, S. Y.; He, X.; Li, S. L.; Truhlar, D. G. MN15: A Kohn–Sham global-hybrid exchange–correlation density functional with broad accuracy for multi-reference and single-reference systems and noncovalent interactions. *Chem. Sci.* **2016**, *7*, 5032–5051.

(S70) Zhao, Y.; Truhlar, D. G. Design of density functionals that are broadly accurate for thermochemistry, thermochemical kinetics, and nonbonded interactions. *J. Phys. Chem. A* **2005**, *109*, 5656–5667.

(S71) Yanai, T.; Tew, D. P.; Handy, N. C. A new hybrid exchange–correlation functional using the Coulomb-attenuating method (CAM-B3LYP). *Chem. Phys. Lett.* **2004**, *393*, 51–57.

(S72) Chai, J.-D.; Head-Gordon, M. Long-range corrected hybrid density functionals with damped atom–atom dispersion corrections. *Phys. Chem. Chem. Phys.* **2008**, *10*, 6615–6620.

(S73) Weigend, F.; Ahlrichs, R. Balanced basis sets of split valence, triple zeta valence and quadruple zeta valence quality for H to Rn: Design and assessment of accuracy. *Phys. Chem. Chem. Phys.* **2005**, *7*, 3297–3305.

(S74) Bursch, M.; Mewes, J.-M.; Hansen, A.; Grimme, S. Best Practice DFT Protocols for Basic Molecular Computational Chemistry. *ChemRxiv* **2022**,

(S75) Smith, D. G.; Burns, L. A.; Simmonett, A. C.; Parrish, R. M.; Schieber, M. C.; Galvelis, R.; Kraus, P.; Kruse, H.; Di Remigio, R.; Alenaizan, A., et al. PSI4 1.4: Open-source software for high-throughput quantum chemistry. *J. Chem. Phys.* **2020**, *152*, 184108.
